# Geometry-Complete Diffusion for 3D Molecule Generation

**Published:** 2023-02-15

**Authors:** Alex Morehead, Jianlin Cheng

**Affiliations:** Department of Electrical Engineering & Computer Science, University of Missouri, Columbia, MO 65211, USA

## Abstract

Denoising diffusion probabilistic models (DDPMs) (Ho et al. (2020)) have recently taken the field of generative modeling by storm, pioneering new state-of-the-art results in disciplines such as computer vision and computational biology for diverse tasks ranging from text-guided image generation (Ramesh et al. (2022); Saharia et al. (2022); Rombach et al. (2022)) to structure-guided protein design (Ingraham et al. (2022); Watson et al. (2022)). Along this latter line of research, methods such as those of Hoogeboom et al. (2022) have been proposed for unconditionally generating 3D molecules using equivariant graph neural networks (GNNs) within a DDPM framework. Toward this end, we propose GCDM, a geometry-complete diffusion model that achieves new state-of-the-art results for 3D molecule diffusion generation by leveraging the representation learning strengths offered by GNNs that perform geometry-complete message-passing. Our results with GCDM also offer preliminary insights into how physical inductive biases impact the generative dynamics of molecular DDPMs. The source code, data, and instructions to train new models or reproduce our results are freely available at https://github.com/BioinfoMachineLearning/Bio-Diffusion.

## 1 Introduction

Generative modeling has recently been experiencing a renaissance in modeling efforts driven largely by denoising diffusion probabilistic models (DDPMs). At a high level, DDPMs are trained by learning how to denoise a noisy version of an input example. For example, in the context of computer vision, Gaussian noise may be successively added to an input image, and, with the goals of a DDPM in mind, we would desire for a generative model of images to learn how to successfully distinguish between the original input image’s feature signal and the noise signal added to the image thereafter. If a model can achieve such outcomes, we can use the model to generate novel images by first sampling multivariate Gaussian noise and then iteratively removing from the current state of the image the noise predicted by our model. This classic formulation of DDPMs has achieved significant results in the space of image generation (Rombach et al. (2022)), audio synthesis (Kong et al. (2020)), and even meta-learning by learning how to conditionally generate neural network checkpoints (Peebles et al. (2022)). Furthermore, such an approach to generative modeling has expanded its reach to encompass scientific disciplines such as computational biology (Anand & Achim (2022)), computational chemistry (Xu et al. (2022)), and even computational physics (Mudur & Finkbeiner (2022)).

Concurrently, the field of geometric deep learning (GDL) (Bronstein et al. (2021)) has seen a sizeable increase in research interest lately, driven largely by theoretical advances within the discipline (Joshi et al. (2023)) as well as by novel applications of such methodology (Stärk et al. (2022)). Notably, such applications even include what is considered by many researchers to be a solution to the problem of predicting 3D protein structures from their corresponding amino acid sequences (Jumper et al. (2021)). Such an outcome arose, in part, from recent advances in sequence-based language modeling efforts (Vaswani et al. (2017)) as well as from innovations in equivariant neural network modeling (Thomas et al. (2018)).

With such diverse, successful use cases of DDPMs and GDL in mind, in this work, we explore the intersection of geometric graph representation learning and DDPMs to answer the following questions.

- What is the impact of geometric representation learning on DDPMs designed to generate molecular data?
- What are the limitations of current equivariant graph neural networks empowering contemporary molecular DDPMs?
- What role do physical inductive biases play within the generative denoising of molecular DDPMs?

## 2 Related Work

### Generative Modeling.

The field of deep generative modeling (Ruthotto & Haber (2021)) has pioneered a variety of techniques by which to train deep neural networks to create new content similar to that of an existing data repository (e.g., a text dataset of English sentences). Language models such as GPT-3 and ChatGPT (Brown et al. (2020); Schulman et al. (2022)) have become known as hallmark examples of successful generative modeling of text data. In the domains of computer vision and computational biology, techniques such as latent diffusion (Rombach et al. (2022)) and equivariant graph diffusion (Luo et al. (2022)) have established some of the latest state-of-the-art results in generative modeling of images and biomolecules such as proteins, respectively.

### Geometric Deep Learning.

Data residing in a geometric or physical space (e.g., ${\mathbb{R}}^{3}$) can be processed by machine learning algorithms in a plethora of ways. However, in recent years, the field of geometric deep learning has become known for its proficiency in introducing powerful new deep learning methods designed specifically to process geometric data efficiently (Cao et al. (2020)). Examples of popular GDL algorithms include convolutional neural networks designed for working with image data (LeCun et al. (1995)), recurrent neural networks for processing sequence-based data (Medsker & Jain (1999)), and graph neural networks for handling graph-structured model inputs (Zhou et al. (2020)).

### Equivariant Neural Networks.

To process geometric data efficiently, however, recent GDL research (Cohen & Welling (2016); Bronstein et al. (2021); Bulusu et al. (2021)) has specifically shown that designing one’s machine learning algorithm to be equivariant to the symmetry groups the input data points naturally respect (e.g., 3D rotation symmetries) often helps such an algorithm generalize to datasets beyond those used for its cross-validation (e.g., training and testing datasets). As a particularly relevant example of a neural network that is equivariant to several important and common symmetry groups of geometric data, equivariant graph neural networks (Fuchs et al. (2020); Satorras et al. (2021b); Kofinas et al. (2021); Morehead & Cheng (2022)) that are translation and rotation equivariant to inputs residing in ${\mathbb{R}}^{3}$ have become known as hallmark examples of geometric deep learning algorithms that generalize remarkably well to new inputs and require notably fewer training iterations to converge.

### Representation Learning of Scientific Data.

Scientific data, in particular, requires careful consideration in the context of representation learning. As much scientific data contains within it a notion of geometry or latent structure, equivariance has become a key algorithmic component for processing such inputs as well (Han et al. (2022)). Moreover, equivariant graph representation learning algorithms have recently become a de facto methodology for processing scientific data of many shapes and origins Musaelian et al. (2022); Batzner et al. (2022).

### Contributions.

In this work, we connect ideas at the forefront of GDL and generative modeling to advance the state-of-the-art (SOTA) for 3D molecule generation. In detail, we provide the following contributions.

- We introduce the Geometry-Complete Diffusion Model (GCDM) which establishes new SOTA results for unconditional 3D molecule generation.
- We investigate the impact of geometric message-passing on the behavior and performance of DDPMs trained to generate 3D molecular data.
- Our experiments demonstrate the importance of incorporating physical inductive biases within DDPM denoising neural networks when training them on data from physical domains.

## 3 Preliminaries

### 3.1 Diffusion Models

Key to understanding our contributions in this work are denoising diffusion probabilistic models. As alluded to previously, once trained, DDPMs can generate new data of arbitrary shapes, sizes, formats, and geometries by learning to reverse a noising process acting on each model input. More precisely, for a given data point **x**, a diffusion process adds noise to **x** for time step *t* = 0, 1, …, *T* to yield **z**_*t*_, a noisy representation of the input **x** at time step *t*. Such a process is defined by a multivariate Gaussian distribution:

(1) $$q\left(z_{t}|x\right)=𝒩\left(z_{t}|\alpha_{t}x_{t},\sigma_{t}^{2}I\right),$$

where $\alpha_{t}\in {\mathbb{R}}^{+}$ regulates how much feature signal is retained and $\sigma_{t}^{2}$ modulates how much feature noise is added to input **x**. Note that we typically model *α* as a function defined with smooth transitions from *α*_0_ = 1 to *α*_*T*_ = 0, where a special case of such a noising process, the variance preserving process (Sohl-Dickstein et al. (2015); Ho et al. (2020)), is defined by $\alpha_{t}=\sqrt{1-\sigma_{t}^{2}}$. To simplify notation, in this work, we define the feature signal-to-noise ratio as $\mathrm{SNR}(t)=\alpha_{t}^{2}/\sigma_{t}^{2}$. Also interesting to note is that this diffusion process is Markovian in nature, indicating that we have transition distributions as follows:

(2) $$q\left(z_{t}|z_{s}\right)=𝒩\left(z_{t}|\alpha_{t|s}z_{s},\sigma_{t|s}^{2}I\right),$$

for all *t* > *s* with *α*_*t*|*s*_ = *α*_*t*_*/α*_*s*_ and $\sigma_{t|s}^{2}=\sigma_{t}^{2}-\alpha_{t|s}^{2}\sigma_{s}^{2}$. In total, then, we can write the noising process as:

(3) $$q\left(z_{0},z_{1},\ldots ,z_{T}|x\right)=q\left(z_{0}|x\right)\prod_{t=1}^{T}q\left(z_{t}|z_{t-1}\right).$$

If we then define ***μ***_*t*→*s*_(**x**, **z**_*t*_) and *σ*_*t*→*s*_ as

$$\mu_{t\to s}\left(x,z_{t}\right)=\frac{\alpha_{t|s}\sigma_{s}^{2}}{\sigma_{t}^{2}}z_{t}+\frac{\alpha_{s}\sigma_{t|s}^{2}}{\sigma_{t}^{2}}x\;\text{and}\;\sigma_{t\to s}=\frac{\sigma_{t|s}\sigma_{s}}{\sigma_{t}},$$

we have that the inverse of the noising process, the *true denoising process*, is given by the posterior of the transitions conditioned on **x**, a process that is also Gaussian:

(4) $$q\left(z_{s}|x,z_{t}\right)=𝒩\left(z_{s}|\mu_{t\to s}\left(x,z_{t}\right),\sigma_{t\to s}I\right).$$

### The Generative Denoising Process.

In diffusion models, we define the generative process according to the *true denoising process*. However, for such a denoising process, we do not know the value of **x** *a priori*, so we typically approximate it as $\hat{x}=\phi \left(z_{t},t\right)$ using a neural network *ϕ*. Doing so then lets us express the generative transition distribution *p*(**z**_*s*_|**z**_*t*_) as $q\left(z_{s}|\hat{x}\left(z_{t},t\right),z_{t}\right)$. As a practical alternative to Eq. 4, we can represent this expression using our approximation for $\hat{x}$:

(5) $$p\left(z_{s}|z_{t}\right)=𝒩\left(z_{s}|\mu_{t\to s}\left(\hat{x},z_{t}\right),\sigma_{t\to s}^{2}I\right).$$

If we choose to define *s* as *s* = *t* − 1, then we can derive the variational lower bound on the log-likelihood of **x** given our generative model as:

(6) $$\log\;p(x)\geq {ℒ}_{0}+{ℒ}_{base\;}+\sum_{t=1}^{T}{ℒ}_{t},$$

where we note that ${ℒ}_{0}=\log\;p\left(x|z_{0}\right)$ models the likelihood of the data given its noisy representation **z**_0_, ${ℒ}_{base}=-\mathrm{KL}\left(q\left(z_{T}|x\right)|p\left(z_{T}\right)\right)$ models the difference between a standard normal distribution and the final latent variable *q*(**z**_*T*_|**x**), and

$${ℒ}_{t}=-\mathrm{KL}\left(q\left(z_{s}|x,z_{t}\right)|p\left(z_{s}|z_{t}\right)\right)\;\text{for}\;t=1,2,\ldots ,T.$$

Note that, in this formation of diffusion models, our neural network *ϕ* directly predicts $\hat{x}$. However, Ho et al. (2020) and others have found optimization of *ϕ* to be made much easier when instead predicting the Gaussian noise added to **x** to create $\hat{x}$. An intuition for how this changes the neural network’s learning dynamics is that, when predicting back the noise added to the model’s input, the network is being trained to more directly differentiate which part of **z**_*t*_ corresponds to the input’s feature signal (i.e., the underlying data point **x**) and which part corresponds to added feature noise. In doing so, if we let **z**_*t*_ = *α*_*t*_**x** + *σ*_*t*_***ϵ***, our neural network can then predict $\hat{\epsilon }=\phi \left(z_{t},t\right)$ such that:

(7) $$\hat{x}=\left(1/\alpha_{t}\right)z_{t}-\left(\sigma_{t}/\alpha_{t}\right)\hat{\epsilon }.$$

Kingma et al. (2021) and others have since shown that, when parametrizing our denoising neural network in this way, the loss term ${ℒ}_{t}$ reduces to:

(8) $${ℒ}_{t}=E_{\epsilon \sim 𝒩(0,I)}\left[\frac{1}{2}(1-\mathrm{SNR}(t-1)/\mathrm{SNR}(t))\|\epsilon -\hat{\epsilon }{\|}^{2}\right]$$

Note that, in practice, the loss term ${ℒ}_{base}$ should be close to zero when using a noising schedule defined such that *α*_*T*_ ≈ 0. Moreover, if and when *α*_0_ ≈ 1 *and* **x** is a discrete value, we will find ${ℒ}_{0}$ to be close to zero as well.

### 3.2 SE(3) Equivariance

In this work, we will consider designing denoising neural networks, here denoted as *f*, that are equivariant to the action of the special Euclidean group (i.e., *SE*(3)). We say that a function *f* is equivariant to the action of a group *G* if, for all *g* ∈ *G*, we have *T*_*g*_(*f*(**x**)) = *f*(*S*_*g*_(**x**)), where *T*_*g*_ and *S*_*g*_ are linear representations associated with the group element *g* (Serre et al. (1977)). Given that we are considering the SE(3) group, which is generated by 3D translations and rotations, we can represent *T*_*g*_ and *S*_*g*_ by a translation **t** and an orthogonal matrix **R** that rotates coordinates. Then we consider *f* to be equivariant to a rotation **R** if transforming its input **x** yields an equivalent transformation of its output, that is, we have **R***f*(**x**) = *f*(**Rx**).

#### Diffusion Models and Equivariant Distributions.

In this work, we desire for the marginal distribution *p*(**x**) of our denoising neural network to be an invariant distribution. We begin by observing that a conditional distribution *p*(*y*|*x*) is equivariant to the action of 3D rotations by meeting the criterion:

(9) p(y∣x)=p(Ry∣Rx)forallorthogonalR.

Moreover, a distribution is invariant to rotation transformations **R** when

(10) p(y)=p(Ry)forallorthogonalR.

As Köhler et al. (2020) and Xu et al. (2022) have collectively demonstrated, we know that if *p*(**z**_*T*_) is invariant and the neural network we use to parametrize *p*(**z**_*t*−1_|**z**_*t*_) is equivariant, we have, as desired, that the marginal distribution *p*(**x**) of our denoising model is an invariant distribution.

#### SE(3)-Equivariant Points and Features.

In the context of our work, we represent a point cloud as a fully-connected 3D graph 𝒢=(𝒱,ℰ) with $X=\left(x_{1},x_{2},\ldots ,x_{N}\right)\in {\mathbb{R}}^{N\times 3}$ as the respective Cartesian coordinates for each node, where N=|𝒱| and E=|ℰ|. Each node is described by scalar features $H\in {\mathbb{R}}^{N\times h}$ and *m* vector-valued features $\chi \in {\mathbb{R}}^{N\times (m\times 3)}$. Likewise, each edge is described by scalar features $E\in {\mathbb{R}}^{E\times e}$ and *x* vector-valued features $\xi \in {\mathbb{R}}^{E\times (x\times 3)}$. Important to note is that the features **H** and **E** are invariant to rotations, reflections, and translations, whereas the features ***χ*** and ***ξ*** are equivariant to rotations and reflections. In particular, we describe a function **Φ** as *SE*(3)-equivariant if it satisfies the following constraint:

#### Definition 3.1.

(SE(3) Equivariance).

Given (**H**′, **E**′, **X**′, ***χ***′, ***ξ***′) = **Φ**(**H**, **E**, **X**, ***χ***, ***ξ***),

we have $\left(H^{\prime },E^{\prime },QX^{\prime^{\text{T}}}+g,Q\chi^{\prime \text{T}},Q\xi^{\prime^{\text{T}}}\right)=\Phi \left(H,E,QX^{\text{T}}+g,Q\chi^{\text{T}},Q\xi^{\text{T}}\right)$, $\forall Q\in SO(3),\;\forall g\in {\mathbb{R}}^{3\times 1}$.

#### Geometry-Complete Perceptron Networks.

GCPNets are a type of geometric Graph Neural Network that satisfies our SE(3) equivariance constraint (3.1). In this setting, with (*h*_*i*_ ∈ **H**, $\chi_{i}\in \chi$, *e*_*ij*_ ∈ **E***, ξ*_*ij*_ ∈ ***ξ***), GCPNet consists of a composition of Geometry-Complete Graph Convolution (**GCPConv**) layers $\left(h_{i}^{l},\chi_{i}^{l}\right),x_{i}^{l}=GCPConv\left[\left(h_{i}^{l-1},\chi_{i}^{l-1}\right),\left(e_{ij}^{0},\xi_{ij}^{0}\right),x_{i}^{l-1},{ℱ}_{ij}\right]$ which are defined as:

(11) $$n_{i}^{l}=\phi^{l}\left(n_{i}^{l-1},{𝒜}_{\forall j\in 𝒩(i)}\Omega_{\omega }^{l}\left(n_{i}^{l-1},n_{j}^{l-1},e_{ij},{ℱ}_{ij}\right)\right),$$

where $n_{i}^{l}=\left(h_{i}^{l},\chi_{i}^{l}\right);e_{ij}=\left(e_{ij}^{0},\xi_{ij}^{0}\right)$; *ϕ* is a trainable function; *l* signifies the representation depth of the network; 𝒜 is a permutation-invariant aggregation function; Ω_*ω*_ represents a message-passing function corresponding to the *ω*-th **GCP** message-passing layer; and ${𝓕}_{ij}^{t}=\left(a_{ij}^{t},b_{ij}^{t},c_{ij}^{t}\right)$, with $a_{ij}^{t}=\frac{x_{i}^{t}-x_{j}^{t}}{\left\|x_{i}^{t}-x_{j}^{t}\right\|}$, $b_{ij}^{t}=\frac{x_{i}^{t}\times x_{j}^{t}}{x_{i}^{t}\times x_{j}^{t}}$ and $c_{ij}^{t}=a_{ij}^{t}\times b_{ij}^{t}$, respectively.

Lastly, if one desires to update the positions of each node in 𝒢, as we do in the context of 3D molecule generation, **GCPConv** provides a simple, SE(3)-equivariant method to do so using a dedicated **GCP** module as follows:

(12) $$\left(h_{p_{i}}^{l},\chi_{p_{i}}^{l}\right)=GCP_{p}^{l}\left(n_{i}^{l},{ℱ}_{ij}\right)$$

(13) $$x_{i}^{l}=x_{i}^{l-1}+\chi_{p_{i}}^{l},\;\text{where}\;\chi_{p_{i}}^{l}\in {\mathbb{R}}^{1\times 3},$$

where $GCP_{.}^{l}\left(\cdot ,\;{ℱ}_{ij}\right)$ is defined as in (Morehead & Cheng (2022)) to provide rotation and translation invariant updates to *h*_*i*_ and rotation equivariant updates to *χ*_*i*_ following centralization of the input point cloud’s coordinates **X** (Du et al. (2022)). The effect of using feature updates to *χ*_*i*_ to update *x*_*i*_ is, after decentralizing **X** following the final **GCPConv** layer, that updates to *x*_*i*_ then become SE(3)-equivariant. As such, all the transformations described above collectively satisfy the required equivariance constraint in Def. 3.1. Important to note is that, (1) GCPNet performs message passing directly using vector-valued features corresponding to nodes and edges instead of performing insufficient approximations of such geometric quantities using only scalar features, and (2) GCPNet incorporates a biophysical inductive bias concerning reflection symmetries (e.g., molecular chirality) into the network architecture by encoding into the network’s updates to scalar and vector-valued features geometric frames that are not reflection equivariant. For a more detailed description of the subcomponents within GCPNet, we refer interested readers to Morehead & Cheng (2022).

## 4 GCDM: A Geometry-Complete Diffusion Model

**Figure 1:**
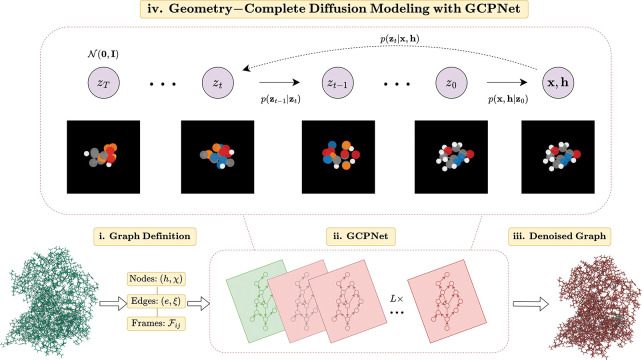
A framework overview for our proposed *Geometry-Complete Diffusion Model* (GCDM). Our framework consists of (**i.**) a graph (topology) definition process, (**ii.**) a GCPNet-based graph neural network for 3D graph representation learning, (**iii.**) denoising of 3D input graphs using GCPNet, and (**iv.**) application of a trained GCPNet denoising network for 3D molecule generation. Zoom in for the best viewing experience.

In this section, we describe GCDM, a new Geometry-Complete SE(3)-Equivariant Diffusion Model, which is illustrated in Figure 1. In particular, we describe how GCDM defines a noising process jointly on equivariant node positions **X** and invariant node features **H** and learns a generative *denoising* process using GCPNet.

### 4.1 The Diffusion Process

In this work, we define an equivariant diffusion process for equivariant node coordinates **x**_*i*_ and invariant node features **h**_*i*_, one that adds random noise to such input data. Recall that the graph inputs to our model, 𝒢, associate with each node a coordinate representation $x_{i}\in {\mathbb{R}}^{3}$ and a feature vector $h\in {\mathbb{R}}^{h}$. Subsequently, let [·, ·] denote the concatenation of two variables. We then define our equivariant noising process on latent variables $z_{t}=\left[z_{t}^{\left(x\right)},z_{t}^{\left(h\right)}\right]$ as:

(14) $$q\left(z_{t}|x,h\right)={𝒩}_{xh}\left(z_{t}|\alpha_{t}[x,h],\sigma_{t}^{2}I\right)\;\text{for}\;t=1,2,\ldots ,T,$$

where we use ${𝒩}_{xh}$ as concise notation to denote the product of two distributions. In this context, the former distribution represents the noised node coordinates ${𝒩}_{x}$, and the latter distribution represents the noised node features ${𝒩}_{h}$. Being as such, ${𝒩}_{xh}$ is given by:

(15) $${𝒩}_{x}\left(z_{t}^{\left(x\right)}|\alpha_{t}x,\sigma_{t}^{2}I\right)\cdot {𝒩}_{h}\left(z_{t}^{\left(h\right)}|\alpha_{t}h,\sigma_{t}^{2}I\right).$$

Note that, with the context of a standard diffusion model in mind, these two equations, Eqs. 14 and 15, correspond to Eq. 1. To address the translation invariance issue raised by Satorras et al. (2021a) in the context of handling a distribution over 3D coordinates, we adopt the zero center of gravity trick proposed by Xu et al. (2022) to define ${𝒩}_{x}$ as a normal distribution on the subspace defined by $\sum_{i}x_{i}=0$. However, to handle node features **h**_*i*_ that are rotation, reflection, and translation-invariant, we can instead use a conventional normal distribution 𝒩.

#### GCDM Generative Denoising Process.

Recall that we need to address the noise posteriors to define a generative process for GCDM. In a similar manner as in Eq. 4, we can directly use the noise posteriors *q*(**z**_*s*_|**x**, **h**, **z**_*t*_) of Eq. 14. To do so, we must replace the input variables **x** and **h** with the approximations $\hat{x}$ and $\hat{h}$ predicted by our denoising neural network:

(16) $$p\left(z_{s}|z_{t}\right)={𝒩}_{xh}\left(z_{s}|\mu_{t\to s}\left([\hat{x},\hat{h}],z_{t}\right),\sigma_{t\to s}^{2}I\right),$$

where our values for $\hat{x}$ and $\hat{h}$ depend on **z**_*t*_, *t*, and our denoising neural network *ϕ*. As mentioned previously, it is often easier to optimize a diffusion model using a noise parametrization to predict the noise $\hat{\epsilon }$ In this work, we use such a parametrization to predict $\hat{\epsilon }=\left[{\hat{\epsilon }}^{\left(x\right)},{\hat{\epsilon }}^{\left(h\right)}\right]$, which represents the noise individually added to $\hat{x}$ and $\hat{h}$. We can then use the predicted $\hat{\epsilon }$ to derive:

(17) $$[\hat{x},\hat{h}]=z_{t}/\alpha_{t}-{\hat{\epsilon }}_{t}\cdot \sigma_{t}/\alpha_{t}.$$

Observe that rotating **z**_*t*_ with **R** yields $R\hat{\epsilon }=\left(\phi \left(Rz_{t},t\right)\right)$. Moreover, since the mean of the denoising distribution (16), one that uses isotropic noise, rotates as $R\hat{x}=Rz_{t}^{\left(x\right)}/\alpha_{t}-R{\hat{\epsilon }}_{t}^{\left(x\right)}\sigma_{t}/\alpha_{t}$, the distribution is equivariant.

Sampling from the model involves sampling $z_{T}\sim 𝒩(0,\;I)$ and then iteratively sampling **z**_*t*_ ~ *p*(**z**_*t*−1_|**z**_*t*_) for *t* = *T, T* − 1, …, 1. Lastly, we can sample **x**, **h** ~ *p*(**x**, **h**|**z**_0_). For a high-level overview of the sampling algorithm for diffusion models such as GCDM, we refer interested readers to Hoogeboom et al. (2022).

#### GCDM Optimization Objective.

When we use the noise parametrization referred to in Eq. 8, the likelihood term of our model, ${ℒ}_{t}=-\mathrm{KL}\left(q\left(z_{s}|x,z_{t}\right)|p\left(z_{s},z_{t}\right)\right)$, notably simplifies. Similar to Hoogeboom et al. (2022), with this parametrization, we observe that ${ℒ}_{t}$ reduces to

(18) $${ℒ}_{t}=E_{\epsilon_{t}\sim {𝒩}_{xh}(0,I)}\left[\frac{1}{2}w(t){\left\|\epsilon_{t}-{\hat{\epsilon }}_{t}\right\|}^{2}\right],$$

where ${\hat{\epsilon }}_{t}=\phi \left(z_{t},t\right)$ and *w*(*t*) = (1 −SNR(*t* − 1)/SNR(*t*)). Following Ho et al. (2020), Hoogeboom et al. (2022), and others, practically speaking, we set *w*(*t*) = 1 to stabilize training and improve sample quality. For a high-level overview of the optimization algorithm for diffusion models such as GCDM, we refer interested readers once again to Hoogeboom et al. (2022).

To summarize, we have defined a diffusion process, a denoising model, and an optimization objective function between them. We now need to define the neural network model *ϕ* that we will reside within the denoising model.

### 4.2 Equivariant Dynamics

We use our previous definition of GCPNet in Section 3.2 to learn an SE(3)-equivariant dynamics function $\left[{\hat{\epsilon }}^{\left(x\right)},{\hat{\epsilon }}^{\left(h\right)}\right]=\phi \left(z_{t}^{\left(x\right)},z_{t}^{\left(h\right)},t\right)$ as:

(19) $${\hat{\epsilon }}_{t}^{\left(x\right)},{\hat{\epsilon }}_{t}^{\left(h\right)}=\mathrm{GCPNet}\left(z_{t}^{\left(x\right)},\left[z_{t}^{\left(h\right)},t/T\right]\right)-\left[z_{t}^{\left(x\right)},0\right],$$

where we inform our denoising model of the current time step by concatenating *t*/*T* as an additional node feature and where we subtract the coordinate representation outputs of GCPNet from its coordinate representation inputs after subtracting from the coordinate representation outputs their collective center of gravity. With the parametrization in Eq. 17, we have subsequently achieved rotation equivariance on ${\hat{x}}_{i}$.

### 4.3 Zeroth Likelihood Terms

For the zeroth likelihood terms corresponding to each type of input feature, we adopt the respective terms previously derived by Hoogeboom et al. (2022). In particular, for integer node features, we adopt the zeroth likelihood term:

(20) $$p\left(h|z_{0}^{\left(h\right)}\right)=\int_{h-\frac{1}{2}}^{h+\frac{1}{2}}𝒩\left(u|z_{0}^{\left(h\right)},\sigma_{0}\right)\text{d}u,$$

where we use the CDF of a standard normal distribution, Φ, to compute Eq. 20 as $\Phi \left(\left(h+\frac{1}{2}-z_{0}^{\left(h\right)}\right)/\sigma_{0}\right)-\Phi \left(\left(h-\frac{1}{2}-z_{0}^{\left(h\right)}\right)/\sigma_{0}\right)\approx 1$ for reasonable noise parameters *α*_0_ and *σ*_0_. For categorical node features, we instead use the zeroth likelihood term:

(21) $$p\left(h|z_{0}^{\left(h\right)}\right)=C(h|p),p\propto \int_{1-\frac{1}{2}}^{1+\frac{1}{2}}𝒩\left(u|z_{0}^{\left(h\right)},\sigma_{0}\right)\text{d}u,$$

where we normalize **p** to sum to one and where *C* is a categorical distribution. Lastly, for continuous node positions, we adopt the zeroth likelihood term:

(22) $$p\left(x|z_{0}^{\left(x\right)}\right)=𝒩\left(x|z_{0}^{\left(x\right)}/\alpha_{0}-\sigma_{0}/\alpha_{0}{\hat{\epsilon }}_{0},\sigma_{0}^{2}/\alpha_{0}^{2}I\right)$$

which gives rise to the log-likelihood component ${ℒ}_{0}^{\left(x\right)}$ as:

(23) $${ℒ}_{0}^{\left(x\right)}=E_{\epsilon^{\left(x\right)}\sim {𝒩}_{x}(0,I)}\left[\log Z^{-1}-\frac{1}{2}{\left\|\epsilon^{x}-\phi^{\left(x\right)}\left(z_{0},0\right)\right\|}^{2}\right],$$

where *d* = 3 and the normalization constant $Z={\left(\sqrt{2\pi }\cdot \sigma_{0}/\alpha_{0}\right)}^{(N-1)\cdot d}$ - in particular, its (*N*−1)·*d* term - arises from the zero center of gravity trick mentioned in Section 4.1.

#### Scaling Node Features.

In line with Hoogeboom et al. (2022), to improve the log-likelihood of our model’s generated samples, we find it useful to train and perform sampling with our model using scaled node feature inputs as $\left[x,\frac{1}{4}h^{\text{(}categorical)},\frac{1}{10}h^{\text{(}integer)}\right]$.

#### Deriving The Number of Atoms.

Finally, to determine the number of atoms with which our model will generate a 3D molecule, we first sample *N* ~ *p*(*N*), where *p*(*N*) denotes the categorical distribution of molecule sizes over the model’s training dataset. Then, we conclude by sampling **x, h** ~ *p*(**x**, **h**|*N*).

## 5 Experiments

### 5.1 Unconditional 3D Molecule Generation - QM9

The QM9 dataset (Ramakrishnan et al. (2014)) contains molecular property descriptions and 3D atom coordinates for 130k small molecules. Each molecule in QM9 can contain up to 9 heavy atoms, that is, 29 atoms when including hydrogens. For the task of 3D molecule generation, we train GCDM to unconditionally generate molecules by producing atom types (H, C, N, O, and F), integer-valued atom charges, and 3D coordinates for each of the molecules’ atoms. Following Anderson et al. (2019), we split QM9 into training, validation, and test partitions consisting of 100k, 18k, and 13k molecule examples, respectively.

#### Metrics.

We adopt the scoring conventions of Satorras et al. (2021a) by using the distance between atom pairs and their respective atom types to predict bond types (single, double, triple, or none). Subsequently, we measure the proportion of generated atoms that have the right valency (atom stability) and the proportion of generated molecules for which all atoms are stable (molecule stability).

#### Baselines.

We compare GCDM to three existing E(3)-equivariant models: G-Schnet (Gebauer et al. (2019)), Equivariant Normalizing Flows (E-NF) (Satorras et al. (2021a)), and Equivariant Diffusion Models (EDM) (Hoogeboom et al. (2022)). For each of these three models, we report their results as reported in Hoogeboom et al. (2022). For comparison with models for this task that are not equivariant, we also report results from Hoogeboom et al. (2022) for Graph Diffusion Models (GDM) trained with random data rotations (GDM-aug) and without them (GDM). To the best of our knowledge, the force-guided molecule generation methods of Wu et al. (2022) are the most recent and performant state-of-the-art methods for 3D molecule generation, so we include their results for this experiment as well.

We further include two GCDM ablation models to more closely analyze the impact of certain key model components within GCDM. These two ablation models include GCDM without local geometric frames ${ℱ}_{ij}$ (i.e., GCDM w/o Frames) and GCDM without scalar message attention (SMA) applied to each edge message (i.e., GCDM w/o SMA) as **m**_*ij*_ = *e*_*ij*_**m**_*ij*_, where **m**_*ij*_ represents the scalar messages learned by GCPNet during geometric graph message passing and *e*_*ij*_ represents a 1 if an edge exists between nodes *i* and *j* (and 0 otherwise) via *e*_*ij*_ ≈ *ϕ*_*inf*_(**m**_*ij*_). Here, $\phi_{inf}:{\mathbb{R}}^{e}\to {\left[0,1\right]}^{1}$ resembles a linear layer followed by a sigmoid function Satorras et al. (2021b). All GCDM models use 9 **GCPConv** layers; SiLU activations (Elfwing et al. (2018)); 256 and 64 scalar node and edge hidden features, respectively; and 32 and 16 vector-valued node and edge features, respectively. All GCDM models are also trained using the AdamW optimizer (Loshchilov & Hutter (2017)) with a batch size of 64, a learning rate of 10^−4^, and a weight decay rate of 10^−12^.

#### Results.

In Table 1, we see that GCDM outperforms previous methods (E-NF, G-Schnet, EDM, Bridge, and Bridge + Force) as well as their non-equivariant counterparts (GDM and GDM-aug) for all metrics, except concerning Atom stable (%) compared to Bridge + Force. We note that the variance of this metric for Bridge + Force is notably larger than that of GCDM. Importantly, when evaluating this metric in conjunction with Mol stable (%), we see that GCDM generates a significantly larger proportion of realistic and stable molecules (negative log-likelihood & Mol stable (%)) compared to all other methods. It is specifically interesting to note how much lower the negative log-likelihood (NLL) of GCDM is compared to that of EDM, the previous NLL-based SOTA method for 3D molecule generation, indicating the generative distribution that GCDM learns from QM9 likely contains much sharper peaks compared to EDM.

To offer additional insights into the behavior of each method for 3D molecule generation, we report as additional metrics the validity of a generated molecule as determined by RDKit (Landrum et al. (2013)) and the uniqueness of the generated molecules overall. Note that for G-Schnet, EDM, Bridge, and Bridge + Force, we directly derive the bonds from the distance between atom pairs. We see in Table 2 that GCDM generates the highest percentage of valid and unique molecules compared to all other methods, improving upon previous SOTA results in such measures by a notable margin. A possible explanation for why GCDM can achieve such results over equivariant methods such as EDM is that GCDM performs geometric (and geometry-complete) message-passing over each 3D input graph to remove the noise present therein, whereas other methods operate solely on scalar features. In future work, it would be interesting to investigate the impact of performing different kinds of geometric message-passing (e.g., type-2 tensor message-passing) on the performance of diffusion models for tasks such as 3D molecule generation.

### 5.2 Conditional 3D Molecule Generation - QM9

#### Baselines.

Towards conditional generation of 3D molecules, we compare GCDM to an existing E(3)-equivariant model, EDM (Hoogeboom et al. (2022)), as well as to two naive baselines: ”Naive (Upper-bound)” where a property classifier *ϕ*_*c*_ predicts molecular properties given a method’s generated 3D molecules and shuffled (i.e., random) property labels; and ”# Atoms” where one uses the numbers of atoms in a method’s generated 3D molecules to predict their molecular properties. For each baseline method, we report its mean absolute error in terms of molecular property prediction by an EGNN classifier *ϕ*_*c*_ Satorras et al. (2021b) as reported in Hoogeboom et al. (2022). For GCDM, we train each conditional model by conditioning it on one of five distinct molecular properties - *α*, gap, homo, lumo, and *μ* - using the QM9 validation split of Hoogeboom et al. (2022) as the model’s training dataset and the QM9 training split of Hoogeboom et al. (2022) as the corresponding EGNN classifier’s training dataset. Consequently, one can expect the gap between a method’s performance and that of ”QM9 (Lower-bound)” to decrease as the method generates molecules that more accurately model a given molecular property.

#### Results.

We see in Table 3 that GCDM outperforms all other methods in conditioning on a given molecular property. In particular, GCDM improves upon the mean absolute error of the SOTA EDM method for the properties *α*, gap, homo, lumo, and *μ* by 28%, 8%, 3%, 18%, and 24%, respectively, demonstrating that, using geometry-complete message passing, GCDM can more accurately model important molecular properties for 3D molecule generation.

## 6 Conclusion

In this work, we introduced GCDM, an SE(3)-equivariant geometry-complete denoising diffusion probabilistic model for 3D molecule generation. While previous equivariant methods for this task have had difficulty establishing sizeable performance gains over non-equivariant methods for this task, GCDM establishes a clear performance advantage over all other methods, generating more realistic, stable, valid, unique, and property-specific 3D molecules compared to existing approaches.

## Figures and Tables

**Table 1:** Comparison of GCPNet with baseline methods for 3D molecule generation. The results are reported in terms of each method’s negative log-likelihood (NLL) - log *p*(**x**, **h**, *N*), atom stability, and molecule stability with standard deviations across three runs on QM9, each drawing 10,000 samples from the model. The top-1 (best) results for this task are in **bold**, and the second-best results are underlined.

Type	Method	NLL ↓	Atoms Stable (%) ↑	Mol Stable (%) ↑

Normalizing Flow	E-NF	−59.7	85.0	4.9

Graph Autoregression	G-Schnet	n/a	95.7	68.1

DDPM	GDM	−94.7	97.0	63.2
	GDM-aug	−92.5	97.6	71.6
	EDM	−110.7 ± 1.5	98.7 ± 0.1	82.0 ± 0.4
	Bridge		98.7 ± 0.1	81.8 ± 0.2
	Bridge + Force		**98.8** ± 0.1	84.6 ± 0.3

DDPM - Ours	GCDM w/o Frames	−162.3 ± 0.3	98.4 ± 0.0	81.7 ± 0.5
	GCDM w/o SMA	−131.3 ± 0.8	95.7 ± 0.1	51.7 ± 1.4
	GCDM	**−171.0** ± 0.2	98.7 ± 0.0	**85.7** ± 0.4

Data			99.0	95.2

**Table 2:** Comparison of GCPNet with baseline methods for 3D molecule generation. The results are reported in terms of the validity and uniqueness of 10,000 samples generated by each method, with standard deviations across three runs on QM9. The best results for this task are in **bold**, and the second-best results are underlined.

Type	Method	Valid (%) ↑	Valid and Unique (%) ↑

Normalizing Flow	E-NF	40.2	39.4

Graph Autoregression	G-Schnet	85.5	80.3

DDPM	GDM-aug	90.4	89.5
	EDM	91.9 ± 0.5	90.7 ± 0.6
	Bridge		90.2
	Bridge + Force		90.7

DDPM - Ours	GCDM w/o Frames	93.9 ± 0.1	92.7 ± 0.1
	GCDM w/o SMA	83.1 ± 1.7	82.8 ± 1.7
	GCDM	**94.8** ± 0.2	**93.3** ± 0.0

Data		97.7	97.7

**Table 3:** Comparison of GCPNet with baseline methods for property-conditional 3D molecule generation. The results are reported in terms of the mean absolute error for molecular property prediction by an EGNN classifier *ϕ_c_* on a QM9 subset, GCDM-generated samples, and two different baselines ”Naive (Upper-bound)” and ”# Atoms”. The top-1 (best) results for this task are in **bold**, and the second-best results are underlined.

Task	*α*	Δ*ϵ*	*ϵ_HOMO_*	*ϵ_LUMO_*	*μ*
Units	*Bohr* ^3^	*meV*	*meV*	*meV*	*D*

Naive (Upper-bound)	9.01	1470	645	1457	1.616
# Atoms	3.86	866	426	813	1.053
EDM	2.76	655	356	584	1.111
GCDM	**1.97**	**602**	**344**	**479**	**0.844**
QM9 (Lower-bound)	0.10	64	39	36	0.043

## References

[R1] Anand Namrata and Achim Tudor. Protein structure and sequence generation with equivariant denoising diffusion probabilistic models. arXiv preprint arXiv:2205.15019, 2022.

[R2] Anderson Brandon, Hy Truong Son, and Kondor Risi. Cormorant: Covariant molecular neural networks. Advances in neural information processing systems, 32, 2019.

[R3] Batzner Simon, Musaelian Albert, Sun Lixin, Geiger Mario, Mailoa Jonathan P, Kornbluth Mordechai, Molinari Nicola, Smidt Tess E, and Kozinsky Boris. E (3)-equivariant graph neural networks for data-efficient and accurate interatomic potentials. Nature communications, 13(1): 2453, 2022. 10.1038/s41467-022-29939-5 PMC9068614 35508450

[R4] Bronstein Michael M, Bruna Joan, Cohen Taco, and Veličković Petar. Geometric deep learning: Grids, groups, graphs, geodesics, and gauges. arXiv preprint arXiv:2104.13478, 2021.

[R5] Brown Tom, Mann Benjamin, Ryder Nick, Subbiah Melanie, Kaplan Jared D, Dhariwal Prafulla, Neelakantan Arvind, Shyam Pranav, Sastry Girish, Askell Amanda, Language models are few-shot learners. Advances in neural information processing systems, 33:1877–1901, 2020.

[R6] Bulusu Srinath, Favoni Matteo, Ipp Andreas, Müller David I, and Schuh Daniel. Generalization capabilities of translationally equivariant neural networks. Physical Review D, 104(7):074504, 2021.

[R7] Cao Wenming, Yan Zhiyue, He Zhiquan, and He Zhihai. A comprehensive survey on geometric deep learning. IEEE Access, 8:35929–35949, 2020.

[R8] Cohen Taco and Welling Max. Group equivariant convolutional networks. In International conference on machine learning, pp. 2990–2999. PMLR, 2016.

[R9] Du Weitao, Zhang He, Du Yuanqi, Meng Qi, Chen Wei, Zheng Nanning, Shao Bin, and Liu Tie-Yan. Se (3) equivariant graph neural networks with complete local frames. In International Conference on Machine Learning, pp. 5583–5608. PMLR, 2022.

[R10] Elfwing Stefan, Uchibe Eiji, and Doya Kenji. Sigmoid-weighted linear units for neural network function approximation in reinforcement learning. Neural Networks, 107:3–11, 2018. 29395652 10.1016/j.neunet.2017.12.012

[R11] Fuchs Fabian, Worrall Daniel, Fischer Volker, and Welling Max. Se (3)-transformers: 3d roto-translation equivariant attention networks. Advances in Neural Information Processing Systems, 33:1970–1981, 2020.

[R12] Gebauer Niklas, Gastegger Michael, and Schütt Kristof. Symmetry-adapted generation of 3d point sets for the targeted discovery of molecules. Advances in neural information processing systems, 32, 2019.

[R13] Han Jiaqi, Rong Yu, Xu Tingyang, and Huang Wenbing. Geometrically equivariant graph neural networks: A survey. arXiv preprint arXiv:2202.07230, 2022.

[R14] Ho Jonathan, Jain Ajay, and Abbeel Pieter. Denoising diffusion probabilistic models. Advances in Neural Information Processing Systems, 33:6840–6851, 2020.

[R15] Hoogeboom Emiel, Satorras Vıctor Garcia, Vignac Clément, and Welling Max. Equivariant diffusion for molecule generation in 3d. In International Conference on Machine Learning, pp. 8867–8887. PMLR, 2022.

[R16] Ingraham John, Baranov Max, Costello Zak, Frappier Vincent, Ismail Ahmed, Tie Shan, Wang Wujie, Xue Vincent, Obermeyer Fritz, Beam Andrew, Illuminating protein space with a programmable generative model. bioRxiv, pp. 2022–12, 2022. 10.1038/s41586-023-06728-8 PMC10686827 37968394

[R17] Joshi Chaitanya K, Bodnar Cristian, Mathis Simon V, Cohen Taco, and Liò Pietro. On the expressive power of geometric graph neural networks. arXiv preprint arXiv:2301.09308, 2023.

[R18] Jumper John, Evans Richard, Pritzel Alexander, Green Tim, Figurnov Michael, Ronneberger Olaf, Tunyasuvunakool Kathryn, Bates Russ, Žídek Augustin, Potapenko Anna, Highly accurate protein structure prediction with alphafold. Nature, 596(7873):583–589, 2021. 34265844 10.1038/s41586-021-03819-2 PMC8371605

[R19] Kingma Diederik, Salimans Tim, Poole Ben, and Ho Jonathan. Variational diffusion models. Advances in neural information processing systems, 34:21696–21707, 2021.

[R20] Kofinas Miltiadis, Nagaraja Naveen, and Gavves Efstratios. Roto-translated local coordinate frames for interacting dynamical systems. Advances in Neural Information Processing Systems, 34:6417–6429, 2021.

[R21] Köhler Jonas, Klein Leon, and Noé Frank. Equivariant flows: exact likelihood generative learning for symmetric densities. In International conference on machine learning, pp. 5361–5370. PMLR, 2020.

[R22] Kong Zhifeng, Ping Wei, Huang Jiaji, Zhao Kexin, and Catanzaro Bryan. Diffwave: A versatile diffusion model for audio synthesis. arXiv preprint arXiv:2009.09761, 2020.

[R23] Landrum Greg Rdkit: A software suite for cheminformatics, computational chemistry, and predictive modeling. Greg Landrum, 8, 2013.

[R24] Yann LeCun Yoshua Bengio, Convolutional networks for images, speech, and time series. The handbook of brain theory and neural networks, 3361(10):1995, 1995.

[R25] Loshchilov Ilya and Hutter Frank. Decoupled weight decay regularization. arXiv preprint arXiv:1711.05101, 2017.

[R26] Luo Shitong, Su Yufeng, Peng Xingang, Wang Sheng, Peng Jian, and Ma Jianzhu. Antigen-specific antibody design and optimization with diffusion-based generative models. bioRxiv, pp. 2022–07, 2022.

[R27] Medsker Larry and Jain Lakhmi C. Recurrent neural networks: design and applications. CRC press, 1999.

[R28] Morehead Alex and Cheng Jianlin. Geometry-complete perceptron networks for 3d molecular graphs. arXiv preprint arXiv:2211.02504, 2022. 10.1093/bioinformatics/btae087 PMC10904142 38373819

[R29] Mudur Nayantara and Finkbeiner Douglas P. Can denoising diffusion probabilistic models generate realistic astrophysical fields? arXiv preprint arXiv:2211.12444, 2022.

[R30] Musaelian Albert, Batzner Simon, Johansson Anders, Sun Lixin, Owen Cameron J, Kornbluth Mordechai, and Kozinsky Boris. Learning local equivariant representations for large-scale atomistic dynamics. arXiv preprint arXiv:2204.05249, 2022. 10.1038/s41467-023-36329-y PMC9898554 36737620

[R31] Peebles William, Radosavovic Ilija, Brooks Tim, Efros Alexei A, and Malik Jitendra. Learning to learn with generative models of neural network checkpoints. arXiv preprint arXiv:2209.12892, 2022.

[R32] Ramakrishnan Raghunathan, Dral Pavlo O, Rupp Matthias, and Von Lilienfeld O Anatole. Quantum chemistry structures and properties of 134 kilo molecules. Scientific data, 1(1):1–7, 2014. 10.1038/sdata.2014.22 PMC4322582 25977779

[R33] Ramesh Aditya, Dhariwal Prafulla, Nichol Alex, Chu Casey, and Chen Mark. Hierarchical text-conditional image generation with clip latents. arXiv preprint arXiv:2204.06125, 2022.

[R34] Rombach Robin, Blattmann Andreas, Lorenz Dominik, Esser Patrick, and Ommer Björn. High-resolution image synthesis with latent diffusion models. In Proceedings of the IEEE/CVF Conference on Computer Vision and Pattern Recognition, pp. 10684–10695, 2022.

[R35] Ruthotto Lars and Haber Eldad. An introduction to deep generative modeling. GAMM-Mitteilungen, 44(2):e202100008, 2021.

[R36] Saharia Chitwan, Chan William, Saxena Saurabh, Li Lala, Whang Jay, Denton Emily, Ghasemipour Seyed Kamyar Seyed, Ayan Burcu Karagol, Mahdavi S Sara, Lopes Rapha Gontijo, Photorealistic text-to-image diffusion models with deep language understanding. arXiv preprint arXiv:2205.11487, 2022.

[R37] Satorras Victor Garcia, Hoogeboom Emiel, Fuchs Fabian B, Posner Ingmar, and Welling Max. E (n) equivariant normalizing flows. arXiv preprint arXiv:2105.09016, 2021a.

[R38] Satorras Vıctor Garcia, Hoogeboom Emiel, and Welling Max. E (n) equivariant graph neural networks. In International conference on machine learning, pp. 9323–9332. PMLR, 2021b.

[R39] Schulman J, Zoph B, Kim C, Hilton J, Menick J, Weng J, Uribe JFC, Fedus L, Metz L, Pokorny M, Chatgpt: Optimizing language models for dialogue, 2022.

[R40] Serre Jean-Pierre Linear representations of finite groups, volume 42. Springer, 1977.

[R41] Sohl-Dickstein Jascha, Weiss Eric, Maheswaranathan Niru, and Ganguli Surya. Deep unsupervised learning using nonequilibrium thermodynamics. In International Conference on Machine Learning, pp. 2256–2265. PMLR, 2015.

[R42] Stärk Hannes, Ganea Octavian, Pattanaik Lagnajit, Barzilay Regina, and Jaakkola Tommi. Equibind: Geometric deep learning for drug binding structure prediction. In International Conference on Machine Learning, pp. 20503–20521. PMLR, 2022.

[R43] Thomas Nathaniel, Smidt Tess, Kearnes Steven, Yang Lusann, Li Li, Kohlhoff Kai, and Riley Patrick. Tensor field networks: Rotation-and translation-equivariant neural networks for 3d point clouds. arXiv preprint arXiv:1802.08219, 2018.

[R44] Vaswani Ashish, Shazeer Noam, Parmar Niki, Uszkoreit Jakob, Jones Llion, Gomez Aidan N, Kaiser Łukasz, and Polosukhin Illia. Attention is all you need. Advances in neural information processing systems, 30, 2017.

[R45] Watson Joseph L, Juergens David, Bennett Nathaniel R, Trippe Brian L, Yim Jason, Eisenach Helen E, Ahern Woody, Borst Andrew J, Ragotte Robert J, Milles Lukas F, Broadly applicable and accurate protein design by integrating structure prediction networks and diffusion generative models. bioRxiv, pp. 2022–12, 2022.

[R46] Wu Lemeng, Gong Chengyue, Liu Xingchao, Ye Mao, and Liu Qiang. Diffusion-based molecule generation with informative prior bridges. arXiv preprint arXiv:2209.00865, 2022.

[R47] Xu Minkai, Yu Lantao, Song Yang, Shi Chence, Ermon Stefano, and Tang Jian. Geodiff: A geometric diffusion model for molecular conformation generation. arXiv preprint arXiv:2203.02923, 2022.

[R48] Zhou Jie, Cui Ganqu, Hu Shengding, Zhang Zhengyan, Yang Cheng, Liu Zhiyuan, Wang Lifeng, Li Changcheng, and Sun Maosong. Graph neural networks: A review of methods and applications. AI open, 1:57–81, 2020.

