# Geometry-Complete Diffusion for 3D Molecule Generation and Optimization

**Published:** 2023-06-17

**Authors:** Alex Morehead, Jianlin Cheng

**Affiliations:** Department of Electrical Engineering & Computer Science, University of Missouri-Columbia, Columbia, MO 65202

## Abstract

Denoising diffusion probabilistic models (DDPMs) have recently taken the field of generative modeling by storm, pioneering new state-of-the-art results in disciplines such as computer vision and computational biology for diverse tasks ranging from text-guided image generation to structure-guided protein design. Along this latter line of research, methods have recently been proposed for generating 3D molecules using equivariant graph neural networks (GNNs) within a DDPM framework. However, such methods are unable to learn important geometric and physical properties of 3D molecules during molecular graph generation, as they adopt molecule-agnostic and non-geometric GNNs as their 3D graph denoising networks, which negatively impacts their ability to effectively scale to datasets of large 3D molecules. In this work, we address these gaps by introducing the Geometry-Complete Diffusion Model (GCDM) for 3D molecule generation, which outperforms existing 3D molecular diffusion models by significant margins across conditional and unconditional settings for the QM9 dataset as well as for the larger GEOM-Drugs dataset. Importantly, we demonstrate that the geometry-complete denoising process GCDM learns for 3D molecule generation allows the model to generate realistic and stable large molecules at the scale of GEOM-Drugs, whereas previous methods fail to do so with the features they learn. Additionally, we show that GCDM’s geometric features can effectively be repurposed to directly optimize the geometry and chemical composition of existing 3D molecules for specific molecular properties, demonstrating new, real-world versatility of molecular diffusion models. Our source code, data, and reproducibility instructions are freely available at https://github.com/BioinfoMachineLearning/bio-diffusion.

## 1 Introduction

Generative modeling has recently been experiencing a renaissance in modeling efforts driven largely by denoising diffusion probabilistic models (DDPMs). At a high level, DDPMs are trained by learning how to denoise a noisy version of an input example. For example, in the context of computer vision, Gaussian noise may be successively added to an input image with the goals of a DDPM in mind. We would then desire for a generative model of images to learn how to successfully distinguish between the original input image’s feature signal and the noise signal added to the image thereafter. If a model can achieve such outcomes, we can use the model to generate novel images by first sampling multivariate Gaussian noise and then iteratively removing, from the current state of the image, the noise predicted by our model. This classic formulation of DDPMs has achieved significant results in the space of image generation (Rombach et al. [2022]), audio synthesis (Kong et al. [2020]), and even meta-learning by learning how to conditionally generate neural network checkpoints (Peebles et al. [2022]). Furthermore, such an approach to generative modeling has expanded its reach to encompass scientific disciplines such as computational biology (Anand and Achim [2022]), computational chemistry (Xu et al. [2022]), and computational physics (Mudur and Finkbeiner [2022]).

Concurrently, the field of geometric deep learning (GDL) (Bronstein et al. [2021]) has seen a sizeable increase in research interest lately, driven largely by theoretical advances within the discipline (Joshi et al. [2023]) as well as by novel applications of such methodology (Stärk et al. [2022]). Notably, such applications even include what is considered by many researchers to be a solution to the problem of predicting 3D protein structures from their corresponding amino acid sequences (Jumper et al. [2021]). Such an outcome arose, in part, from recent advances in sequence-based language modeling efforts (Vaswani et al. [2017], Lin et al. [2023]) as well as from innovations in equivariant neural network modeling (Thomas et al. [2018]).

However, it is currently unclear how the expressiveness of geometric neural networks impacts the ability of generative methods that incorporate them to faithfully model a geometric data distribution. In addition, it is currently unknown whether diffusion models for 3D molecules can be repurposed for important, real-world tasks without retraining or fine-tuning and whether geometric diffusion models are better equipped for such tasks. Toward this end, in this work, we provide the following findings.

- Neural networks that perform message-passing with geometric and geometry-complete quantities enable diffusion generative models of 3D molecules to generate stable and realistic large molecules, whereas non-geometric message-passing networks fail to do so.
- Physical inductive biases such as invariant graph attention and molecular chirality both play important roles in allowing diffusion models to generate valid and realistic 3D molecules.
- Our newly-proposed Geometry-Complete Diffusion Model (GCDM), which incorporates the above insights, establishes new state-of-the-art (SOTA) results for conditional and unconditional 3D molecule generation on the QM9 dataset as well as for unconditional molecule generation on the GEOM-Drugs dataset of large 3D molecules.
- As a first-of-its-kind result, we further demonstrate that geometric diffusion models such as GCDM can effectively perform 3D molecule optimization for specific molecular properties without requiring any retraining or fine-tuning and can do so better than non-geometric diffusion models.

## 2 Related Work

### Generative Modeling.

The field of deep generative modeling (Ruthotto and Haber [2021]) has pioneered a variety of techniques by which to train deep neural networks to create new content similar to that of an existing data repository (e.g., a text dataset of English sentences). Language models such as GPT-3 and ChatGPT (Brown et al. [2020], Schulman et al. [2022]) have become known as hallmark examples of successful generative modeling of text data. In the domains of computer vision and computational biology, techniques such as latent diffusion (Rombach et al. [2022]) and equivariant graph diffusion (Luo et al. [2022]) have established some of the latest state-of-the-art results in generative modeling of images (Tang et al. [2022]) and biomolecules (Hoogeboom et al. [2022], Xu et al. [2023]) such as proteins (Anand and Achim [2022], Yim et al. [2023]), respectively.

### Geometric Deep Learning.

Data residing in a geometric or physical space (e.g., $R^{3}$) can be processed by machine learning algorithms in a plethora of ways. Subsequently, in recent years, the field of geometric deep learning has become known for its proficiency in introducing powerful new deep learning methods designed specifically to process geometric data (Cao et al. [2020]). Examples of popular GDL algorithms include convolutional neural networks designed for working with image data (LeCun et al. [1995]), recurrent neural networks for processing sequence-based data (Medsker and Jain [1999]), and graph neural networks for handling graph-like model inputs (Zhou et al. [2020]).

### Equivariant Neural Networks.

To process geometric data efficiently, however, recent GDL research (Cohen and Welling [2016], Bronstein et al. [2021], Bulusu et al. [2021]) has specifically shown that designing one’s machine learning algorithm to be equivariant to the symmetry groups the input data points naturally respect (e.g., 3D rotation symmetries) often helps such an algorithm generalize to new dataset settings. As a particularly relevant example of a neural network that is equivariant to several important geometric symmetry groups, equivariant graph neural networks (Fuchs et al. [2020], Satorras et al. [2021a], Kofinas et al. [2021]) that are translation and rotation-equivariant to inputs residing in $R^{3}$ have become known as hallmark examples of geometric deep learning algorithms that generalize remarkably well to new inputs and require notably fewer training iterations to converge.

### Representation Learning of Scientific Data.

Scientific data, in particular, requires careful consideration in the context of representation learning. As much scientific data contains within it a notion of geometry or latent structure, equivariance has become a key algorithmic component for processing such inputs as well (Han et al. [2022]). Moreover, equivariant graph representation learning algorithms have recently become a de facto methodology for processing scientific data of many shapes and origins (Musaelian et al. [2022], Batzner et al. [2022]).

## 3 Methods

### 3.1 Problem Setting

In this work, our goal is to generate new 3D molecules either unconditionally or conditioned on user-specified properties. We represent a molecular point cloud as a fully-connected 3D graph 𝒢=(𝒱,ℰ) with 𝒱 and ℰ representing the graph’s set of nodes and set of edges, respectively, and N=|𝒱| and E=|ℰ| representing the number of nodes and the number of edges in the graph, respectively. In addition, $X=\left(x_{1},x_{2},\ldots ,x_{N}\right)\in R^{N\times 3}$ represents the respective Cartesian coordinates for each node (i.e., atom). Each node in 𝒢 is described by scalar features $H\in R^{N\times h}$ and m vector-valued features $\chi \in R^{N\times (m\times 3)}$. Likewise, each edge in 𝒢 is described by scalar features $E\in R^{E\times e}$ and x vector-valued features $\xi \in R^{E\times (x\times 3)}$. Then, let ℳ=[X,H] represent the molecules our method is to generate, where [⋅,⋅] denotes the concatenation of two variables. Important to note is that the input features H and E are invariant to 3D rotations, reflections, and translations, whereas the input features X,χ, and ξ are equivariant to 3D rotations (SO(3)-equivariant) and reflections (O(3)-equivariant). In particular, we say a denoising neural network Φ is 3D rotation and translation-equivariant (i.e., SE(3)-equivariant) if it satisfies the following constraint on its outputs (denoted by ${•}^{\prime }$):

#### Definition 3.1. (SE(3) Equivariance).

Given $\left(H^{\prime },E^{\prime },X^{\prime },\chi^{\prime },\xi^{\prime }\right)=\Phi (H,E,X,\chi ,\xi )$, we have $\left(H^{\prime },E^{\prime },QX^{\prime^{T}}+g,Q\chi^{\prime^{T}},\;Q\xi^{\prime^{T}})=\Phi \left(H,E,QX^{T}+g,Q\chi^{T},Q\xi^{T}\right),\;\forall Q\in SO(3\right)$, $\forall g\in R^{3\times 1}$.

### 3.2 Overview of GCDM

We will now introduce GCDM, a new Geometry-Complete SE(3)-Equivariant Diffusion Model. In particular, we will describe how GCDM defines a joint noising process on equivariant atom coordinates x and invariant atom types h to produce a noisy representation $z=\left[z^{\left(x\right)},z^{\left(h\right)}\right]$ and then learns a generative *denoising* process using our recently-proposed GCPNET (Authors [2023]) model. As we will show in subsequent sections, GCPNET is a desirable architecture for the task of denoising 3D graph inputs in that it contains two distinct feature channels for scalar and vector features, respectively, and supports geometry-complete and chirality-aware message-passing by embedding geometry information-complete local frames for each node (Barron [1986]). Moreover, in our subsequent experiments, we demonstrate that this enables GCPNET to learn more useful equivariant graph representations for generative modeling of 3D molecules.

As an extension of the DDPM framework (Ho et al. [2020]) outlined in Appendix A.1 of our supplementary materials, GCDM is designed to generate molecules in 3D while maintaining SE(3) equivariance, in contrast to previous methods that generate molecules solely in 2D (Jin et al. [2018]) or other dimensionalities (Segler et al. [2018]). GCDM generates molecules by directly placing atoms in continuous 3D space and assigning them discrete types, which is accomplished by modeling forward and reverse diffusion processes, respectively:

(1) $$q\left(z_{1:T}|z_{0}\right)=\prod_{t=1}^{T}q\left(z_{t}|z_{t-1}\right)$$

(2) $$p_{\Phi }\left(z_{0:T-1}|z_{T}\right)=\prod_{t=1}^{T}p_{\Phi }\left(z_{t-1}|z_{t}\right)$$

**Figure 1:**
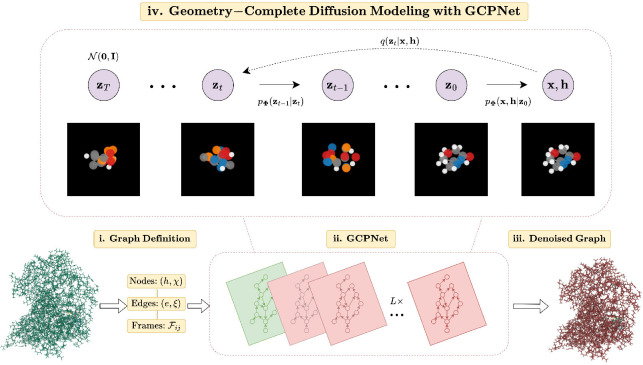
A framework overview for our proposed *Geometry-Complete Diffusion Model* (GCDM). Our framework consists of (**i.**) a graph (topology) definition process, (**ii.**) a GCPNET-based graph neural network for 3D graph representation learning, (**iii.**) denoising of 3D input graphs using GCPNET, and (**iv.**) application of a trained GCPNET denoising network for 3D molecule generation. Zoom in for the best viewing experience.

Overall, these processes describe a latent variable model $p_{\Phi }\left(z_{0}\right)=\int p_{\Phi }\left(z_{0:T}\right)dz_{1:T}$ given a sequence of latent variables $z_{0},z_{1},\ldots ,z_{T}$ matching the dimensionality of the data $ℳ∼p\left(z_{0}\right)$. As illustrated in Figure 1, the forward process (directed from right to left) iteratively adds noise to an input, and the learned reverse process (directed from left to right) iteratively denoises a noisy input to generate new examples from the original data distribution. We will now proceed to formulate GCDM’s joint diffusion process and its remaining practical details.

### 3.3 Joint Molecular Diffusion

Recall that our model’s molecular graph inputs, 𝒢, associate with each node a 3D position $x_{i}\in R^{3}$ and a feature vector $h_{i}\in R^{h}$. By way of adding random noise to these model inputs at each time step t and using a fixed, Markov chain variance schedule $\sigma_{1}^{2},\sigma_{2}^{2},\ldots ,\sigma_{T}^{2}$, we can define a joint molecular diffusion process for equivariant atom coordinates x and invariant atom types h as the product of two distributions (Hoogeboom et al. [2022]):

(3) $$q\left(z_{t}|z_{t-1}\right)={𝒩}_{x}(z_{t}^{\left(x\right)}|\alpha_{t}z_{t-1}^{\left(x\right)},\sigma_{t}^{2}I)\cdot {𝒩}_{h}(z_{t}^{\left(h\right)}|\alpha_{t}z_{t-1}^{\left(h\right)},\sigma_{t}^{2}I).$$

where the first distribution, ${𝒩}_{x}$, represents the noised node coordinates, the second distribution, ${𝒩}_{h}$, represents the noised node features, and $\alpha_{t}=\sqrt{1-\sigma_{t}^{2}}$ following the variance preserving process of Ho et al. [2020]. Using ${𝒩}_{xh}$ as concise notation to denote the product of two normal distributions, we can further simplify Eq. 3 as:

(4) $$q\left(z_{t}|z_{t-1}\right)={𝒩}_{xh}\left(z_{t}|\alpha_{t}z_{t-1},\sigma_{t}^{2}I\right).$$

With $\alpha_{t|s}=\alpha_{t}/\alpha_{s}$ and $\sigma_{t|s}^{2}=\sigma_{t}^{2}-\alpha_{t|s}\sigma_{s}^{2}$ for any t>s, we can directly obtain the noisy data distribution $q\left(z_{t}|z_{0}\right)$ at any time step t:

(5) $$q\left(z_{t}|z_{0}\right)={𝒩}_{xh}(z_{t}|\alpha_{t|0}z_{0},\sigma_{t|0}^{2}I).$$

Bayes Theorem then tells us that if we then define $\mu_{t\to s}\left(z_{t},z_{0}\right)$ and $\sigma_{t\to s}$ as

$$\mu_{t\to s}\left(z_{t},z_{0}\right)=\frac{\alpha_{s}\sigma_{t|s}^{2}}{\sigma_{t}^{2}}z_{0}+\frac{\alpha_{t|s}\sigma_{s}^{2}}{\sigma_{t}^{2}}z_{t}\;\text{and}\;\sigma_{t\to s}=\frac{\sigma_{t|s}\sigma_{s}}{\sigma_{t}},$$

we have that the inverse of the noising process, the *true denoising process*, is given by the posterior of the transitions conditioned on $ℳ∼z_{0}$, a process that is also Gaussian (Hoogeboom et al. [2022]):

(6) $$q\left(z_{s}|z_{t},z_{0}\right)=𝒩\left(z_{s}|\mu_{t\to s}\left(z_{t},z_{0}\right),\sigma_{t\to s}^{2}I\right).$$

### 3.4 Geometry-Complete Parametrization of the Equivariant Reverse Process

#### Noise parametrization.

We now need to define our learned generative reverse process that *denoises* pure noise into realistic examples from the original data distribution. Towards this end, we can directly use the noise posteriors $q\left(z_{s}|z_{t},z_{0}\right)$ of Eq. 4 of our supplementary materials with $z_{0}∼(ℳ=[x,\;h])$. However, to do so, we must replace the input variables x and h with the approximations $\overset{ˆ}{x}$ and $\overset{ˆ}{h}$ predicted by our denoising neural network Φ:

(7) $$p_{\Phi }\left(z_{s}|z_{t}\right)={𝒩}_{xh}\left(z_{s}|\mu_{\Phi_{t\to s}}\left(z_{t},{\tilde{z}}_{0}\right),\sigma_{t\to s}^{2}I\right),$$

where the values for ${\tilde{z}}_{0}=[\overset{ˆ}{x},\overset{ˆ}{h}]$ depend on $z_{t},\;t$, and our denoising neural network Φ.

In the context of diffusion models, many different parametrizations of $\mu_{\Phi_{t\to s}}\left(z_{t},{\tilde{z}}_{0}\right)$ are possible. Prior works have found that it is often easier to optimize a diffusion model using a noise parametrization to predict the noise $\overset{ˆ}{\epsilon }$. In this work, we use such a parametrization to predict $\overset{ˆ}{\epsilon }=[{\overset{ˆ}{\epsilon }}^{\left(x\right)},{\overset{ˆ}{\epsilon }}^{\left(h\right)}]$, which represents the noise individually added to $\overset{ˆ}{x}$ and $\overset{ˆ}{h}$. We can then use the predicted $\overset{ˆ}{\epsilon }$ to derive:

(8) $${\tilde{z}}_{0}=[\overset{ˆ}{x},\overset{ˆ}{h}]=z_{t}/\alpha_{t}-{\overset{ˆ}{\epsilon }}_{t}\cdot \sigma_{t}/\alpha_{t}.$$

#### Invariant likelihood.

Ideally, we desire for a 3D molecular diffusion model to assign the same likelihood to a generated molecule even after arbitrarily rotating or translating it in 3D space. To ensure our model achieves this desirable property for $p_{\Phi }\left(z_{0}\right)$, we can leverage the insight that an invariant distribution composed of an equivariant transition function yields an invariant distribution (Satorras et al. [2021b], Xu et al. [2022], Hoogeboom et al. [2022]). Moreover, to address the translation invariance issue raised by Satorras et al. [2021b] in the context of handling a distribution over 3D coordinates, we adopt the zero center of gravity trick proposed by Xu et al. [2022] to define ${𝒩}_{x}$ as a normal distribution on the subspace defined by $\sum_{i}x_{i}=0$. In contrast, to handle node features $h_{i}$ that are rotation and translation-invariant, we can instead use a conventional normal distribution 𝒩. As such, if we parametrize our transition function $p_{\Phi }$ using an SE(3)-equivariant neural network after using the zero center of gravity trick of Xu et al. [2022], our model will have achieved the desired likelihood invariance property.

#### Geometry-completeness.

Furthermore, in this work, we postulate that certain types of geometric neural networks serve as more effective 3D graph denoising functions for molecular DDPMs. We describe this notion as follows.

#### Hypothesis 3.2. (Geometry-Complete Denoising).

Geometric neural networks that achieve geometry-completeness are more robust in denoising 3D molecular network inputs compared to models that are not geometry-complete, in that geometry-complete methods unambiguously define direction-robust local geometric reference frames.

This hypothesis comes as an extension of the definition of geometry-completeness from Authors [2023]. An intuition for its implications on molecular diffusion models is that geometry-complete networks should be able to more effectively learn the gradients of data distributions (Ho et al. [2020]) in which a global force field is present, as is typically the case with 3D molecules (Du et al. [2022]). This is because, broadly speaking, geometry-complete methods encode local reference frames for each node (or edge) under which the directions of arbitrary global force vectors can be mapped. In addition to describing the theoretical benefits offered to geometry-complete denoising networks, we support this hypothesis through specific ablation studies in Section 4.1.

#### GCPNETS.

Inspired by their recent success in modeling 3D molecular structures with geometry-complete message-passing, as mentioned previously, we will parametrize $p_{\Phi }$ using an extended version of Geometry-Complete Perceptron Networks (GCPNETS) as introduced by Authors [2023]. GCPNET is a geometry-complete graph neural network that is equivariant to SE(3) transformations of its graph inputs and, as such, satisfies our SE(3) equivariance constraint (3.1) and maps nicely to the context of Hypothesis 3.2.

In this setting, with $\left(h_{i}\in H,\;\chi_{i}\in \chi ,\;e_{ij}\in E,\xi_{ij}\in \xi \right)$, GCPNet consists of a composition of Geometry-Complete Graph Convolution (**GCPConv**) layers $\left(h_{i}^{l},\chi_{i}^{l}),\;x_{i}^{l}=GCPConv[(h_{i}^{l-1},\chi_{i}^{l-1}),(e_{ij}^{l-1},\xi_{ij}^{l-1}),x_{i}^{l-1},{ℱ}_{ij}\right]$ which are defined as:

(9) $$n_{i}^{l}=\phi^{l}(n_{i}^{l-1},{𝒜}_{\forall j\in 𝒩(i)}\Omega_{\omega }^{l}(n_{i}^{l-1},n_{j}^{l-1},e_{ij}^{l-1},\xi_{ij}^{l-1},{ℱ}_{ij})),$$

where $n_{i}^{l}=\left(h_{i}^{l},\chi_{i}^{l}\right);\;\phi^{l}$ is a trainable function; l signifies the representation depth of the network; 𝒜 is a permutation-invariant aggregation function; $\Omega_{\omega }$ represents a message-passing function corresponding to the ω-th **GCP** message-passing layer; and node i’s geometry-complete local frames are ${ℱ}_{ij}^{t}=(a_{ij}^{t},b_{ij}^{t},c_{ij}^{t})$ with $a_{ij}^{t}=\frac{x_{i}^{t}-x_{j}^{t}}{∥x_{i}^{t}-x_{j}^{t}∥},\;b_{ij}^{t}=\frac{x_{i}^{t}\times x_{j}^{t}}{∥x_{i}^{t}\times x_{j}^{t}∥}$, and $c_{ij}^{t}=a_{ij}^{t}\times b_{ij}^{t}$, respectively.

Lastly, if one desires to update the coordinate representations of each node in 𝒢, as we do in the context of 3D molecule generation, **GCPConv** provides a simple, SE(3)-equivariant method to do so using a dedicated **GCP** module as follows:

(10) $$\left(h_{p_{i}}^{l},\chi_{p_{i}}^{l}\right)={GCP}_{p}^{l}\left(n_{i}^{l},{ℱ}_{ij}\right)$$

(11) $$x_{i}^{l}=x_{i}^{l-1}+\chi_{p_{i}}^{l},\text{where}\;\chi_{p_{i}}^{l}\in R^{1\times 3},$$

where ${GCP}^{l}\left(\cdot ,{ℱ}_{ij}\right)$ is defined as in (Authors [2023]) to provide chirality-aware rotation and translation-invariant updates to $h_{i}$ and rotation-equivariant updates to $\chi_{i}$ following centralization of the input point cloud’s coordinates X (Du et al. [2022]). The effect of using positional feature updates $\chi_{p_{i}}$ to update $x_{i}$ is, after decentralizing X following the final **GCPConv** layer, that updates to $x_{i}$ then become SE(3)-equivariant. As such, all transformations described above satisfy the required equivariance constraint in Def. 3.1. Therefore, in adapting GCPNET as its 3D graph denoiser, GCDM achieves SE(3) equivariance, geometry-completeness, and likelihood invariance altogether. Note that GCDM subsequently performs message-passing with vector features to denoise its geometric inputs, whereas previous methods denoise their inputs solely using geometrically-insufficient scalar message-passing (Joshi et al. [2023]) as we illustrate through our experiments in our Section 4.

### 3.5 Optimization Objective

Following previous works on diffusion models (Ho et al. [2020], Hoogeboom et al. [2022], Wu et al. [2022]), our noise parametrization chosen for GCDM yields the following model training objective:

(12) $${ℒ}_{t}=E_{\epsilon_{t}∼{𝒩}_{xh}(0,1)}\left[\frac{1}{2}w(t){∥\epsilon_{t}-{\overset{ˆ}{\epsilon }}_{t}∥}^{2}\right],$$

where ${\overset{ˆ}{\epsilon }}_{t}$ is our network’s noise prediction as described above and where we empirically choose to set w(t)=1 for the best possible generation results compared to w(t)=(1-SNR(t-1)/SNR(t)) with $SNR(t)=\alpha_{t}^{2}/\sigma_{t}^{2}$. Additionally, GCDM permits a negative log-likelihood computation using the same optimization terms as Hoogeboom et al. [2022], for which we refer interested readers to Appendices 1.2, 1.3, and 1.4 of our supplementary materials for remaining implementation details.

## 4 Experiments

### 4.1 Unconditional 3D Molecule Generation - QM9

The QM9 dataset (Ramakrishnan et al. [2014]) contains molecular properties and 3D atom coordinates for 130k small molecules. Each molecule in QM9 can contain up to 29 atoms. For the task of 3D molecule generation, we train GCDM to unconditionally generate molecules by producing atom types (H, C, N, O, and F), integer atom charges, and 3D coordinates for each of the molecules’ atoms. Following Anderson et al. [2019], we split QM9 into training, validation, and test partitions consisting of 100k, 18k, and 13k molecule examples, respectively.

#### Metrics.

We adopt the scoring conventions of Satorras et al. [2021b] by using the distance between atom pairs and their respective atom types to predict bond types (single, double, triple, or none) for all but one baseline method (i.e., E-NF). Subsequently, we measure the proportion of generated atoms that have the right valency (atom stability) and the proportion of generated molecules for which all atoms are stable (molecule stability). To offer additional insights into each method’s behavior for 3D molecule generation, we also report the validity of a generated molecule as determined by RDKit (Landrum et al. [2013]) and the uniqueness of the generated molecules overall.

#### Baselines.

Besides including a reference point for molecule quality metrics using QM9 itself (i.e., Data), we compare GCDM (a geometry-complete DDPM - i.e., GC-DDPM) to 10 baseline models for 3D molecule generation using QM9: G-Schnet (Gebauer et al. [2019]); Equivariant Normalizing Flows (E-NF) (Satorras et al. [2021b]); Graph Diffusion Models (GDM) (Hoogeboom et al. [2022]) and their variations (i.e., GCM-aug); Equivariant Diffusion Models (EDM) (Hoogeboom et al. [2022]); Bridge and Bridge + Force (Wu et al. [2022]); latent diffusion models (LDMs) such as GraphLDM and its variation GraphLDM-aug (Xu et al. [2023]); as well as the state-of-the-art GeoLDM method (Xu et al. [2023]). For each of these baseline methods, we report their results as curated by Wu et al. [2022] and Xu et al. [2023]. We further include two GCDM ablation models to more closely analyze the impact of certain key model components within GCDM. These two ablation models include GCDM without chiral and geometry-complete local frames ${ℱ}_{ij}$ (i.e., GCDM w/o Frames) and GCDM without scalar message attention (SMA) applied to each edge message (i.e., GCDM w/o SMA). We refer interested readers to Appendix B.1 of our supplementary materials for further details regarding GCDM’s hyperparameters and optimization with these model configurations.

#### Results.

**Figure 2:**
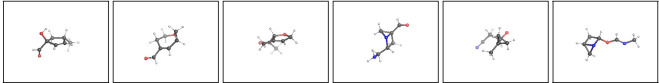
3D molecules generated by GCDM for the QM9 dataset.

In Table 1, we see that GCDM matches or outperforms all previous methods for all metrics, with generated samples shown in Figure 2. In particular, GCDM generates the highest percentage of probable (NLL), valid, and unique molecules compared to all baseline methods, improving upon previous SOTA results in such measures by 54%, 1%, and 1%, respectively. Our ablation of SMA within GCDM demonstrates that, to generate stable 3D molecules, GCDM heavily relies on both being able to perform a lightweight version of fully-connected graph self-attention (Vaswani et al. [2017]), similar to previous methods (Hoogeboom et al. [2022]), which suggests avenues of future research that will be required to scale up such generative models to large biomolecules such as proteins. Additionally, removing geometric local frame embeddings from GCDM reveals that the inductive biases of molecular chirality and geometry-completeness are important contributing factors in GCDM achieving such SOTA results, which provides support for Hypothesis 3.2.

### 4.2 Conditional 3D Molecule Generation - QM9

#### Baselines.

Towards conditional generation of 3D molecules, we compare GCDM to existing E(3)-equivariant models, EDM (Hoogeboom et al. [2022]) and GeoLDM Xu et al. [2023], as well as to two naive baselines: “Naive (Upper-bound)” where a property classifier $\phi_{c}$ predicts molecular properties given a method’s generated 3D molecules and shuffled (i.e., random) property labels; and “# Atoms” where one uses the numbers of atoms in a method’s generated 3D molecules to predict their molecular properties. For each baseline method, we report its mean absolute error in terms of molecular property prediction by an EGNN classifier $\phi_{c}$ (Satorras et al. [2021a]) as reported in Hoogeboom et al. [2022]. For GCDM, we train each conditional model by conditioning it on one of six distinct molecular properties - α, gap, homo, lumo, μ, and $C_{v}$- for approximately 1,500 epochs using the QM9 validation split of Hoogeboom et al. [2022] as the model’s training dataset and the QM9 training split of Hoogeboom et al. [2022] as the corresponding EGNN classifier’s training dataset. Consequently, one can expect the gap between a method’s performance and that of “QM9 (Lower-bound)” to decrease as the method more accurately generates property-specific molecules.

#### Results.

**Figure 3:**
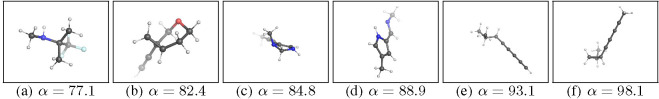
3D molecules generated by GCDM using increasing values of α for the QM9 dataset.

We see in Table 2 that GCDM achieves the best overall results compared to all baseline methods in conditioning on a given molecular property, with conditionally-generated samples shown in Figure 3. In particular, GCDM improves upon the mean absolute error of the SOTA GeoLDM method for four of the six molecular properties - α, lumo, μ, and $C_{v}$ - by 17%, 8%, 24%, and 33%, respectively, and achieves competitive results for the two remaining properties - gap and homo. These results demonstrate that, using geometry-complete diffusion, GCDM can more accurately model important molecular properties for 3D molecule generation. For interested readers, Appendix B.2.1 of our supplementary materials expands upon these conditional modeling results by introducing a novel means of repurposing diffusion generative models for 3D molecule optimization. Such an outcome, where we show GCDM requires only 20 denoising time steps to improve a 3D molecule’s molecular stability by as much as 6%, is to the best of our knowledge the first successful example of its kind for diffusion generative models.

### 4.3 Unconditional 3D Molecule Generation - GEOM-Drugs

The GEOM-Drugs dataset is a well-known source of large, 3D molecular conformers for downstream machine learning tasks. It contains 430k molecules, each with 44 atoms on average and with up to as many as 181 atoms. For this experiment, we collect the 30 lowest-energy conformers corresponding to a molecule and task each baseline method with generating new molecules with 3D positions and types for each constituent atom. Here, we also adopt the negative log-likelihood, atom stability, and molecule stability metrics as defined in Section 4.1 and train GCDM using the same hyperparameters as listed in Appendix B.1 of our supplementary materials, with the exception of training for approximately 75 epochs on GEOM-Drugs.

#### Baselines.

In this experiment, we compare GCDM to several state-of-the-art baseline methods for 3D molecule generation on GEOM-Drugs. Similar to our experiments on QM9, in addition to including a reference point for molecule quality metrics using GEOM-Drugs itself (i.e., Data), here we also compare against E-NF, GDM, GDM-aug, EDM, Bridge along with its variant Bridge + Force, as well as GraphLDM, GraphLDM-aug, and GeoLDM.

#### Results.

**Figure 4:**
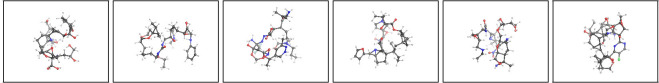
3D molecules generated by GCDM for the GEOM-Drugs dataset.

To start, Table 3 displays an interesting phenomenon: Due to the size of GEOM-Drugs’ molecules and the subsequent errors accumulated when estimating bond types based on inter-atom distances, the baseline results for the molecule stability metrics measured here (i.e., Data) are much lower than those collected for the QM9 dataset. Nonetheless, for GEOM-Drugs, GCDM improves upon SOTA negative log-likelihood results by 71% and upon SOTA atom stability results by 5%, with generated samples shown in Figure 4. Remarkably, to our best knowledge, GCDM is also the first deep learning model that can generate any stable large molecules according to the definitions of atomic and molecular stability in Section 4.1, demonstrating that geometric diffusion models such as GCDM can not only effectively generate large molecules but can also generalize beyond the native distribution of stable molecules within GEOM-Drugs, further empirical support for Hypothesis 3.2.

## 5 Conclusion

While previous methods for 3D molecule generation have possessed insufficient geometric and molecular priors for scaling well to a variety of molecular datasets, in this work, we introduced a geometry-complete diffusion model (GCDM) that establishes a clear performance advantage over previous methods, generating more realistic, stable, valid, unique, and property-specific 3D molecules overall. Moreover, GCDM does so without complex modeling techniques such as latent diffusion, which suggests that GCDM’s results could likely be further improved by expanding upon these techniques (Xu et al. [2023]). Although GCDM’s results here are promising, since it (like previous methods) requires fully-connected graph attention as well as 1,000 time steps to generate a batch of 3D molecules, using it to generate several thousand large molecules can take a notable amount of time (e.g., 15 minutes to generate 100 new large molecules). As such, future research with GCDM could involve adding new time-efficient graph construction or sampling algorithms (Song et al. [2020]) or exploring the impact of higher-order (e.g., type-2 tensor) geometric expressiveness on 3D generative models to accelerate sample generation.

## Figures and Tables

**Table 1:** Comparison of GCPNet with baseline methods for 3D molecule generation. The results are reported in terms of the negative log-likelihood (NLL)-logp(x,h,N), atom stability, molecule stability, validity, and uniqueness of 10,000 samples drawn from each model, with standard deviations for each model across three runs on QM9. The top-1 (best) results for this task are in **bold**, and the second-best results are underlined.

Type	Method	NLL ↓	Atoms Stable (%) ↑	Mol Stable (%) ↑	Valid (%) ↑	Valid and Unique (%) ↑

Normalizing Flow	E-NF	−59.7	85.0	4.9	40.2	39.4

Graph Autoregression	G-Schnet	-	95.7	68.1	85.5	80.3

DDPM	GDM	−94.7	97.0	63.2	-	-
	GDM-aug	−92.5	97.6	71.6	90.4	89.5
	EDM	−110.7 ± 1.5	98.7 ± 0.1	82.0 ± 0.4	91.9 ± 0.5	90.7 ± 0.6
	Bridge	-	98.7 ± 0.1	81.8 ± 0.2	-	90.2
	Bridge + Force	-	98.8 ± 0.1	84.6 ± 0.3	92.0	90.7

LDM	GraphLDM	-	97.2	70.5	83.6	82.7
	GraphLDM-aug	-	97.9	78.7	90.5	89.5
	GeoLDM	-	**98.9** ± 0.1	**89.4** ± 0.5	93.8 ± 0.4	92.7 ± 0.5

GC-DDPM - *Ours*	GCDM w/o Frames	−162.3 ± 0.3	98.4 ± 0.0	81.7 ± 0.5	93.9 ± 0.1	92.7 ± 0.1
	GCDM w/o SMA	−131.3 ± 0.8	95.7 ± 0.1	51.7 ± 1.4	83.1 ± 1.7	82.8 ± 1.7
	GCDM	**−171.0** ± 0.2	98.7 ± 0.0	85.7 ± 0.4	**94.8** ± 0.2	**93.3** ± 0.0

Data			99.0	95.2	97.7	97.7

**Table 2:** Comparison of GCPNET with baseline methods for property-conditional 3D molecule generation. The results are reported in terms of the mean absolute error for molecular property prediction by an EGNN classifier $\phi_{c}$ on a QM9 subset, with results listed for GCDM-generated samples as well as for four separate baseline methods. The top-1 (best) results for this task are in **bold**, and the second-best results are underlined.

Task	*α*	Δ*ϵ*	*ϵ_HOMO_*	*ϵ_LUMO_*	*μ*	*C_v_*
Units	*Bohr* ^3^	*meV*	*meV*	*meV*	*D*	$\frac{cal}{mol}$ *K*

Naive (Upper-bound)	9.01	1470	645	1457	1.616	6.857
# Atoms	3.86	866	426	813	1.053	1.971
EDM	2.76	655	356	584	1.111	1.101
GeoLDM	2.37	**587**	**340**	522	1.108	1.025
GCDM	**1.97**	602	344	**479**	**0.844**	**0.689**

QM9 (Lower-bound)	0.10	64	39	36	0.043	0.040

**Table 3:** Comparison of GCPNET with baseline methods for 3D molecule generation. The results are reported in terms of each method’s negative log-likelihood, atom stability, and molecule stability with standard deviations across three runs on GEOM-Drugs, each drawing 10,000 samples from the model. The top-1 (best) results for this task are in **bold**, and the second-best results are underlined.

Type	Method	NLL ↓	Atoms Stable (%) ↑	Mol Stable (%) ↑

Normalizing Flow	E-NF	-	75.0	0.0

DDPM	GDM	−14.2	75.0	0.0
	GDM-aug	−58.3	77.7	0.0
	EDM	−137.1	81.3	0.0
	Bridge	-	81.0 ± 0.7	0.0
	Bridge + Force	-	82.4 ± 0.8	0.0

LDM	GraphLDM	-	76.2	0.0
	GraphLDM-aug	-	79.6	0.0
	GeoLDM	-	84.4	0.0

GC-DDPM - *Ours*	GCDM w/o Frames	769.7	88.0 ± 0.3	3.4 ± 0.3
	GCDM w/o SMA	3505.5	43.9 ± 3.6	0.1 ± 0.0
	GCDM	**−234.3**	**89.0** ± 0.8	**5.2** ± 1.1

Data			86.5	2.8

## A Appendix - Methods

### A.1 Diffusion Models

Key to understanding our contributions in this work are denoising diffusion probabilistic models (DDPMs). As alluded to previously, once trained, DDPMs can generate new data of arbitrary shapes, sizes, formats, and geometries by learning to reverse a noising process acting on each model input. More precisely, for a given data point x, a diffusion process adds noise to x for time step t=0,1,…,T to yield $z_{t}$, a noisy representation of the input x at time step t. Such a process is defined by a multivariate Gaussian distribution:

(13)
$$q\left(z_{t}|x\right)=𝒩\left(z_{t}|\alpha_{t}x_{t},\sigma_{t}^{2}I\right),$$

where $\alpha_{t}\in R^{+}$ regulates how much feature signal is retained and $\sigma_{t}^{2}$ modulates how much feature noise is added to input x. Note that we typically model α as a function defined with smooth transitions from $\alpha_{0}=1$ to $\alpha_{T}=0$, where a special case of such a noising process, the variance preserving process (Sohl-Dickstein et al. [2015], Ho et al. [2020]), is defined by $\alpha_{t}=\sqrt{1-\sigma_{t}^{2}}$. To simplify notation, in this work, we define the feature signal-to-noise ratio as $SNR(t)=\alpha_{t}^{2}/\sigma_{t}^{2}$. Also interesting to note is that this diffusion process is Markovian in nature, indicating that we have transition distributions as follows:

(14)
$$q\left(z_{t}|z_{s}\right)=𝒩\;(z_{t}|\alpha_{t|s}z_{s},\sigma_{t|s}^{2}I),$$

for all t>s with $\alpha_{t|s}=\alpha_{t}/\alpha_{s}$ and $\sigma_{t|s}^{2}=\sigma_{t}^{2}-\alpha_{t|s}^{2}\sigma_{s}^{2}$. In total, then, we can write the noising process as:

(15)
$$q\left(z_{0},z_{1},\ldots ,z_{T}|x\right)=q\left(z_{0}|x\right)\prod_{t=1}^{T}q\left(z_{t}|z_{t-1}\right).$$

If we then define $\mu_{t\to s}\left(x,z_{t}\right)$ and $\sigma_{t\to s}$ as

$$\mu_{t\to s}\left(x,z_{t}\right)=\frac{\alpha_{t|s}\sigma_{s}^{2}}{\sigma_{t}^{2}}z_{t}+\frac{\alpha_{s}\sigma_{t|s}^{2}}{\sigma_{t}^{2}}x\;\text{and}\;\sigma_{t\to s}=\frac{\sigma_{t|s}\sigma_{s}}{\sigma_{t}},$$

we have that the inverse of the noising process, the *true denoising process*, is given by the posterior of the transitions conditioned on x, a process that is also Gaussian:

(16)
$$q\left(z_{s}|x,z_{t}\right)=𝒩\left(z_{s}|\mu_{t\to s}\left(x,z_{t}\right),\sigma_{t\to s}I\right).$$

#### The Generative Denoising Process.

In diffusion models, we define the generative process according to the true denoising process. However, for such a denoising process, we do not know the value of x a priori, so we typically approximate it as $\overset{ˆ}{x}=\phi \left(z_{t},t\right)$ using a neural network ϕ. Doing so then lets us express the generative transition distribution $p\left(z_{s}|z_{t}\right)$ as $q\left(z_{s}|\overset{ˆ}{x}\left(z_{t},\;t\right),z_{t}\right)$. As a practical alternative to Eq. 16, we can represent this expression using our approximation for $\overset{ˆ}{x}$:

(17)
$$p\left(z_{s}|z_{t}\right)=𝒩\left(z_{s}|\mu_{t\to s}\left(\overset{ˆ}{x},z_{t}\right),\sigma_{t\to s}^{2}I\right).$$

If we choose to define s as s=t-1, then we can derive the variational lower bound on the log-likelihood of x given our generative model as:

(18)
$$\text{log}\;p\left(x\right)\geq {ℒ}_{0}+{ℒ}_{base}+\sum_{t=1}^{T}{ℒ}_{t},$$

where we note that ${ℒ}_{0}=log\;p\left(x|z_{0}\right)$ models the likelihood of the data given its noisy representation $z_{0},\;{ℒ}_{\mathrm{base}}=-KL\left(q\left(z_{T}|x\right)|p\left(z_{T}\right)\right)$ models the difference between a standard normal distribution and the final latent variable $q\left(z_{T}|x\right)$, and

$${ℒ}_{t}=-KL\left(q\left(z_{s}|x,z_{t}\right)|p\left(z_{s}|z_{t}\right)\right)\;\text{for}\;t=1,2,\ldots ,T.$$

Note that, in this formation of diffusion models, our neural network ϕ directly predicts $\overset{ˆ}{x}$. However, Ho et al. [2020] and others have found optimization of ϕ to be made much easier when instead predicting the Gaussian noise added to x to create $\overset{ˆ}{x}$. An intuition for how this changes the neural network’s learning dynamics is that, when predicting back the noise added to the model’s input, the network is being trained to more directly differentiate which part of $z_{t}$ corresponds to the input’s feature signal (i.e., the underlying data point x) and which part corresponds to added feature noise. In doing so, if we let $z_{t}=\alpha_{t}x+\sigma_{t}\epsilon$, our neural network can then predict $\overset{ˆ}{\epsilon }=\phi \left(z_{t},\;t\right)$ such that:

(19)
$$\overset{ˆ}{x}=\left(1/\alpha_{t}\right)z_{t}-\left(\sigma_{t}/\alpha_{t}\right)\overset{ˆ}{\epsilon }.$$

Kingma et al. [2021] and others have since shown that, when parametrizing our denoising neural network in this way, the loss term ${ℒ}_{t}$ reduces to:

(20)
$${ℒ}_{t}=E_{\epsilon ∼𝒩\;(0,I)}\left[\frac{1}{2}(1-SNR(t-1)/SNR(t))∥\epsilon -\overset{ˆ}{\epsilon }{∥}^{2}\right]$$

Note that, in practice, the loss term ${ℒ}_{\mathrm{base}}$ should be close to zero when using a noising schedule defined such that $\alpha_{T}\approx 0$. Moreover, if and when $\alpha_{0}\approx 1$ *and* x is a discrete value, we will find ${ℒ}_{0}$ to be close to zero as well.

### A.2 Zeroth Likelihood Terms for GCDM Optimization Objective

For the zeroth likelihood terms corresponding to each type of input feature, we directly adopt the respective terms previously derived by Hoogeboom et al. [2022]. Doing so enables a negative log-likelihood calculation for GCDM’s predictions. In particular, for integer node features, we adopt the zeroth likelihood term:

(21)
$$p(h|z_{0}^{\left(h\right)})=\int_{h-\frac{1}{2}}^{h+\frac{1}{2}}\;𝒩\;(u|z_{0}^{\left(h\right)},\sigma_{0})du,$$

where we use the CDF of a standard normal distribution, Φ, to compute Eq. 21 as $\Phi ((h+\frac{1}{2}-z_{0}^{\left(h\right)})/\sigma_{0})-\Phi ((h-\frac{1}{2}-z_{0}^{\left(h\right)})/\sigma_{0})\approx 1$ for reasonable noise parameters $\alpha_{0}$ and $\sigma_{0}$ (Hoogeboom et al. [2022]). For categorical node features, we instead use the zeroth likelihood term:

(22)
$$p(h|z_{0}^{\left(h\right)})=C(h|p),p\propto \int_{1-\frac{1}{2}}^{1+\frac{1}{2}}\;𝒩\;(u|z_{0}^{\left(h\right)},\sigma_{0})du,$$

where we normalize p to sum to one and where C is a categorical distribution (Hoogeboom et al. [2022]). Lastly, for continuous node positions, we adopt the zeroth likelihood term:

(23)
$$p(x|z_{0}^{\left(x\right)})=𝒩\left(x|z_{0}^{\left(x\right)}/\alpha_{0}-\sigma_{0}/\alpha_{0}{\overset{ˆ}{\epsilon }}_{0},\sigma_{0}^{2}/\alpha_{0}^{2}I\right)$$

which gives rise to the log-likelihood component ${ℒ}_{0}^{\left(x\right)}$ as:

(24)
$${ℒ}_{0}^{\left(x\right)}=E_{\epsilon^{\left(x\right)}∼{𝒩}_{x}(0,I)}\left[logZ^{-1}-\frac{1}{2}{∥\epsilon^{x}-\phi^{\left(x\right)}\left(z_{0},0\right)∥}^{2}\right],$$

where d=3 and the normalization constant $Z={\left(\sqrt{2\pi }\cdot \sigma_{0}/\alpha_{0}\right)}^{(N-1)\cdot d}$ - in particular, its (N-1)⋅d term - arises from the zero center of gravity trick mentioned in Section 3.4 of the main text (Hoogeboom et al. [2022]).

### A.3 Diffusion Models and Equivariant Distributions

In the context of diffusion generative models of 3D data, one often desires for the marginal distribution p(x) of their denoising neural network to be an invariant distribution. Towards this end, we observe that a conditional distribution p(y|x) is equivariant to the action of 3D rotations by meeting the criterion:

(25)
p(y|x)=p(Ry|Rx)forallorthogonalR.

Moreover, a distribution is invariant to rotation transformations R when

(26)
p(y)=p(Ry)forallorthogonalR.

As Köhler et al. [2020] and Xu et al. [2022] have collectively demonstrated, we know that if $p\left(z_{T}\right)$ is invariant and the neural network we use to parametrize $p\left(z_{t-1}|z_{t}\right)$ is equivariant, we have, as desired, that the marginal distribution p(x) of the denoising model is an invariant distribution.

### A.4 Training and Sampling Procedures for GCDM

#### Equivariant Dynamics.

In this work, we use our previous definition of GCPNET in Section 3.4 of the main text to learn an SE(3)-equivariant dynamics function $\left[{\overset{ˆ}{\epsilon }}^{\left(x\right)},{\overset{ˆ}{\epsilon }}^{\left(h\right)}]=\phi (z_{t}^{\left(x\right)},z_{t}^{\left(h\right)},t\right)$ as:

(27)
$${\overset{ˆ}{\epsilon }}_{t}^{\left(x\right)},{\overset{ˆ}{\epsilon }}_{t}^{\left(h\right)}=GCPNET(z_{t}^{\left(x\right)},[z_{t}^{\left(h\right)},t/T])-[z_{t}^{\left(x\right)},0],$$

where we inform our denoising model of the current time step by concatenating t/T as an additional node feature and where we subtract the coordinate representation outputs of GCPNET from its coordinate representation inputs after subtracting from the coordinate representation outputs their collective center of gravity. With the parametrization in Eq. 8 of the main text, GCDM subsequently achieves rotation equivariance on ${\overset{ˆ}{x}}_{i}$, thereby achieving a 3D translation and rotation-invariant marginal distribution p(x) as described in Appendix A.3.

#### Scaling Node Features.

In line with Hoogeboom et al. [2022], to improve the log-likelihood of our model’s generated samples, we find it useful to train and perform sampling with GCDM using scaled node feature inputs as $\left[x,\;\frac{1}{4}h^{\left(\mathrm{categorical}\right)},\;\frac{1}{10}h^{\left(\mathrm{integer}\right)}\right]$.

#### Deriving The Number of Atoms.

Finally, to determine the number of atoms with which GCDM will generate a 3D molecule, we first sample N∼p(N), where p(N) denotes the categorical distribution of molecule sizes over GCDM’s training dataset. Then, we conclude by sampling x,h∼p(x,h|N).

**Table 4:** Comparison of GCPNET with baseline methods for property-guided 3D molecule optimization. The results are reported in terms of molecular stability *(MS*) and the mean absolute error for molecular property prediction by an EGNN classifier $\phi_{c}$ on a QM9 subset, with results listed for EDM and GCDM-optimized samples as well as two different molecule generation baselines (“EDM Samples” and “GCDM Samples”). The top-1 (best) results for this task are in **bold**, and the second-best results are underlined.

Task	*α / MS*	Δ*ϵ / MS*	*ϵ_HOMO_ / MS*	*ϵ_LUMO_ / MS*	*μ / MS*	*C_v_ / MS*
Units	*Bohr*^3^ / %	*meV* / %	*meV* / %	*meV* / %	*D* / %	$\frac{cal}{mol}$*K* / %

EDM Samples (Moderately Stable)	4.91 / 82.9	1.24 / 82.9	0.55 / 82.9	1.23 / 82.9	1.40 / 82.9	2.84 / 82.9
EDM-Opt (on EDM Samples)	4.80 / 84.4	1.24 / **86.3**	0.55 / 84.4	1.24 / **85.2**	1.41 / 86.0	2.83 / 84.2
GCDM-Opt (on EDM Samples)	**4.76** / **85.2**	**1.22** / 84.0	**0.54** / **84.6**	**1.20** / 83.5	**1.36** / **88.1**	**2.71** / **84.3**

GCDM Samples (Highly Stable)	4.82 / **90.5**	1.19 / 90.5	0.54 / 90.5	1.24 / 90.5	1.32 / 90.5	2.82 / **90.5**
EDM-Opt (on GCDM Samples)	**4.67** / 89.0	1.19 / 90.8	0.54 / 90.8	1.24 / **91.2**	1.32 / **92.6**	**2.80** / 90.0
GCDM-Opt (on GCDM Samples)	4.71 / 90.1	**1.18** / **91.2**	**0.53** / **91.0**	**1.23** / 89.7	**1.30** / 91.3	2.81 / 90.1

## B Appendix - Experiments

### B.1 Additional Technical Details

#### B.1.1 Unconditional 3D Molecule Generation - QM9

In our implementation of scalar message attention (SMA) within GCDM, $m_{ij}=e_{ij}m_{ij}$, where $m_{ij}$ represents the scalar messages learned by GCPNET during message-passing and $e_{ij}$ represents a 1 if an edge exists between nodes i and j (and a 0 otherwise) via $e_{ij}\approx \phi_{inf}\left(m_{ij}\right)$. Here, $\phi_{\inf\;}:R^{e}\to [0,\;1{]}^{1}$ resembles a linear layer followed by a sigmoid function Satorras et al. [2021a].

Moreover, all GCDM models train on QM9 for approximately 1,000 epochs using 9 **GCPConv** layers; SiLU activations (Elfwing et al. [2018]); 256 and 64 scalar node and edge hidden features, respectively; and 32 and 16 vector-valued node and edge features, respectively. All GCDM models are also trained using the AdamW optimizer (Loshchilov and Hutter [2017]) with a batch size of 64, a learning rate of 10^−4^, and a weight decay rate of 10^−12^.

### B.2 Additional Experiments

#### B.2.1 Property-Guided 3D Molecule Optimization - QM9

To evaluate whether molecular diffusion models can not only generate new 3D molecules but can also optimize existing molecules using molecular property guidance, we adopt the QM9 dataset for the following experiment. First, we use an unconditional diffusion model to generate 1,000 3D molecules with each baseline method, and then we provide these molecules to a separate property-conditional diffusion model for optimization of the molecules towards the conditional model’s respective property. This conditional model accepts these 3D molecules as intermediate states for 20 time steps of joint feature denoising, representing 20 time steps of property-guided optimization of the molecules’ atom types and 3D coordinates. Lastly, we repurpose our experimental setup from Section 4.2 of the main text to score these optimized molecules using an external property classifier model to evaluate (1) how much the optimized molecules’ predicted property values have been improved for the respective property (first metric) and (2) whether and how much the optimized molecules’ stability (as defined in Section 4.1 of the main text) has been changed during optimization (second metric).

##### Baselines.

Baseline methods for this experiment include EDM (Hoogeboom et al. [2022]) and GCDM, where both methods use similar experimental setups for evaluation and where each generates 1,000 new molecules for optimization. Our baseline methods also include property-specificity and molecular stability measures of each method’s initial (unconditional) 3D molecules to demonstrate how much molecular diffusion models are able to modify or improve each method’s existing 3D molecules in terms of how property-specific and stable they are. As in Section 4.2 of the main text, property specificity is measured in terms of the corresponding property classifier’s mean absolute error for a given molecule with a targeted property value. Molecular stability (i.e., Mol Stable (%)), here abbreviated at MS, is defined as in Section 4.1 of the main text.

##### Results.

Table 4 showcases an interesting finding: molecular diffusion models for 3D molecule generation can effectively be repurposed as 3D molecular optimization algorithms with minimal modifications, with both baseline optimization methods offering positive refinement results. An interesting observation is that EDM-generated samples (i.e., “EDM Samples”) seem to be easier for each baseline method to optimize in terms of molecular stability due to their initially-lower stability, while GCDM-generated samples (i.e., “GCDM Samples”) appear to be more difficult for methods to refine as a large proportion of these molecules are already quite stable. Moreover, for groups of samples with lower average molecular stability, both baseline diffusion optimization methods seem to primarily improve molecules’ initial stability while also offering small (on average) improvements to their property specificity. In summary, Table 4 shows that GCDM achieves the best optimization results overall in both settings examined, that is, (1) for moderately-stable molecules and (2) for highly-stable molecules. In particular, when optimizing moderately-stable molecules for the molecular property μ, GCDM is simultaneously able to make the initial EDM molecules more property-specific and improve the stability of the molecules by 6% on average, demonstrating that GCDM is capable of not only 3D molecule generation but also 3D molecule optimization (i.e., refinement). Although Table 4 shows that both baseline optimization methods face difficulties in optimizing molecules that are initially highly stable, the results in this setting still show that molecular diffusion models such as GCDM and EDM can achieve success in molecular optimization of highly-stable molecules.

We note that, in general, both baseline methods likely improve the initial molecules’ property specificities only marginally as a function of the small number of optimization steps used. Here, however, we use a small number of optimization steps with both baselines to mimic an important real-world use case of these models: rapid relaxation and optimization of generated molecules at the pace and scale of drug screening procedures in the pharmaceutical industry. To our best knowledge, the results in Table 4 demonstrate the first successful example of using diffusion models to optimize 3D molecules for molecular stability as well as for specific molecular properties, setting the stage for important future applications of these models within modern drug discovery pipelines.

## C Appendix - Additional Details

### C.1 Broader Impacts

In this work, we investigate the impact of geometric representation learning on generative models for 3D molecules. Such research can accelerate the development of new medicinal or energy-related molecular compounds, and, consequently, can contribute to both ethical and unethical use cases of such methodology. However, similar to previous works on molecular generative models (Guan et al. [2023]), we believe the ethical use cases of powerful molecule generation methods far outweigh the unethical use cases (Walters and Murcko [2020]).

### C.2 Training Details

As mentioned in Section B.1, we train each GCDM model using 256 hidden channels for scalar node features, 32 hidden channels for vector-valued node features, 64 hidden channels for scalar edge features, and 16 hidden channels for vector-valued edge features. With a maximum batch size of 64, this 9-layer model configuration allows us to train GCDM models for unconditional (conditional) tasks on the QM9 dataset using approximately 10 (15) days of GPU training time with a single 24GB NVIDIA A10 GPU. For unconditional molecule generation on the much larger GEOM-Drugs dataset, a maximum batch size of 64 allows us to train 4-layer GCDM models using approximately 60 days of GPU training time with a single 48GB NVIDIA RTX A6000 GPU. As such, access to several GPUs with larger GPU memory limits (e.g., 80GBs) should allow one to concurrently train GCDM models in a fraction of the time via larger batch sizes or data-parallel training techniques (Falcon [2019]).

### C.3 Compute Requirements

Training GCDM models for tasks on the QM9 dataset by default requires a GPU with at least 24GB of GPU memory. Inference with such GCDM models for QM9 is much more flexible in terms of GPU memory requirements, as users can directly control how soon a molecule generation batch will complete according to the size of molecules being generated as well as one’s selected batch size during sampling. Training GCDM models for unconditional molecule generation on the GEOM-Drugs dataset by default requires a GPU with at least 48GB of GPU memory. Similar to our GCDM models for QM9, inference with GEOM-Drugs models is flexible in terms of GPU memory requirements according to one’s choice of sampling hyperparameters. Note that inference for both QM9 models and GEOM-Drugs models can likely be accelerated using techniques such as DDIM sampling (Song et al. [2020]). However, we have not officially validated the quality of generated molecules using such sampling techniques, so we caution users to be aware of this potential risk of degrading molecule sample quality when using such sampling algorithms.

### C.4 Reproducibility

On GitHub, we thoroughly provide all source code, data, and instructions required to train new GCDM models or reproduce our results for each of the four molecule generation tasks we study in this work. Our source code uses PyTorch (Paszke et al. [2019]) and PyTorch Lightning (Falcon [2019]) to facilitate model training; PyTorch Geometric (Fey and Lenssen [2019]) to support sparse tensor operations on geometric graphs; and Hydra (Yadan [2019]) to enable reproducible hyperparameter and experiment management.

## References

[R1] Rombach Robin, Blattmann Andreas, Lorenz Dominik, Esser Patrick, and Ommer Björn. High-resolution image synthesis with latent diffusion models. In Proceedings of the IEEE/CVF Conference on Computer Vision and Pattern Recognition, pages 10684–10695, 2022.

[R2] Kong Zhifeng, Ping Wei, Huang Jiaji, Zhao Kexin, and Catanzaro Bryan. Diffwave: A versatile diffusion model for audio synthesis. arXiv preprint arXiv:2009.09761, 2020.

[R3] Peebles William, Radosavovic Ilija, Brooks Tim, Efros Alexei A, and Malik Jitendra. Learning to learn with generative models of neural network checkpoints. arXiv preprint arXiv:2209.12892, 2022.

[R4] Anand Namrata and Achim Tudor. Protein structure and sequence generation with equivariant denoising diffusion probabilistic models. arXiv preprint arXiv:2205.15019, 2022.

[R5] Xu Minkai, Yu Lantao, Song Yang, Shi Chence, Ermon Stefano, and Tang Jian. Geodiff: A geometric diffusion model for molecular conformation generation. arXiv preprint arXiv:2203.02923, 2022.

[R6] Mudur Nayantara and Finkbeiner Douglas P. Can denoising diffusion probabilistic models generate realistic astrophysical fields? arXiv preprint arXiv:2211.12444, 2022.

[R7] Bronstein Michael M, Bruna Joan, Cohen Taco, and Veličković Petar. Geometric deep learning: Grids, groups, graphs, geodesics, and gauges. arXiv preprint arXiv:2104.13478, 2021.

[R8] Joshi Chaitanya K, Bodnar Cristian, Mathis Simon V, Cohen Taco, and Liò Pietro. On the expressive power of geometric graph neural networks. arXiv preprint arXiv:2301.09308, 2023.

[R9] Stärk Hannes, Ganea Octavian, Pattanaik Lagnajit, Barzilay Regina, and Jaakkola Tommi. Equibind: Geometric deep learning for drug binding structure prediction. In International Conference on Machine Learning, pages 20503–20521. PMLR, 2022.

[R10] Jumper John, Evans Richard, Pritzel Alexander, Green Tim, Figurnov Michael, Ronneberger Olaf, Tunyasuvunakool Kathryn, Bates Russ, Žídek Augustin, Potapenko Anna, Highly accurate protein structure prediction with alphafold. Nature, 596(7873):583–589, 2021. 34265844 10.1038/s41586-021-03819-2 PMC8371605

[R11] Vaswani Ashish, Shazeer Noam, Parmar Niki, Uszkoreit Jakob, Jones Llion, Gomez Aidan N, Kaiser Łukasz, and Polosukhin Illia. Attention is all you need. Advances in neural information processing systems, 30, 2017.

[R12] Lin Zeming, Akin Halil, Rao Roshan, Hie Brian, Zhu Zhongkai, Lu Wenting, Smetanin Nikita, Verkuil Robert, Kabeli Ori, Shmueli Yaniv, Evolutionary-scale prediction of atomic-level protein structure with a language model. Science, 379(6637):1123–1130, 2023. 36927031 10.1126/science.ade2574

[R13] Thomas Nathaniel, Smidt Tess, Kearnes Steven, Yang Lusann, Li Li, Kohlhoff Kai, and Riley Patrick. Tensor field networks: Rotation-and translation-equivariant neural networks for 3d point clouds. arXiv preprint arXiv:1802.08219, 2018.

[R14] Ruthotto Lars and Haber Eldad. An introduction to deep generative modeling. GAMM-Mitteilungen, 44(2):e202100008, 2021.

[R15] Brown Tom, Mann Benjamin, Ryder Nick, Subbiah Melanie, Kaplan Jared D, Dhariwal Prafulla, Neelakantan Arvind, Shyam Pranav, Sastry Girish, Askell Amanda, Language models are few-shot learners. Advances in neural information processing systems, 33:1877–1901, 2020.

[R16] Schulman J, Zoph B, Kim C, Hilton J, Menick J, Weng J, Uribe JFC, Fedus L, Metz L, Pokorny M, Chatgpt: Optimizing language models for dialogue, 2022.

[R17] Luo Shitong, Su Yufeng, Peng Xingang, Wang Sheng, Peng Jian, and Ma Jianzhu. Antigen-specific antibody design and optimization with diffusion-based generative models. bioRxiv, pages 2022–07, 2022.

[R18] Tang Raphael, Pandey Akshat, Jiang Zhiying, Yang Gefei, Kumar Karun, Lin Jimmy, and Ture Ferhan. What the daam: Interpreting stable diffusion using cross attention. arXiv preprint arXiv:2210.04885, 2022.

[R19] Hoogeboom Emiel, Satorras Vıctor Garcia, Vignac Clément, and Welling Max. Equivariant diffusion for molecule generation in 3d. In International Conference on Machine Learning, pages 8867–8887. PMLR, 2022.

[R20] Xu Minkai, Powers Alexander, Dror Ron, Ermon Stefano, and Leskovec Jure. Geometric latent diffusion models for 3d molecule generation. arXiv preprint arXiv:2305.01140, 2023.

[R21] Yim Jason, Trippe Brian L, De Bortoli Valentin, Mathieu Emile, Doucet Arnaud, Barzilay Regina, and Jaakkola Tommi. Se (3) diffusion model with application to protein backbone generation. arXiv preprint arXiv:2302.02277, 2023.

[R22] Cao Wenming, Yan Zhiyue, He Zhiquan, and He Zhihai. A comprehensive survey on geometric deep learning. IEEE Access, 8:35929–35949, 2020.

[R23] LeCun Yann, Bengio Yoshua, Convolutional networks for images, speech, and time series. The handbook of brain theory and neural networks, 3361(10):1995, 1995.

[R24] Medsker Larry and Jain Lakhmi C. Recurrent neural networks: design and applications. CRC press, 1999.

[R25] Zhou Jie, Cui Ganqu, Hu Shengding, Zhang Zhengyan, Yang Cheng, Liu Zhiyuan, Wang Lifeng, Li Changcheng, and Sun Maosong. Graph neural networks: A review of methods and applications. AI open, 1:57–81, 2020.

[R26] Cohen Taco and Welling Max. Group equivariant convolutional networks. In International conference on machine learning, pages 2990–2999. PMLR, 2016.

[R27] Bulusu Srinath, Favoni Matteo, Ipp Andreas, David I Müller, and Daniel Schuh. Generalization capabilities of translationally equivariant neural networks. Physical Review D, 104(7):074504, 2021.

[R28] Fuchs Fabian, Worrall Daniel, Fischer Volker, and Welling Max. Se (3)-transformers: 3d roto-translation equivariant attention networks. Advances in Neural Information Processing Systems, 33:1970–1981, 2020.

[R29] Satorras Vıctor Garcia, Hoogeboom Emiel, and Welling Max. E (n) equivariant graph neural networks. In International conference on machine learning, pages 9323–9332. PMLR, 2021a.

[R30] Kofinas Miltiadis, Nagaraja Naveen, and Gavves Efstratios. Roto-translated local coordinate frames for interacting dynamical systems. Advances in Neural Information Processing Systems, 34: 6417–6429, 2021.

[R31] Han Jiaqi, Rong Yu, Xu Tingyang, and Huang Wenbing. Geometrically equivariant graph neural networks: A survey. arXiv preprint arXiv:2202.07230, 2022.

[R32] Musaelian Albert, Batzner Simon, Johansson Anders, Sun Lixin, Owen Cameron J, Kornbluth Mordechai, and Kozinsky Boris. Learning local equivariant representations for large-scale atomistic dynamics. arXiv preprint arXiv:2204.05249, 2022. 10.1038/s41467-023-36329-y PMC9898554 36737620

[R33] Batzner Simon, Musaelian Albert, Sun Lixin, Geiger Mario, Mailoa Jonathan P, Kornbluth Mordechai, Molinari Nicola, Smidt Tess E, and Kozinsky Boris. E(3)-equivariant graph neural networks for data-efficient and accurate interatomic potentials. Nature communications, 13(1):2453, 2022. 10.1038/s41467-022-29939-5 PMC9068614 35508450

[R34] Authors. Geometry-complete perceptron networks for 3d molecular graphs. DLG-AAAI, 2023. 10.1093/bioinformatics/btae087 PMC10904142 38373819

[R35] Barron LD. Symmetry and molecular chirality. Chemical Society Reviews, 15(2):189–223, 1986.

[R36] Ho Jonathan, Jain Ajay, and Abbeel Pieter. Denoising diffusion probabilistic models. Advances in Neural Information Processing Systems, 33:6840–6851, 2020.

[R37] Jin Wengong, Barzilay Regina, and Jaakkola Tommi. Junction tree variational autoencoder for molecular graph generation. In Jennifer Dy and Andreas Krause, editors, Proceedings of the 35th International Conference on Machine Learning, volume 80 of Proceedings of Machine Learning Research, pages 2323–2332. PMLR, 10–15 Jul 2018. URL https://proceedings.mlr.press/v80/jin18a.html.

[R38] Segler Marwin HS, Kogej Thierry, Tyrchan Christian, and Waller Mark P. Generating focused molecule libraries for drug discovery with recurrent neural networks. ACS central science, 4(1): 120–131, 2018. 29392184 10.1021/acscentsci.7b00512 PMC5785775

[R39] Satorras Victor Garcia, Hoogeboom Emiel, Fuchs Fabian B, Posner Ingmar, and Welling Max. E (n) equivariant normalizing flows. arXiv preprint arXiv:2105.09016, 2021b.

[R40] Du Weitao, Zhang He, Du Yuanqi, Meng Qi, Chen Wei, Zheng Nanning, Shao Bin, and Liu Tie-Yan. Se(3) equivariant graph neural networks with complete local frames. In International Conference on Machine Learning, pages 5583–5608. PMLR, 2022.

[R41] Wu Lemeng, Gong Chengyue, Liu Xingchao, Ye Mao, and Liu Qiang. Diffusion-based molecule generation with informative prior bridges. arXiv preprint arXiv:2209.00865, 2022.

[R42] Ramakrishnan Raghunathan, Dral Pavlo O, Rupp Matthias, and Von Lilienfeld O Anatole. Quantum chemistry structures and properties of 134 kilo molecules. Scientific data, 1(1):1–7, 2014. 10.1038/sdata.2014.22 PMC4322582 25977779

[R43] Anderson Brandon, Hy Truong Son, and Kondor Risi. Cormorant: Covariant molecular neural networks. Advances in neural information processing systems, 32, 2019.

[R44] Landrum Greg Rdkit: A software suite for cheminformatics, computational chemistry, and predictive modeling. Greg Landrum, 8, 2013.

[R45] Gebauer Niklas, Gastegger Michael, and Schütt Kristof. Symmetry-adapted generation of 3d point sets for the targeted discovery of molecules. Advances in neural information processing systems, 32, 2019.

[R46] Song Jiaming, Meng Chenlin, and Ermon Stefano. Denoising diffusion implicit models. arXiv preprint arXiv:2010.02502, 2020.

[R47] Jascha Sohl-Dickstein Eric Weiss, Maheswaranathan Niru, and Ganguli Surya. Deep unsupervised learning using nonequilibrium thermodynamics. In International Conference on Machine Learning, pages 2256–2265. PMLR, 2015.

[R48] Kingma Diederik, Salimans Tim, Poole Ben, and Ho Jonathan. Variational diffusion models. Advances in neural information processing systems, 34:21696–21707, 2021.

[R49] Köhler Jonas, Klein Leon, and Noé Frank. Equivariant flows: exact likelihood generative learning for symmetric densities. In International conference on machine learning, pages 5361–5370. PMLR, 2020.

[R50] Elfwing Stefan, Uchibe Eiji, and Doya Kenji. Sigmoid-weighted linear units for neural network function approximation in reinforcement learning. Neural Networks, 107:3–11, 2018. 29395652 10.1016/j.neunet.2017.12.012

[R51] Loshchilov Ilya and Hutter Frank. Decoupled weight decay regularization. arXiv preprint arXiv:1711.05101, 2017.

[R52] Guan Jiaqi, Wesley Wei Qian Xingang Peng, Su Yufeng, Peng Jian, and Ma Jianzhu. 3d equivariant diffusion for target-aware molecule generation and affinity prediction. In The Eleventh International Conference on Learning Representations, 2023. URL https://openreview.net/forum?id=kJqXEPXMsE0.

[R53] Patrick Walters W and Murcko Mark. Assessing the impact of generative ai on medicinal chemistry. Nature biotechnology, 38(2):143–145, 2020. 10.1038/s41587-020-0418-2 32001834

[R54] Falcon William A. Pytorch lightning. GitHub, 3, 2019.

[R55] Paszke Adam, Gross Sam, Massa Francisco, Lerer Adam, Bradbury James, Chanan Gregory, Killeen Trevor, Lin Zeming, Gimelshein Natalia, Antiga Luca, Pytorch: An imperative style, high-performance deep learning library. Advances in neural information processing systems, 32, 2019.

[R56] Fey Matthias and Lenssen Jan Eric. Fast graph representation learning with pytorch geometric. arXiv preprint arXiv:1903.02428, 2019.

[R57] Yadan Omry. Hydra - a framework for elegantly configuring complex applications. Github, 2019. URL https://github.com/facebookresearch/hydra.

