# Geometry-Complete Diffusion for 3D Molecule Generation and Optimization

**Published:** 2024-02-05

**Authors:** Alex Morehead, Jianlin Cheng

**Affiliations:** 1 Electrical Engineering & Computer Science, University of Missouri, W1024 Lafferre Hall, Columbia, 65211, Missouri, USA.

**Keywords:** Geometric deep learning, Diffusion generative modeling, 3D molecules

## Abstract

**Motivation:**

Denoising diffusion probabilistic models (DDPMs) have recently taken the field of generative modeling by storm, pioneering new state-of-the-art results in disciplines such as computer vision and computational biology for diverse tasks ranging from text-guided image generation to structure-guided protein design. Along this latter line of research, methods have recently been proposed for generating 3D molecules using equivariant graph neural networks (GNNs) within a DDPM framework. However, such methods are unable to learn important geometric and physical properties of 3D molecules during molecular graph generation, as they adopt molecule-agnostic and non-geometric GNNs as their 3D graph denoising networks, which negatively impacts their ability to effectively scale to datasets of large 3D molecules.

**Results:**

In this work, we address these gaps by introducing the Geometry-Complete Diffusion Model (GCDM) for 3D molecule generation, which outperforms existing 3D molecular diffusion models by significant margins across conditional and unconditional settings for the QM9 dataset as well as for the larger GEOM-Drugs dataset. Importantly, we demonstrate that the geometry-complete denoising process GCDM learns for 3D molecule generation allows the model to generate realistic and stable large molecules at the scale of GEOM-Drugs, whereas previous methods fail to do so with the features they learn. Additionally, we show that extensions of GCDM can not only effectively design 3D molecules for specific protein pockets but also that GCDM's geometric features can effectively be repurposed to directly optimize the geometry and chemical composition of existing 3D molecules for specific molecular properties, demonstrating new, real-world versatility of molecular diffusion models.

**Availability:**

Our source code and data are freely available on GitHub.

## 1 Introduction

Generative modeling has recently been experiencing a renaissance in modeling efforts driven largely by denoising diffusion probabilistic models (DDPMs). At a high level, DDPMs are trained by learning how to denoise a noisy version of an input example. For example, in the context of computer vision, Gaussian noise may be successively added to an input image with the goals of a DDPM in mind. We would then desire for a generative model of images to learn how to successfully distinguish between the original input image's feature signal and the noise signal added to the image thereafter. If a model can achieve such outcomes, we can use the model to generate novel images by first sampling multivariate Gaussian noise and then iteratively removing, from the current state of the image, the noise predicted by our model. This classic formulation of DDPMs has achieved significant results in the space of image generation [1], audio synthesis [2], and even meta-learning by learning how to conditionally generate neural network checkpoints [3]. Furthermore, such an approach to generative modeling has expanded its reach to encompass scientific disciplines such as computational biology [4, 5], computational chemistry [6], and computational physics [7].

Concurrently, the field of geometric deep learning (GDL) [8] has seen a sizeable increase in research interest lately, driven largely by theoretical advances within the discipline [9] as well as by novel applications of such methodology [10]. Notably, such applications even include what is considered by many researchers to be a solution to the problem of predicting 3D protein structures from their corresponding amino acid sequences [11]. Such an outcome arose, in part, from recent advances in sequence-based language modeling efforts [12, 13] as well as from innovations in equivariant neural network modeling [14].

However, it is currently unclear how the expressiveness of geometric neural networks impacts the ability of generative methods that incorporate them to faithfully model a geometric data distribution. In addition, it is currently unknown whether diffusion models for 3D molecules can be repurposed for important, real-world tasks without retraining or fine-tuning and whether geometric diffusion models are better equipped for such tasks. Toward this end, in this work, we provide the following findings.

- Neural networks that perform message-passing with geometric and geometry-complete quantities enable diffusion generative models of 3D molecules to generate stable and realistic large molecules, whereas non-geometric message-passing networks fail to do so.
- Physical inductive biases such as invariant graph attention and molecular chirality both play important roles in enabling diffusion models to generate valid and realistic 3D molecules.
- Our newly-proposed Geometry-Complete Diffusion Model (GCDM), which is the first diffusion model to incorporate the above insights and achieve the correct type of equivariance for 3D molecules (i.e., SE(3) equivariance), establishes new state-of-the-art (SOTA) results for conditional and unconditional 3D molecule generation on the QM9 dataset as well as for unconditional molecule generation on the GEOM-Drugs dataset of large 3D molecules, and achieves competitive results for protein pocket-conditioned 3D molecule generation.
- As a first-of-its-kind result, we further demonstrate that geometric diffusion models such as GCDM can effectively perform 3D molecule optimization for specific molecular properties without requiring any retraining or fine-tuning and can do so better than non-geometric diffusion models.

## 2 Results

### 2.1 Unconditional 3D Molecule Generation - QM9

The first dataset used in our experiments, the QM9 dataset (Ramakrishnan et al. [15]), contains molecular properties and 3D atom coordinates for 130k small molecules. Each molecule in QM9 can contain up to 29 atoms. For the task of 3D molecule generation, we train GCDM to unconditionally generate molecules by producing atom types (H, C, N, O, and F), integer atom charges, and 3D coordinates for each of the molecules' atoms. Following Anderson et al. [16], we split QM9 into training, validation, and test partitions consisting of 100k, 18k, and 13k molecule examples, respectively.

#### Metrics.

We adopt the scoring conventions of Satorras et al. [17] by using the distance between atom pairs and their respective atom types to predict bond types (single, double, triple, or none) for all but one baseline method (i.e., E-NF). Subsequently, we measure the proportion of generated atoms that have the right valency (atom stability - AS) and the proportion of generated molecules for which all atoms are stable (molecule stability - MS). To offer additional insights into each method's behavior for 3D molecule generation, we also report the validity (Val) of a generated molecule as determined by RDKit (Landrum et al. [18]) and the uniqueness of the generated molecules overall (Uniq).

#### Baselines.

Besides including a reference point for molecule quality metrics using QM9 itself (i.e., Data), we compare GCDM (a geometry-complete DDPM - i.e., GC-DDPM) to 10 baseline models for 3D molecule generation using QM9: G-Schnet (Gebauer et al. [19]); Equivariant Normalizing Flows (E-NF) (Satorras et al. [17]); Graph Diffusion Models (GDM) (Hoogeboom et al. [20]) and their variations (i.e., GCM-aug); Equivariant Diffusion Models (EDM) (Hoogeboom et al. [20]); Bridge and Bridge + Force (Wu et al. [21]); latent diffusion models (LDMs) such as GraphLDM and its variation GraphLDM-aug (Xu et al. [22]); as well as the state-of-the-art GeoLDM method (Xu et al. [22]). For each of these baseline methods, we report their results as curated by Wu et al. [21] and Xu et al. [22]. We further include two GCDM ablation models to more closely analyze the impact of certain key model components within GCDM. These two ablation models include GCDM without chiral and geometry-complete local frames ${ℱ}_{ij}$ (i.e., GCDM w/o Frames) and GCDM without scalar message attention (SMA) applied to each edge message (i.e., GCDM w/o SMA). In Section 3 as well as Appendices A. 1 and B of our supplementary materials, we further discuss GCDM's design, hyperparameters, and optimization with these model configurations.

#### Results.

In Table 1, we see that GCDM matches or outperforms all previous methods for all metrics, with generated samples shown in Figure 2. In particular, GCDM generates the highest percentage of probable (NLL), valid, and unique molecules compared to all baseline methods, improving upon previous SOTA results in such measures by 54%, 1%, and 1%, respectively. Our ablation of SMA within GCDM demonstrates that, to generate stable 3D molecules, GCDM heavily relies on both being able to perform a lightweight version of fully-connected graph self-attention (Vaswani et al. [12]), similar to previous methods (Hoogeboom et al. [20]), which suggests avenues of future research that will be required to scale up such generative models to large biomolecules such as proteins. Additionally, removing geometric local frame embeddings from GCDM reveals that the inductive biases of molecular chirality and geometry-completeness are important contributing factors in GCDM achieving these SOTA results.

### 2.2 Property-Conditional 3D Molecule Generation - QM9

#### Baselines.

Towards the practical use case of conditional generation of 3D molecules, we compare GCDM to existing E(3)-equivariant models, EDM (Hoogeboom et al. [20]) and GeoLDM Xu et al. [22], as well as to two naive baselines: “Naive (Upper-bound)” where a molecular property classifier $\phi_{c}$ predicts molecular properties given a method's generated 3D molecules and shuffled (i.e., random) property labels; and “# Atoms” where one uses the numbers of atoms in a method's generated 3D molecules to predict their molecular properties. For each baseline method, we report its mean absolute error in terms of molecular property prediction by an EGNN classifier $\phi_{c}$ (Satorras et al. [23]) as reported in Hoogeboom et al. [20]. For GCDM, we train each conditional model by conditioning it on one of six distinct molecular property feature inputs - α, gap, homo, lumo, μ, and $C_{v}$ - for approximately 1,500 epochs using the QM9 validation split of Hoogeboom et al. [20] as the model's training dataset and the QM9 training split of Hoogeboom et al. [20] as the corresponding EGNN classifier's training dataset. Consequently, one can expect the gap between a method's performance and that of “QM9 (Lower-bound)” to decrease as the method more accurately generates property-specific molecules.

#### Results.

We see in Table 2 that GCDM achieves the best overall results compared to all baseline methods in conditioning on a given molecular property, with conditionally-generated samples shown in Figure 3. In particular, GCDM improves upon the mean absolute error of the SOTA GeoLDM method for four of the six molecular properties - α, lumo, μ, and $C_{v}$ - by 17%, 8%, 24%, and 33%, respectively, and achieves competitive results for the two remaining properties - gap and homo. These results demonstrate that, using geometry-complete diffusion, GCDM can more accurately model important molecular properties for 3D molecule generation.

### 2.3 Unconditional 3D Molecule Generation - GEOM-Drugs

The second dataset used in our experiments, the GEOM-Drugs dataset, is a well-known source of large, 3D molecular conformers for downstream machine learning tasks. It contains 430k molecules, each with 44 atoms on average and with up to as many as 181 atoms. For this experiment, we collect the 30 lowest-energy conformers corresponding to a molecule and task each baseline method with generating new molecules with 3D positions and types for each constituent atom. Here, we also adopt the negative log-likelihood, atom stability, and molecule stability metrics as defined in Section 2.1 and train GCDM using the same hyperparameters as listed in Appendix B.2 of our supplementary materials, with the exception of training for approximately 75 epochs on GEOM-Drugs.

#### Baselines.

In this experiment, we compare GCDM to several state-of-the-art baseline methods for 3D molecule generation on GEOM-Drugs. Similar to our experiments on QM9, in addition to including a reference point for molecule quality metrics using GEOM-Drugs itself (i.e., Data), here we also compare against E-NF, GDM, GDM-aug, EDM, Bridge along with its variant Bridge + Force, as well as GraphLDM, GraphLDM-aug, and GeoLDM.

#### Results.

To start, Table 3 displays an interesting phenomenon: Due to the size of GEOM-Drugs' molecules and the subsequent errors accumulated when estimating bond types based on inter-atom distances, the baseline results for the molecule stability metrics measured here (i.e., Data) are much lower than those collected for the QM9 dataset. Nonetheless, for GEOM-Drugs, GCDM improves upon SOTA negative log-likelihood results by 71% and upon SOTA atom stability results by 5%, with generated samples shown in Figure 4. Remarkably, to our best knowledge, GCDM is also the first deep learning model that can generate any stable large molecules according to the definitions of atomic and molecular stability in Section 2.1, demonstrating that geometric diffusion models such as GCDM can not only effectively generate large molecules but can also generalize beyond the native distribution of stable molecules within GEOM-Drugs.

### 2.4 Property-Guided 3D Molecule Optimization - QM9

To evaluate whether molecular diffusion models can not only generate new 3D molecules but can also optimize existing molecules using molecular property guidance, we adopt the QM9 dataset for the following experiment. First, we use an unconditional diffusion model to generate 1,000 3D molecules with each baseline method, and then we provide these molecules to a separate property-conditional diffusion model for optimization of the molecules towards the conditional model's respective property. This conditional model accepts these 3D molecules as intermediate states for 20 time steps of joint feature denoising, representing 20 time steps of property-guided optimization of the molecules' atom types and 3D coordinates. Lastly, we repurpose our experimental setup from Section 2.2 to score these optimized molecules using an external property classifier model to evaluate (1) how much the optimized molecules' predicted property values have been improved for the respective property (first metric) and (2) whether and how much the optimized molecules' stability (as defined in Section 2.1) has been changed during optimization (second metric).

#### Baselines.

Baseline methods for this experiment include EDM [20] and GCDM, where both methods use similar experimental setups for evaluation and where each generates 1,000 new molecules for optimization. Our baseline methods also include property-specificity and molecular stability measures of each method's initial (unconditional) 3D molecules to demonstrate how much molecular diffusion models are able to modify or improve each method's existing 3D molecules in terms of how property-specific and stable they are. As in Section 2.2, property specificity is measured in terms of the corresponding property classifier's mean absolute error for a given molecule with a targeted property value. Molecular stability (i.e., Mol Stable (%)), here abbreviated at MS, is defined as in Section 2.1.

#### Results.

Table 4 showcases an interesting finding: molecular diffusion models for 3D molecule generation can effectively be repurposed as 3D molecular optimization algorithms with minimal modifications, with both baseline optimization methods offering positive refinement results. An interesting observation is that EDM-generated samples (i.e., “EDM Samples”) seem to be easier for each baseline method to optimize in terms of molecular stability due to their initially-lower stability, while GCDM-generated samples (i.e., “GCDM Samples”) appear to be more difficult for methods to refine as a large proportion of these molecules are already quite stable. Moreover, for groups of samples with lower average molecular stability, both baseline diffusion optimization methods seem to primarily improve molecules' initial stability while also offering small (on average) improvements to their property specificity. In summary, Table 4 shows that GCDM achieves the best optimization results overall in both settings examined, that is, (1) for moderately stable (i.e., Mod. Stable) molecules and (2) for highly-stable molecules. In particular, when optimizing moderately stable molecules for the molecular property μ, GCDM is simultaneously able to make the initial EDM molecules more property-specific and improve the stability of the molecules by 6% on average, demonstrating that GCDM is capable of not only 3D molecule generation but also 3D molecule optimization (i.e., refinement). Although Table 4 shows that both baseline optimization methods face difficulties in optimizing molecules that are initially highly stable, the results in this setting still show that molecular diffusion models such as GCDM and EDM can achieve success in molecular optimization of highly-stable molecules.

We note that, in general, both baseline methods likely improve the initial molecules' property specificities only marginally as a function of the small number of optimization steps used. Here, however, we use a small number of optimization steps with both baselines to mimic an important real-world use case of these models: rapid relaxation and optimization of generated molecules at the pace and scale of drug screening procedures in the pharmaceutical industry. To our best knowledge, the results in Table 4 demonstrate the first successful example of directly using diffusion models to optimize 3D molecules for molecular stability as well as for specific molecular properties, setting the stage for important future applications of these models within modern drug discovery pipelines.

### 2.5 Protein-Conditional 3D Molecule Generation

To investigate whether geometry-completeness can enhance the ability of molecular diffusion models to generate 3D models within a given protein pocket (i.e., to perform structure-based drug design (SBDD)), in this experiment, we adopt the standard Binding MOAD (BM) [24] and CrossDocked (CD) [25] datasets for training and evaluation of GCDM-SBDD, our geometry-complete, diffusion generative model based on GCPNET++ that extends the diffusion framework of Schneuing et al. [26] for protein pocket-aware molecule generation. The Binding MOAD dataset consists of 100,000 high-quality protein-ligand complexes for training and 100 proteins for testing, with a 30% sequence identity threshold being used to define this cross-validation split. Similarly, the CrossDocked dataset contains 40,484 high-quality protein-ligand complexes split between training (40,354) and test (130) partitions using proteins' enzyme commission numbers as described by Schneuing et al. [26].

#### Baselines.

Baseline methods for this experiment include DiffSBDD-cond [26] and DiffSBDD-joint [26]. We compare these methods to our proposed geometry-complete protein-aware diffusion model, GCDM-SBDD, using metrics that assess the properties, and thereby the quality, of each method's generated molecules. These molecule-averaged metrics include a method's average Vina score (Vina Score - [27]) as a physics-based estimation of a ligand's binding affinity; average drug likeliness QED (QED - [28]); average synthesizability (SA - [29]); on average how many rules of Lipinski's rule of five are satisfied by a ligand (Lipinski - [30]); and average diversity in mean pairwise Tanimoto distances (Diversity - [31, 32]). Following established conventions for 3D molecule generation [20], the size of each ligand to generate was determined using the ligand size distribution of the respective training dataset. Note that, in this context, “joint” and “cond” configurations represent generating a molecule for a protein target, respectively, with and without also modifying the coordinates of the binding pocket within the protein target. Also note that, similar to our experiments in Sections 2.1 – 2.4, our GCDM-SBDD model uses 9 GCP message-passing layers along with 256 and 64 as well as 32 and 16 invariant node and edge scalar features and equivariant node and edge vector features, respectively.

#### Results.

Table 2.5 shows that, across both of the standard SBDD datasets (i.e., Binding MOAD and CrossDocked), GCDM-SBDD generates more synthesizable (SA) and diverse (Diversity) molecules compared to prior methods, potentially due to its use of the SE(3)-equivariant GCPNet++ algorithm as its denoising network. Moreover, across all other metrics, GCDM-SBDD achieves comparable results in terms of drug-likeness measures (QED and Lipinski) and docking (Vina) scores without any hyperparameter tuning. These results suggest that GCDM, along with GCPNet++, not only works well for de novo 3D molecule generation but also protein target-specific 3D molecule generation, notably expanding the number of real-world application areas of GCDM. Moreover, Figure 5 qualitatively shows that GCDM is capable of generating realistic and diverse 3D molecules for unseen protein target pockets.

## 3 Methods

### 3.1 Problem Setting

In this work, our goal is to generate new 3D molecules either unconditionally or conditioned on user-specified properties. We represent a molecular point cloud as a fully-connected 3D graph 𝒢=(𝒱,ℰ) with 𝒱 and ℰ representing the graph's set of nodes and set of edges, respectively, and N=|𝒱| and E=|ℰ| representing the number of nodes and the number of edges in the graph, respectively. In addition, $X=\left(x_{1},x_{2},\ldots ,x_{N}\right)\in R^{N\times 3}$ represents the respective Cartesian coordinates for each node (i.e., atom). Each node in 𝒢 is described by scalar features $H\in R^{N\times h}$ and m vector-valued features $\chi \in R^{N\times (m\times 3)}$. Likewise, each edge in 𝒢 is described by scalar features $E\in R^{E\times e}$ and x vector-valued features $\xi \in R^{E\times (x\times 3)}$. Then, let ℳ=[X,H] represent the molecules our method is to generate, where [⋅,⋅] denotes the concatenation of two variables. Important to note is that the input features H and E are invariant to 3D rotations, reflections, and translations, whereas the input features X, χ, and ξ are equivariant to 3D rotations (*SO(3)*-equivariant) and reflections (*O(3)*-equivariant). In particular, we say a denoising neural network Φ is 3D rotation and translation-equivariant (i.e., SE(3)-equivariant) if it satisfies the following constraint on its outputs (denoted by •′):

**Definition 3.1.** (*SE*(*3*) *Equivariance*).

Given $\left(H^{\prime },E^{\prime },X^{\prime },\chi^{\prime },\xi^{\prime }\right)=\Phi (H,E,X,\chi ,\xi )$, we have $\left(H^{\prime },E^{\prime },QX^{\prime^{T}}+g,Q\chi^{\prime T},Q\xi^{\prime T}\right)=\Phi \left(H,E,QX^{T}+g,Q\chi^{T},Q\xi^{T}\right)$, $\forall Q\in SO(3),\forall g\in R^{3\times 1}$.

### 3.2 Overview of GCDM

We will now introduce GCDM, a new Geometry-Complete SE(3)-Equivariant Diffusion Model. In particular, we will describe how GCDM defines a joint noising process on equivariant atom coordinates x and invariant atom types h to produce a noisy representation $z=\left[z^{\left(x\right)},z^{\left(h\right)}\right]$ and then learns a generative *denoising* process using our proposed GCPNet++ model. As we will show in subsequent sections, GCPNet++ is a desirable architecture for the task of denoising 3D graph inputs in that it contains two distinct feature channels for scalar and vector features, respectively, and supports geometry-complete and chirality-aware message-passing by embedding geometry information-complete local frames for each node (Barron [33]). Moreover, in our subsequent experiments, we demonstrate that this enables GCPNet++ to learn more useful equivariant graph representations for generative modeling of 3D molecules.

As an extension of the DDPM framework (Ho et al. [34]) outlined in Appendix A.1 of our supplementary materials, GCDM is designed to generate molecules in 3D while maintaining SE(3) equivariance, in contrast to previous methods that generate molecules solely in 1D (Segler et al. [35]), 2D (Jin et al. [36]), or 3D modalities without considering chirality (Xu et al. [6], Hoogeboom et al. [20]). GCDM generates molecules by directly placing atoms in continuous 3D space and assigning them discrete types, which is accomplished by modeling forward and reverse diffusion processes, respectively:

(1) $$q\left(z_{1:T}|z_{0}\right)=\prod_{t=1}^{T}\;q\left(z_{t}|z_{t-1}\right)$$

(2) $$p_{\Phi }\left(z_{0:T-1}|z_{T}\right)=\prod_{t=1}^{T}\;p_{\Phi }\left(z_{t-1}|z_{t}\right)$$

**Fig. 1:**
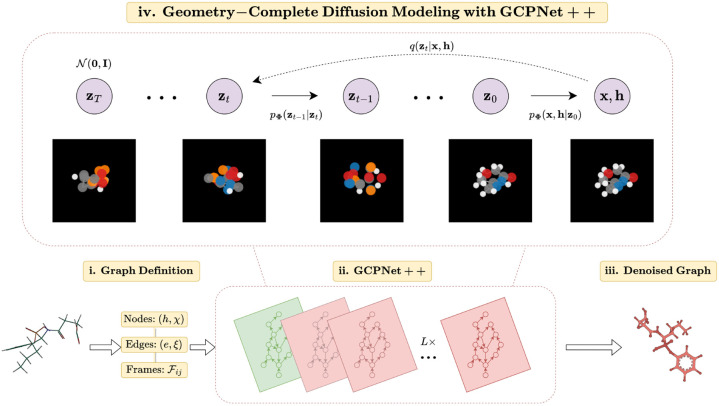
A framework overview for our proposed *Geometry-Complete Diffusion Model* (GCDM). Our framework consists of (**i.**) a graph (topology) definition process, (**ii.**) a GCPNet-based graph neural network for 3D graph representation learning, (**iii.**) denoising of 3D input graphs using GCPNet++, and (**iv.**) application of a trained GCPNet++ denoising network for 3D molecule generation. Zoom in for the best viewing experience.

**Fig. 2:**
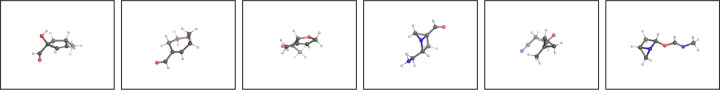
3D molecules generated by GCDM for the QM9 dataset.

**Fig. 3:**
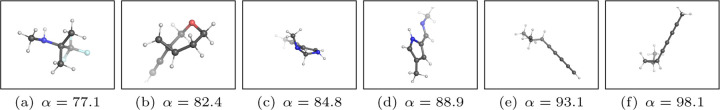
3D molecules generated by GCDM using increasing values of α for the QM9 dataset.

**Fig. 4:**
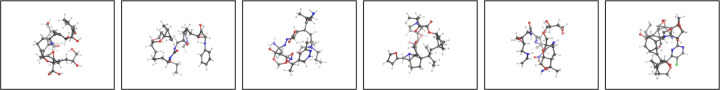
3D molecules generated by GCDM for the GEOM-Drugs dataset.

**Fig. 5:**
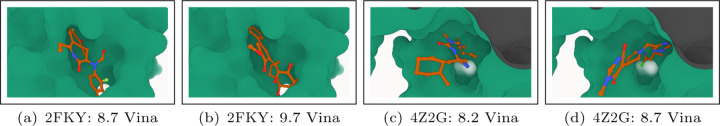
GCDM-SBDD 3D molecules generated for BM (a–b) and CD (c–d) test proteins.

Overall, these processes describe a latent variable model $p_{\Phi }\left(z_{0}\right)=\int p_{\Phi }\left(z_{0:T}\right)dz_{1:T}$ given a sequence of latent variables $z_{0},z_{1},\ldots ,z_{T}$ matching the dimensionality of the data $ℳ\sim p\left(z_{0}\right)$. As illustrated in Figure 1, the forward process (directed from right to left) iteratively adds noise to an input, and the learned reverse process (directed from left to right) iteratively denoises a noisy input to generate new examples from the original data distribution. We will now proceed to formulate GCDM's joint diffusion process and its remaining practical details.

### 3.3 Joint Molecular Diffusion

Recall that our model's molecular graph inputs, 𝒢, associate with each node a 3D position $x_{i}\in R^{3}$ and a feature vector $h_{i}\in R^{h}$. By way of adding random noise to these model inputs at each time step t and using a fixed, Markov chain variance schedule $\sigma_{1}^{2},\sigma_{2}^{2},\ldots ,\sigma_{T}^{2}$, we can define a joint molecular diffusion process for equivariant atom coordinates x and invariant atom types h as the product of two distributions (Hoogeboom et al. [20]):

(3) $$q\left(z_{t}|z_{t-1}\right)={𝒩}_{x}\left(z_{t}^{\left(x\right)}|\alpha_{t}z_{t-1}^{\left(x\right)},\sigma_{t}^{2}I\right)\cdot {𝒩}_{h}\left(z_{t}^{\left(h\right)}|\alpha_{t}z_{t-1}^{\left(h\right)},\sigma_{t}^{2}I\right).$$

where the first distribution, ${𝒩}_{x}$, represents the noised node coordinates, the second distribution, ${𝒩}_{h}$, represents the noised node features, and $\alpha_{t}=\sqrt{1-\sigma_{t}^{2}}$ following the variance preserving process of Ho et al. [34]. Using ${𝒩}_{xh}$ as concise notation to denote the product of two normal distributions, we can further simplify Eq. 3 as:

(4) $$q\left(z_{t}|z_{t-1}\right)={𝒩}_{xh}\left(z_{t}|\alpha_{t}z_{t-1},\sigma_{t}^{2}I\right).$$

With $\alpha_{t|s}=\alpha_{t}/\alpha_{s}$ and $\sigma_{t|s}^{2}=\sigma_{t}^{2}-\alpha_{t|s}\sigma_{s}^{2}$ for any t>s, we can directly obtain the noisy data distribution $q\left(z_{t}|z_{0}\right)$ at any time step t:

(5) $$q\left(z_{t}|z_{0}\right)={𝒩}_{xh}\left(z_{t}|\alpha_{t|0}z_{0},\sigma_{t|0}^{2}I\right).$$

Bayes Theorem then tells us that if we then define $\mu_{t\to s}\left(z_{t},z_{0}\right)$ and $\sigma_{t\to s}$ as

$$\mu_{t\to s}\left(z_{t},z_{0}\right)=\frac{\alpha_{s}\sigma_{t|s}^{2}}{\sigma_{t}^{2}}z_{0}+\frac{\alpha_{t|s}\sigma_{s}^{2}}{\sigma_{t}^{2}}z_{t}\text{ and }\sigma_{t\to s}=\frac{\sigma_{t|s}\sigma_{s}}{\sigma_{t}},$$

we have that the inverse of the noising process, the *true denoising process*, is given by the posterior of the transitions conditioned on $ℳ\sim z_{0}$, a process that is also Gaussian (Hoogeboom et al. [20]):

(6) $$q\left(z_{s}|z_{t},z_{0}\right)=𝒩\left(z_{s}|\mu_{t\to s}\left(z_{t},z_{0}\right),\sigma_{t\to s}^{2}I\right).$$

### 3.4 Geometry-Complete Parametrization of the Equivariant Reverse Process

#### Noise parametrization.

We now need to define our learned generative reverse process that *denoises* pure noise into realistic examples from the original data distribution. Towards this end, we can directly use the noise posteriors $q\left(z_{s}|z_{t},z_{0}\right)$ of Eq. 4 of our supplementary materials with $z_{0}\sim (ℳ=[x,h])$. However, to do so, we must replace the input variables x and h with the approximations $\overset{ˆ}{x}$ and $\overset{ˆ}{h}$ predicted by our denoising neural network Φ:

(7) $$p_{\Phi }\left(z_{s}|z_{t}\right)={𝒩}_{xh}\left(z_{s}|\mu_{\Phi_{t\to s}}\left(z_{t},{\tilde{z}}_{0}\right),\sigma_{t\to s}^{2}I\right),$$

where the values for ${\tilde{z}}_{0}=[\overset{ˆ}{x},\overset{ˆ}{h}]$ depend on $z_{t},t$, and our denoising neural network Φ.

In the context of diffusion models, many different parametrizations of $\mu_{\Phi_{t\to s}}\left(z_{t},{\tilde{z}}_{0}\right)$ are possible. Prior works have found that it is often easier to optimize a diffusion model using a noise parametrization to predict the noise $\overset{ˆ}{\epsilon }$. In this work, we use such a parametrization to predict $\overset{ˆ}{\epsilon }=\left[{\overset{ˆ}{\epsilon }}^{\left(x\right)},{\overset{ˆ}{\epsilon }}^{\left(h\right)}\right]$, which represents the noise individually added to $\overset{ˆ}{x}$ and $\overset{ˆ}{h}$. We can then use the predicted $\overset{ˆ}{\epsilon }$ to derive:

(8) $${\tilde{z}}_{0}=[\overset{ˆ}{x},\overset{ˆ}{h}]=z_{t}/\alpha_{t}-{\overset{ˆ}{\epsilon }}_{t}\cdot \sigma_{t}/\alpha_{t}.$$

#### Invariant likelihood.

Ideally, we desire for a 3D molecular diffusion model to assign the same likelihood to a generated molecule even after arbitrarily rotating or translating it in 3D space. To ensure our model achieves this desirable property for $p_{\Phi }\left(z_{0}\right)$, we can leverage the insight that an invariant distribution composed of an equivariant transition function yields an invariant distribution (Xu et al. [6], Satorras et al. [17], Hoogeboom et al. [20]). Moreover, to address the translation invariance issue raised by Satorras et al. [17] in the context of handling a distribution over 3D coordinates, we adopt the zero center of gravity trick proposed by Xu et al. [6] to define ${𝒩}_{x}$ as a normal distribution on the subspace defined by $\sum_{i}\;x_{i}=0$. In contrast, to handle node features $h_{i}$ that are rotation and translation-invariant, we can instead use a conventional normal distribution 𝒩. As such, if we parametrize our transition function $p_{\Phi }$ using an SE(3)-equivariant neural network after using the zero center of gravity trick of Xu et al. [6], our model will have achieved the desired likelihood invariance property.

#### Geometry-completeness.

Furthermore, in this work, we postulate that certain types of geometric neural networks serve as more effective 3D graph denoising functions for molecular DDPMs. We describe this notion as follows.

**Hypothesis 3.2.** (Geometry-Complete Denoising).

Geometric neural networks that achieve geometry-completeness are more robust in denoising 3D molecular network inputs compared to models that are not geometry-complete, in that geometry-complete methods unambiguously define direction-robust local geometric reference frames.

This hypothesis comes as an extension of the definition of geometry-completeness from Du et al. [37] and Morehead and Cheng [38]:

**Definition 3.3.** (Geometric Completeness).

Given a pair of node positions $\left(x_{i}^{t},x_{j}^{t}\right)$ in a 3D graph 𝒢, with vectors $a_{ij}^{t}\in R^{1\times 3},b_{ij}^{t}\in R^{1\times 3}$, and $c_{ij}^{t}\in R^{1\times 3}$ derived from $\left(x_{i}^{t},x_{j}^{t}\right)$, a local geometric representation $F_{ij}^{t}=\left(a_{ij}^{t},b_{ij}^{t},c_{ij}^{t}\right)\in R^{3\times 3}$ is considered geometrically complete if $F_{ij}^{t}$ is non-degenerate, hence forming a *local orthonormal basis* located at the tangent space of $x_{i}^{t}$.

An intuition for the implications of Hypothesis 3.2 and Definition 3.3 on molecular diffusion models is that geometry-complete networks should be able to more effectively learn the gradients of data distributions (Ho et al. [34]) in which a global force field is present, as is typically the case with 3D molecules (Du et al. [39]). This is because, broadly speaking, geometry-complete methods encode local reference frames for each node (or edge) under which the directions of arbitrary global force vectors can be mapped. In addition to describing the theoretical benefits offered to geometry-complete denoising networks, we support this hypothesis through specific ablation studies in Sections 2.1 and 2.3 where we ablate our geometric frame encodings from GCDM and find that such frames are particularly useful in improving GCDM's ability to generate stable 3D molecules.

#### GCPNet++.

Inspired by its recent success in modeling 3D molecular structures with geometry-complete message-passing, we will parametrize $p_{\Phi }$ using an enhanced version of Geometry-Complete Perceptron Networks (GCPNets) that were originally introduced by Morehead and Cheng [38]. To summarize, GCPNET is a geometry-complete graph neural network that is equivariant to SE(3) transformations of its graph inputs and, as such, satisfies our SE(3) equivariance constraint (3.1) and maps nicely to the context of Hypothesis 3.2.

In this setting, with $\left(h_{i}\in H,\chi_{i}\in \chi ,e_{ij}\in E,\xi_{ij}\in \xi \right)$, GCPNET ++, our enhanced version of GCPNET, consists of a composition of Geometry-Complete Graph Convolution (**GCPConv**) layers $\left(h_{i}^{l},\chi_{i}^{l}\right),x_{i}^{l}=GCPConv\left[\left(h_{i}^{l-1},\chi_{i}^{l-1}\right),\left(e_{ij}^{l-1},\xi_{ij}^{l-1}\right),x_{i}^{l-1},{ℱ}_{ij}\right]$ which are defined as:

(9) $$n_{i}^{l}=\phi^{l}\left(n_{i}^{l-1},{𝒜}_{\forall j\in 𝒩(i)}\Omega_{\omega }^{l}\left(n_{i}^{l-1},n_{j}^{l-1},e_{ij}^{l-1},\xi_{ij}^{l-1},{ℱ}_{ij}\right)\right),$$

where $n_{i}^{l}=\left(h_{i}^{l},\chi_{i}^{l}\right);\phi^{l}$ is a trainable function; l signifies the representation depth of the network; 𝒜 is a permutation-invariant aggregation function; $\Omega_{\omega }$ represents a message-passing function corresponding to the ω-th **GCP** message-passing layer (Morehead and Cheng [38]); and node i 's geometry-complete local frames are ${ℱ}_{ij}^{t}=\left(a_{ij}^{t},b_{ij}^{t},c_{ij}^{t}\right)$, with $a_{ij}^{t}=\frac{x_{i}^{t}-x_{j}^{t}}{∥x_{i}^{t}-x_{j}^{t}∥},b_{ij}^{t}=\frac{x_{i}^{t}\times x_{j}^{t}}{∥x_{i}^{t}\times x_{j}^{t}∥}$, and $c_{ij}^{t}=a_{ij}^{t}\times b_{ij}^{t}$, respectively. Importantly, GCPNet + + restructures the network flow of **GCPConv** (Morehead and Cheng [38]) for each iteration of node feature updates to simplify and enhance information flow, concretely from the form of

(10) $${\overset{ˆ}{n}}^{l}=n^{l-1}+f\left(\Omega_{\omega ,v_{i}}^{l}|v_{i}\in 𝒱\right)$$

to

(11) $${\overset{ˆ}{n}}^{l}=n^{l-1}\cup f\left(\left(g_{e^{\omega },v_{i}}^{l},\Omega_{e^{\omega },v_{i}}^{l},\Omega_{\xi^{\omega },v_{i}}^{l}\right)|v_{i}\in 𝒱\right)$$

and from

(12) $$n^{l}={ResGCP}_{r}^{l}\left({\tilde{n}}_{r-1}^{l}\right)$$

to

(13) $$n^{l}={GCP}_{r}^{l}\left({\tilde{n}}_{r-1}^{l}\right).$$

Note that here f represents a summation or a mean function that is invariant to node order permutations; ∪ denotes the concatenation operation; $g_{e^{\omega },v_{i}}^{l}$ represents the binary-valued (i.e., [0, 1]) output of a scalar message attention (gating) function, expressed as

(14) $$g_{e^{\omega }}^{l}=\sigma_{inf}\left(\phi_{inf}^{l}\left(\Omega_{e^{\omega }}^{l}\right)\right)$$

with $\phi_{\text{inf}}\;:R^{e}\to [0,\;1{]}^{1}$ mapping from high-dimensional scalar edge feature space to a single dimension and σ denoting a sigmoid activation function; r is the node feature update module index; **ResGCP** is a version of the **GCP** module with added residual connections; and $\Omega_{\omega ,v_{i}}^{l}=\left(\Omega_{e^{\omega },v_{i}}^{l},\Omega_{\xi^{\omega },v_{i}}^{l}\right)$ represents the scalar (e) and vector-valued (ξ) messages derived with respect to node $v_{i}$ using up to ω message-passing iterations within each GCPNet++ layer. We found these adaptations to provide state-of-the-art molecule generation results compared to the original node feature updating scheme, which we found yielded sub-optimal results in the context of generative modeling. This highlights the importance of customizing representation learning algorithms for the generative modeling task at hand, since reasonable performance may not always be achievable with them without careful adaptations. It is worth noting that, since GCPNet++ performs message-passing directly on 3D vector features, GCDM is thereby the **first** diffusion generative model that is in principle capable of generating 3D molecules with specific *vector*-valued properties. We leave a full exploration of this idea for future work.

#### Properties of GCDM.

Lastly, if one desires to update the coordinate representations of each node in 𝒢, as we do in the context of 3D molecule generation, the **GCPConv** module of GCPNet++ provides a simple, SE(3)-equivariant method to do so using a dedicated **GCP** module as follows:

(15) $$\left(h_{p_{i}}^{l},\chi_{p_{i}}^{l}\right)={GCP}_{p}^{l}\left(n_{i}^{l},{ℱ}_{ij}\right)$$

(16) $$\begin{array}{rr} & \;\\ x_{i}^{l} & \;=x_{i}^{l-1}+\chi_{p_{i}}^{l},\text{ where }\chi_{p_{i}}^{l}\in R^{1\times 3}, \end{array}$$

where ${GCP}_{.}^{l}\left(\cdot ,{ℱ}_{ij}\right)$ is defined to provide chirality-aware rotation and translationin-variant updates to $h_{i}$ and rotation-equivariant updates to $\chi_{i}$ following centralization of the input point cloud's coordinates X (Du et al. [39]). The effect of using positional feature updates $\chi_{p_{i}}$ to update $x_{i}$ is, after decentralizing X following the final **GCPConv** layer, that updates to $x_{i}$ then become SE(3)-equivariant. As such, all transformations described above satisfy the required equivariance constraint in Def. 3.1. Therefore, in integrating GCPNet++ as its 3D graph denoiser, GCDM achieves SE(3) equivariance, geometry-completeness, and likelihood invariance altogether. Important to note is that GCDM subsequently performs message-passing with vector features to denoise its geometric inputs, whereas previous methods denoise their inputs **solely** using geometrically-insufficient scalar message-passing [9] as we demonstrate through our experiments in our Section 2.

### 3.5 Optimization Objective

Following previous works on diffusion models (Hoogeboom et al. [20], Wu et al. [21], Ho et al. [34]), our noise parametrization chosen for GCDM yields the following model training objective:

(17) $${ℒ}_{t}=E_{\epsilon_{t}\sim {𝒩}_{xh}(0,1)}\left[\frac{1}{2}w(t){∥\epsilon_{t}-{\overset{ˆ}{\epsilon }}_{t}∥}^{2}\right],$$

where ${\overset{ˆ}{\epsilon }}_{t}$ is our network's noise prediction as described above and where we empirically choose to set w(t)=1 for the best possible generation results compared to w(t)=(1-SNR⁡(t-1)/SNR⁡(t)) with $SNR(t)=\alpha_{t}^{2}/\sigma_{t}^{2}$. Additionally, GCDM permits a negative log-likelihood computation using the same optimization terms as Hoogeboom et al. [20], for which we refer interested readers to Appendices A.2, A.3, and A.4 of our supplementary materials for remaining implementation details regarding how to compute model log-likelihoods and how to decide the number of atoms with which to generate a 3D molecular sample.

## 4 Discussion & Conclusions

While previous methods for 3D molecule generation have possessed insufficient geometric and molecular priors for scaling well to a variety of molecular datasets, in this work, we introduced a geometry-complete diffusion model (GCDM) that establishes a clear performance advantage over previous methods, generating more realistic, stable, valid, unique, and property-specific 3D molecules overall. Moreover, GCDM does so without complex modeling techniques such as latent diffusion, which suggests that GCDM's results could likely be further improved by expanding upon these techniques (Xu et al. [22]). Although GCDM's results here are promising, since it (like previous methods) requires fully-connected graph attention as well as 1,000 time steps to generate a batch of 3D molecules, using it to generate several thousand large molecules can take a notable amount of time (e.g., 15 minutes to generate 250 new large molecules). As such, future research with GCDM could involve adding new time-efficient graph construction or sampling algorithms (Song et al. [40]) or exploring the impact of higher-order (e.g., type-2 tensor) geometric expressiveness on 3D generative models to accelerate sample generation and increase sample quality. Furthermore, integrating additional external tools for assessing the quality and rationality of generated molecules (Harris et al. [41]) is a promising direction for future work.

## Supplementary Material

1

## Figures and Tables

**Table 1:** Comparison of GCDM with baseline methods for 3D molecule generation. The results are reported in terms of the negative log-likelihood (NLL)- log⁡p(x,h,N), atom stability, molecule stability, validity, and uniqueness of 10,000 samples drawn from each model, with standard deviations for each model across three runs on QM9. The top-1 (best) results for this task are in **bold**, and the second-best results are underlined.

Type	Method	NLL ↓	AS (%) ↑	MS (%) ↑	Val (%) ↑	Val and Uniq (%) ↑
NF	E-NF	−59.7	85.0	4.9	40.2	39.4
Generative GNN	G-Schnet	-	95.7	68.1	85.5	80.3
DDPM	GDM	−94.7	97.0	63.2	-	-
	GDM-aug	−92.5	97.6	71.6	90.4	89.5
	EDM	−110.7 ± 1.5	98.7 ± 0.1	82.0 ± 0.4	91.9 ± 0.5	90.7 ± 0.6
	Bridge	-	98.7 ± 0.1	81.8 ± 0.2	-	90.2
	Bridge + Force	-	98.8 ± 0.1	84.6 ± 0.3	92.0	90.7
LDM	GraphLDM	-	97.2	70.5	83.6	82.7
	GraphLDM-aug	-	97.9	78.7	90.5	89.5
	GeoLDM	-	**98.9** ± 0.1	**89.4** ± 0.5	93.8 ± 0.4	92.7 ± 0.5
GC-DDPM - *Ours*	GCDM w/o Frames	−162.3 ± 0.3	98.4 ± 0.0	81.7 ± 0.5	93.9 ± 0.1	92.7 ± 0.1
	GCDM w/o SMA	−131.3 ± 0.8	95.7 ± 0.1	51.7 ± 1.4	83.1 ± 1.7	82.8 ± 1.7
	GCDM	**−171.0** ± 0.2	98.7 ± 0.0	85.7 ± 0.4	**94.8** ± 0.2	**93.3** ± 0.0
Data			99.0	95.2	97.7	97.7

**Table 2:** Comparison of GCDM with baseline methods for property-conditional 3D molecule generation. The results are reported in terms of the mean absolute error for molecular property prediction by an EGNN classifier $\phi_{c}$ on a QM9 subset, with results listed for GCDM-generated samples as well as for four separate baseline methods. The top-1 (best) results for this task are in **bold**, and the second-best results are underlined.

Task	α	Δϵ	$\epsilon_{HOMO}$	$\epsilon_{LUMO}$	μ	$C_{v}$
Units	${Bohr}^{3}$	meV	meV	meV	D	$\frac{cal}{mol}K$
Naive (Upper-bound)	9.01	1470	645	1457	1.616	6.857
# Atoms	3.86	866	426	813	1.053	1.971
EDM	2.76	655	356	584	1.111	1.101
GeoLDM	2.37	**587**	**340**	522	1.108	1.025
GCDM	**1.97**	602	344	**479**	**0.844**	**0.689**
QM9 (Lower-bound)	0.10	64	39	36	0.043	0.040

**Table 3:** Comparison of GCDM with baseline methods for 3D molecule generation. The results are reported in terms of each method's negative log-likelihood, atom stability, and molecule stability with standard deviations across three runs on GEOM-Drugs, each drawing 10,000 samples from the model. The top-1 (best) results for this task are in **bold**, and the second-best results are underlined.

Type	Method	NLL ↓	AS (%) ↑	MS (%) ↑
NF	E-NF	-	75.0	0.0
DDPM	GDM	−14.2	75.0	0.0
	GDM-aug	−58.3	77.7	0.0
	EDM	−137.1	81.3	0.0
	Bridge	-	81.0 ± 0.7	0.0
	Bridge + Force	-	82.4 ± 0.8	0.0
LDM	GraphLDM	-	76.2	0.0
	GraphLDM-aug	-	79.6	0.0
	GeoLDM	-	84.4	0.0
GC-DDPM - *Ours*	GCDM w/o Frames	769.7	88.0 ± 0.3	3.4 ± 0.3
	GCDM w/o SMA	3505.5	43.9 ± 3.6	0.1 ± 0.0
	GCDM	**−234.3**	**89.0** ± 0.8	**5.2** ± 1.1
Data			86.5	2.8

**Table 4:** Comparison of GCDM with baseline methods for property-guided 3D molecule optimization. The results are reported in terms of molecular stability (MS) and the mean absolute error for molecular property prediction by an EGNN classifier $\phi_{c}$ on a QM9 subset, with results listed for EDM and GCDM-optimized samples as well as two different molecule generation baselines (“EDM Samples” and “GCDM Samples”). The top-1 (best) results for this task are in **bold**, and the second-best results are underlined.

Task	α/MS	Δϵ/MS	$\epsilon_{HOMO}\;/\;MS$	$\epsilon_{LUMO}\;/\;MS$	μ/MS	$C_{v}\;/\;MS$
Units	${Bohr}^{3}\;/\;\%$	meV/%	meV/%	meV/%	D/%	$\frac{cal}{mol}K/\%$
EDM Samples (Mod. Stable)	4.91 / 82.9	1.24 / 82.9	0.55 / 82.9	1.23 / 82.9	1.40 / 82.9	2.84 / 82.9
EDM-Opt (on EDM Samples)	4.80 / 84.4	1.24 / **86.3**	0.55 / 84.4	1.24 / **85.2**	1.41 / 86.0	2.83 / 84.2
GCDM-Opt (on EDM Samples)	**4.76** / **85.2**	**1.22** / 84.0	**0.54** / **84.6**	**1.20** / 83.5	**1.36** / **88.1**	**2.71** / **84.3**
GCDM Samples (Highly Stable)	4.82 / **90.5**	1.19 / 90.5	0.54 / 90.5	1.24 / 90.5	1.32 / 90.5	2.82 / **90.5**
EDM-Opt (on GCDM Samples)	**4.67** / 89.0	1.19 / 90.8	0.54 / 90.8	1.24 / **91.2**	1.32 / **92.6**	**2.80** / 90.0
GCDM-Opt (on GCDM Samples)	4.71 / 90.1	**1.18** / **91.2**	**0.53** / **91.0**	**1.23** / 89.7	**1.30** / 91.3	2.81 / 90.1

**Table 5:** Evaluation of generated molecules for target protein pockets from the Binding MOAD (BM) and CrossDocked (CD) test datasets. Our proposed method, GCDM-SBDD, achieves the best results for the metrics listed in **bold** and the second-best results for the metrics underlined.

Dataset	Method	Vina (kcal/mol, ↓)	QED (↑)	SA (↑)	Lipinski (↑)	Diversity (↑)
BM	DiffSBDD-cond (C*α*)	−6.281 ± 1.81	0.486 ± 0.17	0.313 ± 0.09	4.637 ± 0.63	0.730 ± 0.04
	DiffSBDD-joint (C*α*)	−6.406 ± 5.13	0.512 ± 0.17	0.308 ± 0.09	4.681 ± 0.58	0.621 ± 0.16
	GCDM-SBDD-cond (C*α*) (Ours)	−6.250 ± 1.26	0.465 ± 0.18	**0.618** ± 0.11	4.661 ± 0.74	**0.803** ± 0.04
	GCDM-SBDD-joint (C*α*) (Ours)	−6.159 ± 1.44	0.459 ± 0.18	0.584 ± 0.11	4.609 ± 0.78	0.789 ± 0.04
	*Reference*	−8.328 ± 2.05	0.602 ± 0.15	0.336 ± 0.08	4.838 ± 0.37	–
CD	DiffSBDD-cond (C*α*)	−5.540 ± 1.57	0.460 ± 0.14	0.357 ± 0.09	4.821 ± 0.45	0.815 ± 0.06
	DiffSBDD-joint (C*α*)	−5.735 ± 1.80	0.427 ± 0.15	0.343 ± 0.09	4.789 ± 0.49	0.807 ± 0.07
	GCDM-SBDD-cond (C*α*) (Ours)	**−5.950** ± 1.54	0.457 ± 0.16	**0.640** ± 0.13	4.758 ± 0.61	0.797 ± 0.08
	GCDM-SBDD-joint (C*α*) (Ours)	−5.870 ± 1.37	0.458 ± 0.17	0.631 ± 0.11	4.702 ± 0.69	0.807 ± 0.05
	*Reference*	−6.871 ± 2.32	0.476 ± 0.20	0.728 ± 0.14	4.340 ± 0.14	–

## Data Availability

The data required to train new GCDM models or reproduce our results are available under a Creative Commons Attribution 4.0 International Public License at https://zenodo.org/record/7881981. Additionally, all pre-trained GCDM model checkpoints are available under a Creative Commons Attribution 4.0 International Public License at https://zenodo.org/record/7881986.

## References

[1] Rombach R., Blattmann A., Lorenz D., Esser P., Ommer B.: High-resolution image synthesis with latent diffusion models. In: Proceedings of the IEEE/CVF Conference on Computer Vision and Pattern Recognition, pp. 10684–10695 (2022)

[2] Kong Z., Ping W., Huang J., Zhao K., Catanzaro B.: Diffwave: A versatile diffusion model for audio synthesis. arXiv preprint arXiv:2009.09761 (2020)

[3] Peebles W., Radosavovic I., Brooks T., Efros A.A., Malik J.: Learning to learn with generative models of neural network checkpoints. arXiv preprint arXiv:2209.12892 (2022)

[4] Anand N., Achim T.: Protein structure and sequence generation with equivariant denoising diffusion probabilistic models. arXiv preprint arXiv:2205.15019 (2022)

[5] Guo Z., Liu J., Wang Y., Chen M., Wang D., Xu D., Cheng J.: Diffusion models in bioinformatics and computational biology. Nature Reviews Bioengineering (2023) 10.1038/s44222-023-00114-9 PMC10994218 38576453

[6] Xu M., Yu L., Song Y., Shi C., Ermon S., Tang J.: Geodiff: A geometric diffusion model for molecular conformation generation. arXiv preprint arXiv:2203.02923 (2022)

[7] Mudur N., Finkbeiner D.P.: Can denoising diffusion probabilistic models generate realistic astrophysical fields? arXiv preprint arXiv:2211.12444 (2022)

[8] Bronstein M.M., Bruna J., Cohen T., Veličković P.: Geometric deep learning: Grids, groups, graphs, geodesics, and gauges. arXiv preprint arXiv:2104.13478 (2021)

[9] Joshi C.K., Bodnar C., Mathis S.V., Cohen T., Liò P.: On the expressive power of geometric graph neural networks. arXiv preprint arXiv:2301.09308 (2023)

[10] Stärk H., Ganea O., Pattanaik L., Barzilay R., Jaakkola T.: Equibind: Geometric deep learning for drug binding structure prediction. In: International Conference on Machine Learning, pp. 20503–20521 (2022). PMLR

[11] Jumper J., Evans R., Pritzel A., Green T., Figurnov M., Ronneberger O., Tunyasuvunakool K., Bates R., Žídek A., Potapenko A., : Highly accurate protein structure prediction with alphafold. Nature 596(7873), 583–589 (2021) 34265844 10.1038/s41586-021-03819-2 PMC8371605

[12] Vaswani A., Shazeer N., Parmar N., Uszkoreit J., Jones L., Gomez A.N., Kaiser Ł., Polosukhin I.: Attention is all you need. Advances in neural information processing systems 30 (2017)

[13] Lin Z., Akin H., Rao R., Hie B., Zhu Z., Lu W., Smetanin N., Verkuil R., Kabeli O., Shmueli Y., : Evolutionary-scale prediction of atomic-level protein structure with a language model. Science 379(6637), 1123–1130 (2023) 36927031 10.1126/science.ade2574

[14] Thomas N., Smidt T., Kearnes S., Yang L., Li L., Kohlhoff K., Riley P.: Tensor field networks: Rotation-and translation-equivariant neural networks for 3d point clouds. arXiv preprint arXiv:1802.08219 (2018)

[15] Ramakrishnan R., Dral P.O., Rupp M., Von Lilienfeld O.A.: Quantum chemistry structures and properties of 134 kilo molecules. Scientific data 1(1), 1–7 (2014) 10.1038/sdata.2014.22 PMC4322582 25977779

[16] Anderson B., Hy T.S., Kondor R.: Cormorant: Covariant molecular neural networks. Advances in neural information processing systems 32 (2019)

[17] Satorras V.G., Hoogeboom E., Fuchs F.B., Posner I., Welling M.: E (n) equivariant normalizing flows. arXiv preprint arXiv:2105.09016 (2021)

[18] Landrum G., : Rdkit: A software suite for cheminformatics, computational chemistry, and predictive modeling. Greg Landrum 8 (2013)

[19] Gebauer N., Gastegger M., Schütt K.: Symmetry-adapted generation of 3d point sets for the targeted discovery of molecules. Advances in neural information processing systems 32 (2019)

[20] Hoogeboom E., Satorras V.G., Vignac C., Welling M.: Equivariant diffusion for molecule generation in 3d. In: International Conference on Machine Learning, pp. 8867–8887 (2022). PMLR

[21] Wu L., Gong C., Liu X., Ye M., Liu Q.: Diffusion-based molecule generation with informative prior bridges. arXiv preprint arXiv:2209.00865 (2022)

[22] Xu M., Powers A., Dror R., Ermon S., Leskovec J.: Geometric latent diffusion models for 3d molecule generation. arXiv preprint arXiv:2305.01140 (2023)

[23] Satorras V.G., Hoogeboom E., Welling M.: E (n) equivariant graph neural networks. In: International Conference on Machine Learning, pp. 9323–9332 (2021). PMLR

[24] Hu L., Benson M.L., Smith R.D., Lerner M.G., Carlson H.A.: Binding moad (mother of all databases). Proteins: Structure, Function, and Bioinformatics 60(3), 333–340 (2005) 10.1002/prot.20512 15971202

[25] Francoeur P.G., Masuda T., Sunseri J., Jia A., Iovanisci R.B., Snyder I., Koes D.R.: Three-dimensional convolutional neural networks and a cross-docked data set for structure-based drug design. Journal of chemical information and modeling 60(9), 4200–4215 (2020) 32865404 10.1021/acs.jcim.0c00411 PMC8902699

[26] Schneuing A., Du Y., Harris C., Jamasb A.R., Igashov I., Blundell T.L., Lio P., Gomes C.P., Welling M., Bronstein M.M., : Structure-based drug design with equivariant diffusion models (2022) 10.1038/s43588-024-00737-x PMC11659159 39653846

[27] Peng X., Luo S., Guan J., Xie Q., Peng J., Ma J.: Pocket2mol: Efficient molecular sampling based on 3d protein pockets. In: International Conference on Machine Learning, pp. 17644–17655 (2022). PMLR

[28] Bickerton G.R., Paolini G.V., Besnard J., Muresan S., Hopkins A.L.: Quantifying the chemical beauty of drugs. Nature chemistry 4(2), 90–98 (2012) 10.1038/nchem.1243 PMC3524573 22270643

[29] Ertl P., Schuffenhauer A.: Estimation of synthetic accessibility score of drug-like molecules based on molecular complexity and fragment contributions. Journal of cheminformatics 1, 1–11 (2009) 20298526 10.1186/1758-2946-1-8 PMC3225829

[30] Lipinski C.A.: Lead-and drug-like compounds: the rule-of-five revolution. Drug discovery today: Technologies 1(4), 337–341 (2004) 24981612 10.1016/j.ddtec.2004.11.007

[31] Tanimoto T.T.: Elementary Mathematical Theory of Classification and Prediction. International Business Machines Corp., ??? (1958)

[32] Bajusz D., Rácz A., Héberger K.: Why is tanimoto index an appropriate choice for fingerprint-based similarity calculations? Journal of cheminformatics 7(1), 1–13(2015) 26052348 10.1186/s13321-015-0069-3 PMC4456712

[33] Barron L.: Symmetry and molecular chirality. Chemical Society Reviews 15(2), 189–223 (1986)

[34] Ho J., Jain A., Abbeel P.: Denoising diffusion probabilistic models. Advances in Neural Information Processing Systems 33, 6840–6851 (2020)

[35] Segler M.H., Kogej T., Tyrchan C., Waller M.P.: Generating focused molecule libraries for drug discovery with recurrent neural networks. ACS central science 4(1), 120–131 (2018) 29392184 10.1021/acscentsci.7b00512 PMC5785775

[36] Jin W., Barzilay R., Jaakkola T.: Junction tree variational autoencoder for molecular graph generation. In: Dy J., Krause A. (eds.) Proceedings of the 35th International Conference on Machine Learning. Proceedings of Machine Learning Research, vol. 80, pp. 2323–2332. PMLR, ??? (2018). https://proceedings.mlr.press/v80/jin18a.html

[37] Du W., Zhang H., Du Y., Meng Q., Chen W., Zheng N., Shao B., Liu T.-Y.: SE(3) equivariant graph neural networks with complete local frames. In: Chaudhuri K., Jegelka S., Song L., Szepesvari C., Niu G., Sabato S. (eds.) Proceedings of the 39th International Conference on Machine Learning. Proceedings of Machine Learning Research, vol. 162, pp. 5583–5608. PMLR, ??? (2022). https://proceedings.mlr.press/v162/du22e.html

[38] Morehead A., Cheng J.: Geometry-complete perceptron networks for 3d molecular graphs. AAAI Workshop on Deep Learning on Graphs: Methods and Applications (2023) 10.1093/bioinformatics/btae087 PMC10904142 38373819

[39] Du W., Zhang H., Du Y., Meng Q., Chen W., Zheng N., Shao B., Liu T.-Y.: Se(3) equivariant graph neural networks with complete local frames. In: International Conference on Machine Learning, pp. 5583–5608 (2022). PMLR

[40] Song J., Meng C., Ermon S.: Denoising diffusion implicit models. arXiv preprint arXiv:2010.02502 (2020)

[41] Harris C., Didi K., Jamasb A.R., Joshi C.K., Mathis S.V., Lio P., Blundell T.: Benchmarking generated poses: How rational is structure-based drug design with generative models? arXiv preprint arXiv:2308.07413 (2023)

[42] Sohl-Dickstein J., Weiss E., Maheswaranathan N., Ganguli S.: Deep unsupervised learning using nonequilibrium thermodynamics. In: International Conference on Machine Learning, pp. 2256–2265 (2015). PMLR

[43] Kingma D., Salimans T., Poole B., Ho J.: Variational diffusion models. Advances in neural information processing systems 34, 21696–21707 (2021)

[44] Köhler J., Klein L., Noé F.: Equivariant flows: exact likelihood generative learning for symmetric densities. In: International Conference on Machine Learning, pp. 5361–5370 (2020). PMLR

[45] Walters W.P., Murcko M.: Assessing the impact of generative ai on medicinal chemistry. Nature biotechnology 38(2), 143–145 (2020) 10.1038/s41587-020-0418-2 32001834

[46] Urbina F., Lentzos F., Invernizzi C., Ekins S.: Dual use of artificial-intelligence-powered drug discovery. Nature Machine Intelligence 4(3), 189–191 (2022) 10.1038/s42256-022-00465-9 PMC9544280 36211133

[47] Elfwing S., Uchibe E., Doya K.: Sigmoid-weighted linear units for neural network function approximation in reinforcement learning. Neural Networks 107, 3–11 (2018) 29395652 10.1016/j.neunet.2017.12.012

[48] Loshchilov I., Hutter F.: Decoupled weight decay regularization. arXiv preprint arXiv:1711.05101 (2017)

[49] Falcon W.A.: Pytorch lightning. GitHub 3 (2019)

[50] Paszke A., Gross S., Massa F., Lerer A., Bradbury J., Chanan G., Killeen T., Lin Z., Gimelshein N., Antiga L., : Pytorch: An imperative style, high-performance deep learning library. Advances in neural information processing systems 32 (2019)

[51] Fey M., Lenssen J.E.: Fast graph representation learning with pytorch geometric. arXiv preprint arXiv:1903.02428 (2019)

[52] Yadan O.: Hydra - A framework for elegantly configuring complex applications. Github (2019). https://github.com/facebookresearch/hydra

