# Geometry-Complete Diffusion for 3D Molecule Generation and Optimization

**Published:** 2024-05-24

**Authors:** Alex Morehead, Jianlin Cheng

**Affiliations:** 1 Electrical Engineering & Computer Science, NextGen Precision Health, University of Missouri, Columbia, 65211, Missouri, USA.

**Keywords:** Geometric deep learning, Diffusion generative modeling, 3D molecules

## Abstract

**Motivation:**

Generative deep learning methods have recently been proposed for generating 3D molecules using equivariant graph neural networks (GNNs) within a denoising diffusion framework. However, such methods are unable to learn important geometric properties of 3D molecules, as they adopt molecule-agnostic and non-geometric GNNs as their 3D graph denoising networks, which notably hinders their ability to generate valid large 3D molecules.

**Results:**

In this work, we address these gaps by introducing the Geometry-Complete Diffusion Model (GCDM) for 3D molecule generation, which outperforms existing 3D molecular diffusion models by significant margins across conditional and unconditional settings for the QM9 dataset and the larger GEOM-Drugs dataset, respectively. Importantly, we demonstrate that GCDM’s generative denoising process enables the model to generate a significant proportion of valid and energetically-stable large molecules at the scale of GEOM-Drugs, whereas previous methods fail to do so with the features they learn. Additionally, we show that extensions of GCDM can not only effectively design 3D molecules for specific protein pockets but can be repurposed to consistently optimize the geometry and chemical composition of existing 3D molecules for molecular stability and property specificity, demonstrating new versatility of molecular diffusion models.

**Availability:**

Code and data are freely available on GitHub.

## 1 Introduction

Generative modeling has recently been experiencing a renaissance in modeling efforts driven largely by denoising diffusion probabilistic models (DDPMs). At a high level, DDPMs are trained by learning how to denoise a noisy version of an input example. For example, in the context of computer vision, Gaussian noise may be successively added to an input image with the goals of a DDPM in mind. We would then desire for a generative model of images to learn how to successfully distinguish between the original input image’s feature signal and the noise added to the image thereafter. If a model can achieve such outcomes, we can use the model to generate novel images by first sampling multivariate Gaussian noise and then iteratively removing, from the current state of the image, the noise predicted by the model. This classic formulation of DDPMs has achieved significant results in the space of image generation [1], audio synthesis [2], and even meta-learning by learning how to conditionally generate neural network checkpoints [3]. Furthermore, such an approach to generative modeling has expanded its reach to encompass scientific disciplines such as computational biology [4–8], computational chemistry [9–11], and computational physics [12].

Concurrently, the field of geometric deep learning (GDL) [13] has seen a sizeable increase in research interest lately, driven largely by theoretical advances within the discipline [14] as well as by novel applications of such methodology [15–18]. Notably, such applications even include what is considered by many researchers to be a solution to the problem of predicting 3D protein structures from their corresponding amino acid sequences [19]. Such an outcome arose, in part, from recent advances in sequence-based language modeling efforts [20, 21] as well as from innovations in equivariant neural network modeling [22].

However, it is currently unclear how the expressiveness of geometric neural networks impacts the ability of generative methods that incorporate them to faithfully model a geometric data distribution. In addition, it is currently unknown whether diffusion models for 3D molecules can be repurposed for important, real-world tasks without retraining or fine-tuning and whether geometric diffusion models are better equipped for such tasks. Toward this end, in this work, we provide the following findings.

- Neural networks that perform message-passing with geometric quantities enable diffusion generative models of 3D molecules to generate valid and energetically-stable large molecules whereas non-geometric message-passing networks fail to do so, where we introduce key computational metrics to enable such findings.
- Physical inductive biases such as invariant graph attention and molecular chirality both play important roles in diffusion-generating valid 3D molecules.
- Our newly-proposed Geometry-Complete Diffusion Model (GCDM), which is the first diffusion model to incorporate the above insights and achieve the ideal type of equivariance for 3D molecule generation (i.e., SE(3) equivariance), establishes new state-of-the-art (SOTA) results for conditional 3D molecule generation on the QM9 dataset as well as for unconditional molecule generation on the GEOM-Drugs dataset of large 3D molecules, for the latter more than doubling PoseBusters validity rates; generates more unique and novel small molecules for unconditional generation on the QM9 dataset; and achieves better Vina energy scores and more than twofold higher PoseBusters validity rates [23] for protein-conditioned 3D molecule generation.
- We further demonstrate that geometric diffusion models such as GCDM can consistently perform 3D molecule optimization for molecular stability as well as for specific molecular properties without requiring any retraining and can consistently do so whereas non-geometric diffusion models cannot.

## 2 Results

### 2.1 Unconditional 3D Molecule Generation - QM9

The first dataset used in our experiments, the QM9 dataset [24], contains molecular properties and 3D atom coordinates for 130k small molecules. Each molecule in QM9 can contain up to 29 atoms after hydrogen atoms are imputed for each molecule following dataset postprocessing as in Hoogeboom et al. [25]. For the task of 3D molecule generation, we train GCDM to unconditionally generate molecules by producing atom types (H, C, N, O, and F), integer atom charges, and 3D coordinates for each of the molecules’ atoms. Following Anderson et al. [26], we split QM9 into training, validation, and test partitions consisting of 100k, 18k, and 13k molecule examples, respectively.

#### Metrics.

We measure each method’s average negative log-likelihood (NLL) over the corresponding test dataset, for methods that report this quantity. Intuitively, a method achieving a lower test NLL compared to other methods indicates that the method can more accurately predict *denoised* pairings of atom types and coordinates for unseen data, implying that it has fit the underlying data distribution more precisely than other methods. In terms of molecule-specific metrics, we adopt the scoring conventions of Satorras et al. [27] by using the distance between atom pairs and their respective atom types to predict bond types (single, double, triple, or none) for all but one baseline method (i.e., E-NF). Subsequently, we measure the proportion of generated atoms that have the right valency (atom stability - AS) and the proportion of generated molecules for which all atoms are stable (molecule stability - MS). To offer additional insights into each method’s behavior for 3D molecule generation, we also report the validity (Val) of the generated molecules as determined by RDKit [28], the uniqueness of the generated molecules overall (Uniq), and whether the generated molecules pass each of the *de novo* chemical and structural validity tests (i.e., sanitizable, all atoms connected, valid bond lengths and angles, no internal steric clashes, flat aromatic rings and double bonds, low internal energy, correct valence, and kekulizable) proposed in the PoseBusters software suite [23] and adopted by recent works on molecule generation tasks [29, 30]. Each method’s results in the top half (bottom half) of Table 1 are reported as the mean and standard deviation (mean and Student’s t-distribution 95% confidence error intervals) (±) of each metric across three (five) test runs on QM9, respectively.

#### Baselines.

Besides including a reference point for molecule quality metrics using QM9 itself (i.e., Data), we compare GCDM (a geometry-complete DDPM - i.e., GC-DDPM) to 10 baseline models for 3D molecule generation, each trained and tested using the same corresponding QM9 splits for fair comparisons: G-Schnet [31]; Equivariant Normalizing Flows (E-NF) [27]; Graph Diffusion Models (GDM) [25] and their variations (i.e., GCM-aug); Equivariant Diffusion Models (EDM) [25]; Bridge and Bridge + Force [32]; latent diffusion models (LDMs) such as GraphLDM and its variation GraphLDM-aug [33]; as well as the state-of-the-art GeoLDM method [33]. Note that we specifically include these baselines as representative *implicit bond prediction* methods for which bonds are inferred using their generated molecules’ atom types and inter-atom distances, in contrast to *explicit bond prediction* approaches such as those of [34] and [35] for fair comparisons with our method. For each of such baseline methods, we report their results as curated by Wu et al. [32] and Xu et al. [33]. We further include two GCDM ablation models to more closely analyze the impact of certain key model components within GCDM. These two ablation models include GCDM without chiral and geometry-complete local frames ${ℱ}_{ij}$ (i.e., GCDM w/o Frames) and GCDM without scalar message attention (SMA) applied to each edge message (i.e., GCDM w/o SMA). In Section 3 as well as Appendices B.1 and C, we further discuss GCDM’s design, hyperparameters, and optimization with these model configurations.

#### Results.

In the top half of Table 1, we see that GCDM achieves the highest percentage of probable (NLL), valid, and unique molecules compared to all baseline methods, with AS and MS results marginally lower than those of GeoLDM yet with lower standard deviations. In the bottom half of Table 1, where we reevaluate GCDM and GeoLDM using 5 sampling runs and report 95% confidence intervals for each metric, GCDM generates 1.6% more RDKit-valid and unique molecules and 5.2% more novel molecules compared to GeoLDM, all while offering the best reported negative log-likelihood (NLL) for the QM9 test dataset. This result indicates that although GeoLDM offers novelty rates close to parity (i.e., 50%), GCDM nearly matches the stability and PB-validity rates of GeoLDM while yielding novel molecules nearly 60% of the time on average, suggesting that GCDM may prove more useful for accurately exploring the space of novel yet valid small molecules. Our ablation of SMA within GCDM demonstrates that, to generate stable 3D molecules, GCDM heavily relies on both being able to perform a lightweight version of fully-connected graph self-attention [20], which suggests avenues of future research that will be required to scale up such generative models to large biomolecules such as proteins. Additionally, removing geometric local frame embeddings from GCDM reveals that the inductive biases of molecular chirality and geometry-completeness are important contributing factors in GCDM achieving these SOTA results. Figure 2 illustrates PoseBusters-valid examples of QM9-sized molecules generated by GCDM, with the following corresponding SMILES strings from left to right: **(****a****)** [H]/N=C(\C#N)NCC, **(****b****)** CC[N]c1n[nH]c(=O)o1, **(****c****)** O=CCNC(=O)CCO, **(****d****)** C/N=c1/[nH]c(O)c(N)o1, **(****e****)** [H]/N=C(/C[C]([NH])OC)OC, and **(****f****)** Oc1coc2cnoc12.

### 2.2 Property-Conditional 3D Molecule Generation - QM9

#### Baselines.

Towards the practical use case of conditional generation of 3D molecules, we compare GCDM to existing E(3)-equivariant models, EDM [25] and GeoLDM [33], as well as to two naive baselines: ”Naive (Upper-bound)” where a molecular property classifier $\phi_{c}$ predicts molecular properties given a method’s generated 3D molecules and shuffled (i.e., random) property labels; and ”# Atoms” where one uses the numbers of atoms in a method’s generated 3D molecules to predict their molecular properties. For each baseline method, we report its mean absolute error (MAE) in terms of molecular property prediction by an ensemble of three EGNN classifiers $\phi_{c}$ [36] as reported in Hoogeboom et al. [25]. For GCDM, we train each conditional model by conditioning it on one of six distinct molecular property feature inputs - α, gap, homo, lumo, μ, and $C_{v}$ - for approximately 1,500 epochs using the QM9 validation split of Hoogeboom et al. [25] as the model’s training dataset and the QM9 training split of Hoogeboom et al. [25] as the corresponding EGNN classifier ensemble’s training dataset. Consequently, one can expect the gap between a method’s performance and that of ”QM9 (Lower-bound)” to decrease as the method more accurately generates property-specific molecules.

#### Results.

We see in Table 2 that GCDM achieves the best overall results compared to all baseline methods in conditioning on a given molecular property, with conditionally-generated samples shown in Figure 3 (Note: PSI4-computed property values [37] for (a) and (f) are 69.1 Bohr^3^ (energy: −402 a.u.) and 89.7 Bohr^3^ (energy: −419 a.u.), respectively, at the DFT/B3LYP/6-31G(2df,p) level of theory [24, 38]). In particular, as shown in the bottom half of this table, GCDM surpasses the MAE results of the SOTA GeoLDM method (by 19% on average) for all six molecular properties - α, gap, homo, lumo, μ, and $C_{v}$ - by 28%, 9%, 3%, 15%, 21%, and 35%, respectively, while nearly matching the PB-Valid rates of GeoLDM (similar to the results in Table 1). These results qualitatively and quantitatively demonstrate that, using geometry-complete diffusion, GCDM enables notably precise generation of 3D molecules with specific molecular properties (e.g., α - polarizability).

### 2.3 Unconditional 3D Molecule Generation - GEOM-Drugs

The second dataset used in our experiments, the GEOM-Drugs dataset, is a well-known source of large, 3D molecular conformers for downstream machine learning tasks. It contains 430k molecules, each with 44 atoms on average and with up to as many as 181 atoms after hydrogen atoms are imputed for each molecule following dataset postprocessing as in Hoogeboom et al. [25]. For this experiment, we collect the 30 lowest-energy conformers corresponding to a molecule and task each baseline method with generating new molecules with 3D positions and types for each constituent atom. Here, we also adopt the negative log-likelihood, atom stability, and molecule stability metrics as defined in Section 2.1 and train GCDM using the same hyperparameters as listed in Appendix C.2, with the exception of training for approximately 75 epochs on GEOM-Drugs.

#### Baselines.

In this experiment, we compare GCDM to several state-of-the-art baseline methods for 3D molecule generation on GEOM-Drugs. Similar to our experiments on QM9, in addition to including a reference point for molecule quality metrics using GEOM-Drugs itself (i.e., Data), here we also compare against E-NF, GDM, GDM-aug, EDM, Bridge along with its variant Bridge + Force, as well as GraphLDM, GraphLDM-aug, and GeoLDM. As in Section 2.1, each method’s results in the top half (bottom half) of the table are reported as the mean and standard deviation (mean and Student’s t-distribution 95% confidence interval) (±) of each metric across three (five) test runs on GEOM-Drugs.

#### Results.

To start, Table 3 displays an interesting phenomenon that is important to note: Due to the size and atomic complexity of GEOM-Drugs’ molecules and the subsequent errors accumulated when estimating bond types based on such inter-atom distances, the baseline results for the molecule stability metrics measured here (i.e., Data) are much lower than those collected for the QM9 dataset. Thus, reporting additional chemical and structural validity metrics (e.g., PB-Valid) for comparison is crucial to accurately assess a method’s performance in this context, which we do in the bottom half of Table 3. Nonetheless, for GEOM-Drugs, GCDM supersedes EDM’s SOTA negative log-likelihood results by 57% and advances GeoLDM’s SOTA atom and molecule stability results by 4% and more than sixfold, respectively. More importantly, however, GCDM can generate a significant proportion of PB-valid large molecules, surpassing even the reference molecule stability rate of the GEOM-Drugs dataset (i.e., 2.8) by 54%, demonstrating that geometric diffusion models such as GCDM can not only effectively generate valid large molecules but can also generalize beyond the native distribution of stable molecules within GEOM-Drugs.

Figure 4 illustrates PoseBusters-valid examples of large molecules generated by GCDM at the scale of GEOM-Drugs, with the following corresponding SMILES strings from left to right: **(****a****)** CC(C)=N[N]C(=O)O[C]([CH]C(=O)NCCCCc1cccnc1)Cc1ccc2c(c1)OCO2, **(****b****)** CN(N)Cc1cccnc1C(=O)NCCCc1ccc(F)cc1, **(****c****)** C=CCC(=O)c1cc(C(N)=O)c2ccccc2n1, **(****d****)** CC(=O)N/N=C/N=C/C=C\N=C(/O)[C](O)CC(=O)N(O)Cc1ccc(F)c(F)c1, **(****e****)** COC(=O)/C(CN)=C(\[CH]c1cc(C(C)=O)c(C)n1C)c1cc(Cl)ccc1O, and **(****f****)** CC[C@@H](C)/N=C/[C](N[N+](=O)[O−])C(=O)c1ccc(C(=O)O)cc1. As an example of the notion that GCDM produces low energy structures for a generated molecular graph, the free energies for Figures 4 (a) and (f) were computed to be −3 kcal/mol and −2 kcal/mol, respectively, using CREST [39] at the GFN2-xTB level of theory (which matches the corresponding free energy distribution mean for the GEOM-Drugs dataset (−2.5 kcal/mol) as illustrated in Figure 2 of [40]). Lastly, to detect whether a method, in aggregate, generates molecules with unlikely 3D conformations, a generated molecule’s energy ratio is defined as in Buttenschoen et al. [23] to be the ratio of the molecule’s UFF-computed energy [41] and the mean of 50 RDKit ETKDGv3-generated conformers [42] of the same molecular graph. Note that, as discussed by Wills et al. [43], generated molecules with an energy ratio greater than 7 are considered to have highly unlikely 3D conformations. Subsequently, Figure 5 reveals that the average energy ratio of GCDM’s large 3D molecules is notably lower and more tightly bounded compared to GeoLDM, the baseline SOTA method for this task, indicating that GCDM also generates more energetically-stable 3D molecule conformations compared to prior methods.

### 2.4 Property-Guided 3D Molecule Optimization - QM9

To evaluate whether molecular diffusion models can not only generate new 3D molecules but can also optimize existing small molecules using molecular property guidance, we adopt the QM9 dataset for the following experiment. First, we use an unconditional GCDM model to generate 1,000 3D molecules using 10 time steps of time-scaled reverse diffusion (to leave such molecules in an *unoptimized* state), and then we provide these molecules to a separate property-conditional diffusion model for optimization of the molecules towards the conditional model’s respective property. This conditional model accepts these 3D molecules as intermediate states for 100 and 250 time steps of property-guided optimization of the molecules’ atom types and 3D coordinates. Lastly, we repurpose our experimental setup from Section 2.2 to score these optimized molecules using an ensemble of external property classifier models to evaluate (1) how much the optimized molecules’ predicted property values have been improved for the respective property (first metric) and (2) whether and how much the optimized molecules’ stability (as defined in Section 2.1) has been changed during optimization (second metric).

#### Baselines.

Baseline methods for this experiment include EDM [25] and GCDM, where both methods use similar experimental setups for evaluation. Our baseline methods also include property-specificity and molecule stability measures of the initial (unconditional) 3D molecules to demonstrate how much molecular diffusion models can modify or improve these existing 3D molecules in terms of how property-specific and stable they are. As in Section 2.2, property specificity is measured in terms of the corresponding property classifier’s MAE for a given molecule with a targeted property value, reporting the mean and Student’s t-distribution 95% confidence interval for each property MAE across an ensemble of three corresponding classifiers. Molecular stability (i.e., Mol Stable (%)), here abbreviated at *MS*, is defined as in Section 2.1.

#### Results.

Figure 6 showcases a practical finding: geometric diffusion models such as GCDM can effectively be repurposed as 3D molecule optimization methods with minimal modifications, improving both a molecule’s stability and property specificity. This finding empirically supports the idea that molecular denoising diffusion models approximate the Boltzmann distribution with the score function they learn [44] and therefore may be applied in the optimization stage of the typical drug discovery pipeline [45] to experiment with a wider range of potential drug candidates (post-optimization) more quickly than previously possible. Simultaneously, the baseline EDM method fails to consistently optimize the stability and property specificity of existing 3D molecules, which suggests that geometric methods such as GCDM are theoretically and empirically better suited for such tasks. Notably, on average, with 100 time steps GCDM improves the stability of the initial molecules by over 25% and their specificity for each molecular property by over 27%, whereas for the properties it can optimize with 100 time steps, EDM improves the stability of the molecules by 13% and their property specificity by 15%. Lastly, it is worth noting that increasing the number of optimization time steps from 100 to 250 steps inconsistently leads to further improvements to molecules’ stability and property specificity, indicating that the optimization trajectory likely reaches a local minimum around 100 time steps and hence rationalizes reducing the required compute time for optimizing 1,000 molecules e.g., from 15 minutes (for 250 steps) to 5 minutes (for 100 steps).

### 2.5 Protein-Conditional 3D Molecule Generation

To investigate whether geometry-complete methods can enhance the ability of molecular diffusion models to generate 3D models within a given protein pocket (i.e., to perform structure-based drug design (SBDD)), in this experiment, we adopt the standard Binding MOAD (BM) [46] and CrossDocked (CD) [47] datasets for training and evaluation of GCDM-SBDD, our geometry-complete, diffusion generative model based on GCPNet++ that extends the diffusion framework of Schneuing et al. [48] for protein pocket-aware molecule generation. The Binding MOAD dataset consists of 100,000 high-quality protein-ligand complexes for training and 130 proteins for testing, with a 30% sequence identity threshold being used to define this cross-validation split. Similarly, the CrossDocked dataset contains 40,484 high-quality protein-ligand complexes split between training (40,354) and test (100) partitions using proteins’ enzyme commission numbers as described by Schneuing et al. [48].

#### Baselines.

Baseline methods for this experiment include DiffSBDD-cond [48] and DiffSBDD-joint [48]. We compare these methods to our proposed geometry-complete protein-aware diffusion model, GCDM-SBDD, using metrics that assess the properties, and thereby the quality, of each method’s generated molecules. These molecule-averaged metrics include a method’s average Vina score (computed using QuickVina 2.1) [49] as a physics-based estimate of a ligand’s estimated binding affinity with a target protein, measured in units of kcal/mol (lower is better); average drug likeliness QED [50] (computed using RDKit 2022.03.2); average synthesizability [51] (computed using the procedure introduced by [52]) as an increasing measure of the ease of synthesizing a given molecule (higher is better); on average how many rules of Lipinski’s rule of five are satisfied by a ligand [53] (computed compositionally using RDKit 2022.03.2); and average diversity in mean pairwise Tanimoto distances [54, 55] (derived manually using fingerprints and Tanimoto similarities computed by RDKit 2022.03.2). Following established conventions for 3D molecule generation [25], the size of each ligand to generate was determined using the ligand size distribution of the respective training dataset. Note that, in this context, ”joint” and ”cond” configurations represent generating a molecule for a protein target, respectively, with and without also modifying the coordinates of the binding pocket within the protein target. Also note that, similar to our experiments in Sections 2.1–2.4, the GCDM-SBDD model uses 9 GCP message-passing layers along with 256 (64) and 32 (16) invariant (equivariant) node and edge features, respectively.

#### Results.

Table 4 shows that, across both of the standard SBDD datasets (i.e., Binding MOAD and CrossDocked), GCDM-SBDD generates more clash-free (PB-Valid) and lower energy (Vina) molecules compared to prior methods. Moreover, across all other metrics, GCDM-SBDD achieves comparable or better results in terms of drug-likeness measures (e.g., QED) and comparable results for all other molecule metrics *without performing any hyperparameter tuning due to compute constraints*. These results suggest that GCDM, with GCPNet++ as its denoising neural network, not only works well for de novo 3D molecule generation but also protein target-specific 3D molecule generation, notably expanding the number of real-world application areas of GCDM. Concretely, GCDM-SBDD improves upon DiffSBDD’s average Vina energy scores by 8% on average across both datasets while generating more than twice as many PB-valid ”candidate” molecules for the more challenging Binding MOAD dataset.

As suggested by [23], the gap between the PB-Valid ratios in Table 4 without and with protein-ligand steric clashes considered for both GCDM-SBDD and DiffSBDD suggests that deep learning-based drug design methods for targeted protein pockets can likely benefit significantly from interaction-aware molecular dynamics relaxation following protein-conditional molecule generation, which may allow for many generated ”candidate” molecules to have their PB validity ”recovered” by such relaxation. Nonetheless, Figure 7 demonstrates that GCDM can consistently generate clash-free realistic and diverse 3D molecules with low Vina energies for unseen protein targets.

## 3 Methods

### 3.1 Problem Setting

In this work, our goal is to generate new 3D molecules either unconditionally or conditioned on user-specified properties. We represent a molecular point cloud (e.g., 3D molecule) as a fully-connected 3D graph 𝒢=(𝒱,ℰ) with 𝒱 and ℰ representing the graph’s sets of nodes and edges, respectively, and N=|𝒱| and E=|ℰ| representing the numbers of nodes and edges in the graph, accordingly. In addition, $X=\left(x_{1},x_{2},\ldots ,x_{N}\right)\in R^{N\times 3}$ represents the respective Cartesian coordinates for each node (i.e., atom). Each node in 𝒢 is described by scalar features $H\in R^{N\times h}$ and m vector-valued features $\chi \in R^{N\times (m\times 3)}$. Likewise, each edge in 𝒢 is described by scalar features $E\in R^{E\times e}$ and x vector-valued features $\xi \in R^{E\times (x\times 3)}$. Then, let ℳ=[X,H] represent the molecules (i.e., atom coordinates and atom types) our method is tasked with generating, where [·,·] denotes the concatenation of two variables. Important to note is that the input features H and E are *invariant* to 3D roto-translations, whereas the input vector features X, χ and ξ are *equivariant* to 3D roto-translations. Lastly, in particular, we design a denoising neural network Φ to be equivariant to 3D roto-translations (i.e., SE(3)-equivariant) by defining it such that its internal operations and outputs match corresponding 3D roto-translations acting upon its inputs.

### 3.2 Overview of GCDM

We will now introduce GCDM, a new Geometry-Complete SE(3)-Equivariant Diffusion Model. GCDM defines a joint noising process on equivariant atom coordinates x and invariant atom types h to produce a noisy representation $z=\left[z^{\left(x\right)},\;z^{\left(h\right)}\right]$ and then learns a generative *denoising* process using the newly-proposed GCPNet++ model (see Section A.2 of the appendix), which desirably contains two distinct feature channels for scalar and vector features, respectively, and supports geometry-complete and chirality-aware message-passing [56].

As an extension of the DDPM framework [57] outlined in Appendix B.1, GCDM is designed to generate molecules in 3D while maintaining SE(3) equivariance, in contrast to previous methods that generate molecules solely in 1D [58], 2D [59], or 3D modalities without considering chirality [9, 25]. GCDM generates molecules by directly placing atoms in continuous 3D space and assigning them discrete types, which is accomplished by modeling forward and reverse diffusion processes, respectively:

$$\underset{\text{Forward}\;}{\underbrace{q\left(z_{1:T}|z_{0}\right)}}=\prod_{t=1}^{T}q\left(z_{t}|z_{t-1}\right)\;\underset{\text{Reverse}\;}{\underbrace{p_{\Phi }\left(z_{0:T-1}|z_{T}\right)}}=\prod_{t=1}^{T}p_{\Phi }\left(z_{t-1}|z_{t}\right)$$

**Fig. 1:**
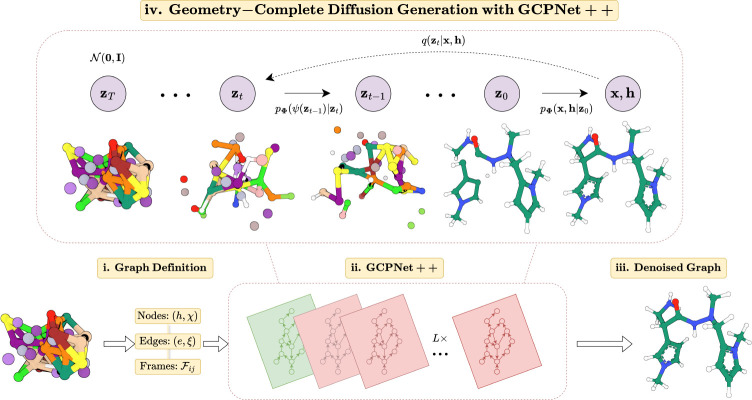
A framework overview of the proposed *Geometry-Complete Diffusion Model* (GCDM) for geometric and chirality-aware 3D molecule generation. The framework consists of (**i.**) a graph (topology) definition process; (**ii.**) a GCPNet-based graph neural network for SE(3)-equivariant graph representation learning; (**iii.**) denoising of 3D input graphs using GCPNet++; and (**iv.**) application of a trained GCPNet++ denoising network for 3D molecule generation. Zoom in for the best viewing experience.

**Fig. 2:**
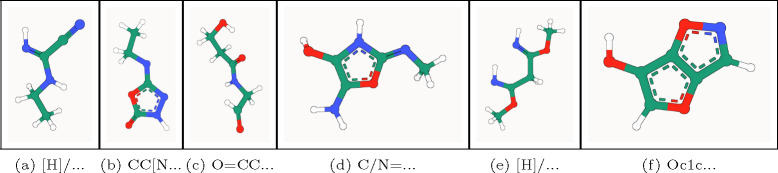
PB-valid 3D molecules generated by GCDM for the QM9 dataset.

**Fig. 3:**
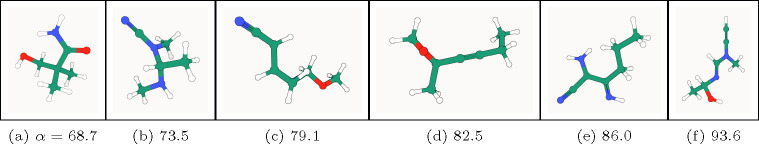
PB-valid 3D molecules generated by GCDM using increasing values of α.

**Fig. 4:**
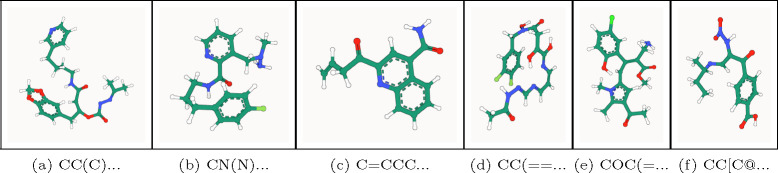
PB-valid 3D molecules generated by GCDM for the GEOM-Drugs dataset.

**Fig. 5:**
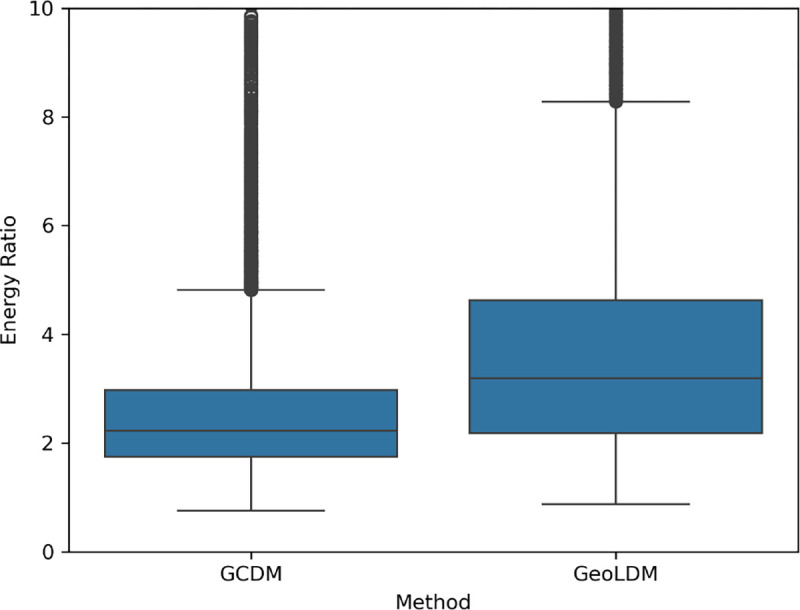
A comparison of the energy ratios [23] of 10,000 large 3D molecules generated by GCDM and GeoLDM, a baseline state-of-the-art method. Employing Student’s t-distribution 95% confidence intervals, GCDM achieves a mean energy ratio of 2.98 ± 0.13, whereas GeoLDM yields a mean energy ratio of 4.19 ± 0.09.

**Fig. 6:**
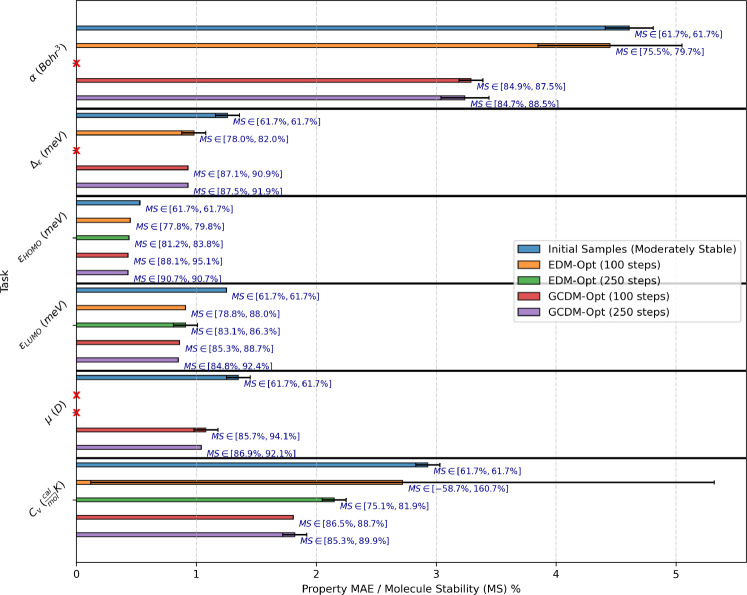
Comparison of GCDM with baseline methods for property-guided 3D molecule optimization. The results are reported in terms of molecular stability (MS) and the MAE for molecular property prediction by an ensemble of three EGNN classifiers $\phi_{c}$ (each trained on the same QM9 subset using a distinct random seed) yielding corresponding Student’s t-distribution 95% confidence intervals, with results listed for EDM and GCDM-optimized samples as well as the molecule generation baseline (”Initial Samples”). Note that x denotes a missing bar representing outlier property MAEs greater than 50. Alternatively, tabular results are given in Table C1 of the appendix.

**Fig. 7:**
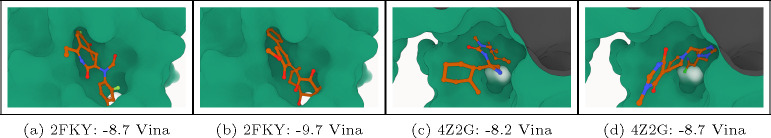
GCDM-SBDD molecules generated for BM (a-b) and CD (c-d) test proteins.

Overall, these processes describe a latent variable model $p_{\Phi }\left(z_{0}\right)=\int p_{\Phi }\left(z_{0:T}\right)dz_{1:T}$ given a sequence of latent variables $z_{0},z_{1},\ldots ,z_{T}$ matching the dimensionality of the data $ℳ∼p\left(z_{0}\right)$. As illustrated in Figure 1, the forward process (directed from right to left) iteratively adds noise to an input, and the learned reverse process (directed from left to right) iteratively denoises a noisy input to generate new examples from the original data distribution. We will now proceed to formulate GCDM’s joint diffusion process and its remaining practical details.

### 3.3 Joint Molecular Diffusion

Recall that our model’s molecular graph inputs, 𝒢, associate with each node a 3D position $x_{i}\in R^{3}$ and a feature vector $h_{i}\in R^{h}$. By way of adding random noise to these model inputs at each time step t via a fixed, Markov chain variance schedule $\sigma_{1}^{2},\sigma_{2}^{2},\ldots ,\sigma_{T}^{2}$, we can define a joint molecular diffusion process for equivariant atom coordinates x and invariant atom types h as the product of two distributions [25]:

(1) $$q\left(z_{t}|z_{t-1}\right)={𝒩}_{xh}\left(z_{t}|\alpha_{t}z_{t-1},\sigma_{t}^{2}I\right).$$

where ${𝒩}_{xh}$ serves as concise notation to denote the product of two normal distributions; the first distribution, ${𝒩}_{x}$, represents the noised node coordinates; the second distribution, ${𝒩}_{h}$, represents the noised node features; and $\alpha_{t}=\sqrt{1-\sigma_{t}^{2}}$ following the variance preserving process of Ho et al. [57]. With $\alpha_{t|s}=\alpha_{t}/\alpha_{s}$ and $\sigma_{t|s}^{2}=\sigma_{t}^{2}-\alpha_{t|s}\sigma_{s}^{2}$ for any t>s, we can directly obtain the noisy data distribution $q\left(z_{t}|z_{0}\right)$ at any time step t:

(2) $$q\left(z_{t}|z_{0}\right)={𝒩}_{xh}(z_{t}|\alpha_{t|0}z_{0},\sigma_{t|0}^{2}I).$$

Bayes Theorem then tells us that if we then define $\mu_{t\to s}\left(z_{t},z_{0}\right)$ and $\sigma_{t\to s}$ as

$$\mu_{t\to s}\left(z_{t},z_{0}\right)=\frac{\alpha_{s}\sigma_{t|s}^{2}}{\sigma_{t}^{2}}z_{0}+\frac{\alpha_{t|s}\sigma_{s}^{2}}{\sigma_{t}^{2}}z_{t}\;\text{and}\;\sigma_{t\to s}=\frac{\sigma_{t|s}\sigma_{s}}{\sigma_{t}},$$

we have that the inverse of the noising process, the *true denoising process*, is given by the posterior of the transitions conditioned on $ℳ∼z_{0}$, a process that is also Gaussian [25]:

(3) $$q\left(z_{s}|z_{t},z_{0}\right)=𝒩\left(z_{s}|\mu_{t\to s}\left(z_{t},z_{0}\right),\sigma_{t\to s}^{2}I\right).$$

### 3.4 Parametrization of the Reverse Process

#### Noise parametrization.

We now need to define the learned generative reverse process that *denoises* pure noise into realistic examples from the original data distribution. Towards this end, we can directly use the noise posteriors $q\left(z_{s}|z_{t},z_{0}\right)$ of Eq. B12 in the appendix after sampling $z_{0}∼(ℳ=[x,\;h])$. However, to do so, we must replace the input variables x and h with the approximations $\overset{ˆ}{x}$ and $\overset{ˆ}{h}$ predicted by the denoising neural network Φ:

(4) $$p_{\Phi }\left(z_{s}|z_{t}\right)={𝒩}_{xh}\left(z_{s}|\mu_{\Phi_{t\to s}}\left(z_{t},{\tilde{z}}_{0}\right),\sigma_{t\to s}^{2}I\right),$$

where the values for ${\tilde{z}}_{0}=[\overset{ˆ}{x},\overset{ˆ}{h}]$ depend on $z_{t}$, t, and the denoising neural network Φ. GCDM then parametrizes $\mu_{\Phi_{t\to s}}\left(z_{t},{\tilde{z}}_{0}\right)$ to predict the noise $\overset{ˆ}{\epsilon }=[{\overset{ˆ}{\epsilon }}^{\left(x\right)},{\overset{ˆ}{\epsilon }}^{\left(h\right)}]$, which represents the noise individually added to $\overset{ˆ}{x}$ and $\overset{ˆ}{h}$. We can then use the predicted $\overset{ˆ}{\epsilon }$ to derive:

(5) $${\tilde{z}}_{0}=[\overset{ˆ}{x},\overset{ˆ}{h}]=z_{t}/\alpha_{t}-{\overset{ˆ}{\epsilon }}_{t}\cdot \sigma_{t}/\alpha_{t}.$$

#### Invariant likelihood.

Ideally, we desire for a 3D molecular diffusion model to assign the same likelihood to a generated molecule even after arbitrarily rotating or translating it in 3D space. To ensure the model achieves this desirable property for $p_{\Phi }\left(z_{0}\right)$, we can leverage the insight that an invariant distribution composed of an equivariant transition function yields an invariant distribution [9, 25, 27]. Moreover, to address the translation invariance issue raised by Satorras et al. [27] in the context of handling a distribution over 3D coordinates, we adopt the zero center of gravity trick proposed by Xu et al. [9] to define ${𝒩}_{x}$ as a normal distribution on the subspace defined by $\sum_{i}x_{i}=0$. In contrast, to handle node features $h_{i}$ that are invariant to roto-translations, we can instead use a conventional normal distribution 𝒩. As such, if we parametrize the transition function $p_{\Phi }$ using an SE(3)-equivariant neural network after using the zero center of gravity trick of Xu et al. [9], the model will have achieved the desired likelihood invariance property.

### 3.5 Geometry-Complete Denoising Network

Crucially, to satisfy the desired likelihood invariance property described in Section 3.4 while optimizing for model expressivity and runtime, GCDM parametrizes the denoising neural network Φ using GCPNet++, an enhanced version of the SE(3)-equivariant GCPNet algorithm [56], that we propose in Section A.2 of the appendix. Notably, GCPNet++ learns both scalar (invariant) and vector (equivariant) node and edge features through a chirality-sensitive graph message passing procedure, which enables GCDM to denoise its noisy molecular graph inputs using not only noisy scalar features but also noisy *vector* features that are derived directly from the noisy node coordinates $z^{\left(x\right)}$ (i.e., $\psi \left(z^{\left(x\right)}\right)$). We empirically find that incorporating such noisy vectors considerably increases GCDM’s representation capacity for 3D graph denoising.

### 3.6 Optimization Objective

Following previous works on diffusion models [25, 32, 57], the noise parametrization chosen for GCDM yields the following model training objective:

(6) $${ℒ}_{t}=E_{\epsilon_{t}∼{𝒩}_{xh}(0,1)}\left[\frac{1}{2}w(t){∥\epsilon_{t}-{\overset{ˆ}{\epsilon }}_{t}∥}^{2}\right],$$

where ${\overset{ˆ}{\epsilon }}_{t}$ is the denoising network’s noise prediction for atom types and coordinates as described above and where we empirically choose to set w(t)=1 for the best possible generation results. Additionally, GCDM permits a negative log-likelihood computation using the same optimization terms as Hoogeboom et al. [25], for which we refer interested readers to Appendices B.2, B.3, and B.4 of the appendix.

## 4 Discussion & Conclusions

While previous methods for 3D molecule generation have possessed insufficient geometric and molecular priors for scaling well to a variety of molecular datasets, in this work, we introduced a geometry-complete diffusion model (GCDM) that establishes a clear performance advantage over previous methods, generating more realistic, stable, valid, unique, and property-specific 3D molecules, while enabling the generation of many large 3D molecules that are energetically stable as well as chemically and structurally valid. Moreover, GCDM does so without complex modeling techniques such as latent diffusion, which suggests that GCDM’s results could likely be further improved by expanding upon these techniques [33]. Although GCDM’s results here are promising, since it (like previous methods) requires fully-connected graph attention as well as 1,000 time steps to generate a high-quality batch of 3D molecules, using it to generate several thousand large molecules can take a notable amount of time (e.g., 15 minutes to generate 250 new large molecules). As such, future research with GCDM could involve adding new time-efficient graph construction or sampling algorithms [60] or exploring the impact of higher-order (e.g., type-2 tensor) yet efficient geometric expressiveness [61] on 3D generative models to accelerate sample generation and increase sample quality. Furthermore, integrating additional external tools for assessing the quality and rationality of generated molecules [62] is a promising direction for future work.

## Figures and Tables

**Table 1:** Comparison of GCDM with baseline methods for 3D molecule generation.

Type	Method	NLL ↓	AS (%) ↑	MS (%) ↑	Val (%) ↑	Val and Uniq (%) ↑

NF	E-NF	−59.7	85.0	4.9	40.2	39.4

Generative GNN	G-Schnet	-	95.7	68.1	85.5	80.3

DDPM	GDM	−94.7	97.0	63.2	-	-
	GDM-aug	−92.5	97.6	71.6	90.4	89.5
	EDM	−110.7 ± 1.5	98.7 ± 0.1	82.0 ± 0.4	91.9 ± 0.5	90.7 ± 0.6
	Bridge	-	98.7 ± 0.1	81.8 ± 0.2	-	90.2
	Bridge + Force	-	98.8 ± 0.1	84.6 ± 0.3	92.0	90.7

LDM	GraphLDM	-	97.2	70.5	83.6	82.7
	GraphLDM-aug	-	97.9	78.7	90.5	89.5
	GeoLDM	-	**98.9** ± 0.1	**89.4** ± 0.5	93.8 ± 0.4	92.7 ± 0.5

GC-DDPM - *Ours*	GCDM w/o Frames	−162.3 ± 0.3	98.4 ± 0.0	81.7 ± 0.5	93.9 ± 0.1	92.7 ± 0.1
	GCDM w/o SMA	−131.3 ± 0.8	95.7 ± 0.1	51.7 ± 1.4	83.1 ± 1.7	82.8 ± 1.7
	GCDM	**−171.0** ± 0.2	98.7 ± 0.0	85.7 ± 0.4	**94.8** ± 0.2	**93.3** ± 0.0

Data			99.0	95.2	97.7	97.7

Method	NLL ↓	AS (%) ↑	MS (%) ↑	Val (%) ↑	Val and Uniq (%) ↑	Novel (%) ↑	PB-Valid (%) ↑

GeoLDM	-	**98.9** ± 0.0	**89.8** ± 0.4	93.6 ± 0.2	91.8 ± 0.2	53.5 ± 0.6	**93.1** ± 0.4

GCDM	**−169.4** ± 0.8	98.7 ± 0.1	86.0 ± 0.7	**94.9** ± 0.3	**93.4** ± 0.3	**58.7** ± 0.5	91.9 ± 0.5

The results in the top half of the table are reported in terms of the negative log-likelihood (NLL) − log *p*(**x**, **h**, *N*), atom stability, molecule stability, validity, and uniqueness of 10,000 samples drawn from each model, with standard deviations (±) for each model across three runs on QM9. The results in the bottom half of the table are for methods specifically evaluated across *five* runs on QM9 using Student’s t-distribution 95% confidence intervals for per-metric errors, additionally with novelty (Novel) defined as the percentage of (valid and unique) generated molecule SMILES strings that were not found in the QM9 dataset and PoseBusters validity (PB-Valid) defined as the percentage of generated molecules that pass all relevant *de novo* structural and chemical sanity checks listed in Section 2.1. The top-1 (best) results for this task are in **bold**, and the second-best results are underlined, with - denoting a metric value that is not available.

**Table 2:** Comparison of GCDM with baseline methods for property-conditional 3D molecule generation.

Task	*α* ↓	Δ*ϵ* ↓	*ϵ_HOMO_* ↓	*ϵ_LUMO_* ↓	*μ* ↓	*C_v_* ↓
Units	*Bohr* ^3^	*meV*	*meV*	*meV*	*D*	$\frac{cal}{mol}K$

Naive (Upper-bound)	9.01	1470	645	1457	1.616	6.857
# Atoms	3.86	866	426	813	1.053	1.971
EDM	2.76	655	356	584	1.111	1.101
GeoLDM	2.37	**587**	**340**	522	1.108	1.025
GCDM	**1.97**	602	344	**479**	**0.844**	**0.689**

QM9 (Lower-bound)	0.10	64	39	36	0.043	0.040

Task	*α* ↓	Δ*ϵ* ↓	*ϵ_HOMO_* ↓	*ϵ_LUMO_* ↓	*μ* ↓	*C_v_* ↓
Units	*Bohr* ^3^	*meV*	*meV*	*meV*	*D*	$\frac{cal}{mol}K$

**GeoLDM**	2.77 ± 0.12	655 ± 20.57	357 ± 5.68	565 ± 10.62	1.089 ± 0.02	1.070 ± 0.04
**GCDM**	**1.99** ± 0.01	**595** ± 14.34	**346** ± 1.23	**480** ± 6.58	**0.855** ± 0.00	**0.698** ± 0.01

Metric	*α* PB-Valid (%) ↑	Δ*ϵ* PB-Valid (%) ↑	*ϵ_HOMO_* PB-Valid (%) ↑	*ϵ_LUMO_* PB-Valid (%) ↑	*μ* PB-Valid (%) ↑	*C_v_* PB-Valid (%) ↑

GeoLDM	93.7 ± 0.5	92.8 ± 0.3	93.9 ± 0.4	93.3 ± 0.6	93.2 ± 1.3	92.5 ± 0.8
GCDM	92.3 ± 0.3	92.5 ± 0.8	92.7 ± 0.5	92.7 ± 0.6	92.4 ± 0.4	91.7 ± 0.4

The results in the top half of the table are reported in terms of the MAE for molecular property prediction by an EGNN classifier $\phi_{c}$ on a QM9 subset, with results listed for GCDM-generated samples as well as for four separate baseline methods. The results in the bottom half of the table (where GeoLDM is retrained using its official code repository due to the unavailability of its conditional model checkpoints) are likewise listed for selected methods yet instead report (across an ensemble of three separately-trained EGNN property classifier models, each with a distinct random seed) Student’s t-distribution 95% confidence error intervals for each property metric as well as the percentage of PoseBusters-validated (PB-Valid) de novo generated molecules. The top-1 (best) conditioning results for this task are in **bold**, and the second-best results are underlined.

**Table 3:** Comparison of GCDM with baseline methods for 3D molecule generation.

Type	Method	NLL ↓	AS (%) ↑	MS (%) ↑

NF	E-NF	-	75.0	0.0

DDPM	GDM	−14.2	75.0	0.0
	GDM-aug	−58.3	77.7	0.0
	EDM	−137.1	81.3	0.0
	Bridge	-	81.0 ± 0.7	0.0
	Bridge + Force	-	82.4 ± 0.8	0.0

LDM	GraphLDM	-	76.2	0.0
	GraphLDM-aug	-	79.6	0.0
	GeoLDM	-	84.4	0.0

GC-DDPM - *Ours*	GCDM w/o Frames	769.7	88.0 ± 0.3	3.4 ± 0.3
	GCDM w/o SMA	3505.5	43.9 ± 3.6	0.1 ± 0.0
	GCDM	**−234.3**	**89.0** ± 0.8	**5.2** ± 1.1

Data			86.5	2.8

Method	NLL ↓	AS (%) ↑	MS (%) ↑	Val (%) ↑	Val and Uniq (%) ↑	Novel (%) ↑	PB-Valid (%) ↑

GeoLDM	-	84.4 ± 0.1	0.6 ± 0.1	**99.5** ±0.1	**99.4** ± 0.1	-	38.3 ± 0.5
GCDM	**−215.1** ± 3.8	**88.1** ± 0.1	**4.3** ± 0.4	95.5 ± 0.1	95.5 ± 0.1	**95.5** ± 0.1	**77.0** ± 0.1

The results in the top half of the table are reported in terms of each method’s negative log-likelihood, atom stability, and molecule stability with standard deviations (±) across three runs on GEOM-Drugs, each drawing 10,000 samples from the model. The results in the bottom half of the table are for methods specifically evaluated across *five* runs on QM9 using Student’s t-distribution 95% confidence intervals for per-metric errors, additionally with validity and uniqueness (Val and Uniq), novelty (Novel), and PoseBusters validity (PB-Valid) defined likewise as in Section 2.1; The top-1 (best) results for this task are in **bold**, and the second-best results are underlined.

**Table 4:** Evaluation of generated molecules for target protein pockets from the Binding MOAD (BM) and CrossDocked (CD) test datasets.

Dataset	Method Vina (kcal/mol, ↓)	QED (↑)	SA (↑)	Lipinski (↑)	Diversity (↑)	PB-Valid (%) (↑)

BM DiffSBDD-cond (C*α*)	−5.784 ± 0.03	0.433 ± 0.00	0.616 ± 0.00	4.719 ± 0.01	0.848 ± 0.00	16.6 ± 0.6 / 1.7 ± 0.2
DiffSBDD-joint (C*α*)	−5.882 ± 0.05	0.474 ± 0.00	0.631 ± 0.00	4.835 ± 0.01	0.852 ± 0.00	10.7 ± 0.5 / 0.7 ± 0.1
GCDM-SBDD-cond (C*α*) (Ours)	**−6.250** ± 0.03	0.465 ± 0.00	0.618 ± 0.00	4.661 ± 0.01	0.806 ± 0.00	**40.8** ± 0.8 / **6.8** ± 0.4
GCDM-SBDD-joint (C*α*) (Ours)	−6.159 ± 0.06	0.459 ± 0.00	0.584 ± 0.00	4.609 ± 0.02	0.794 ± 0.00	37.3 ± 0.8 / 2.0 ± 0.2
*Reference*	−8.328 ± 0.04	0.602 ± 0.00	0.336 ± 0.00	4.838 ± 0.01	–	–

CD DiffSBDD-cond (C*α*)	−5.540 ± 0.03	0.449 ± 0.00	0.636 ± 0.00	4.735 ± 0.01	0.818 ± 0.00	40.7 ± 1.0 / 12.4 ± 0.6
DiffSBDD-joint (C*α*)	−5.735 ± 0.05	0.420 ± 0.00	0.662 ± 0.00	4.859 ± 0.01	0.890 ± 0.00	34.1 ± 0.9 / 6.2 ± 0.5
GCDM-SBDD-cond (C*α*) (Ours)	**−5.955** ± 0.04	0.457 ± 0.00	0.640 ± 0.00	4.758 ± 0.02	0.795 ± 0.00	38.1 ± 1.0 / **15.7** ± 0.7
GCDM-SBDD-joint (C*α*) (Ours)	−5.870 ± 0.03	**0.458** ± 0.00	0.631 ± 0.00	4.701 ± 0.02	0.810 ± 0.00	**46.8** ± 1.0 / 6.5 ± 0.5
*Reference*	−6.871 ± 0.04	0.476 ± 0.00	0.728 ± 0.00	4.340 ± 0.00	–	–

Our proposed method, GCDM-SBDD, achieves the best results for the metrics listed in **bold** and the second-best results for the metrics underlined. For each metric, a method’s mean and Student’s t-distribution 95% confidence error interval (±) is reported over 100 generated molecules for each test pocket. Additionally, the PoseBusters validity (PB-Valid) metric is defined as the percentage of generated molecules that pass all *docking*-relevant structural and chemical sanity checks proposed by [23], with the validity ratio to the left (right) of each / denoting the percentage of valid molecules without (with) consideration of protein-ligand steric clashes.

## Data Availability

The data required to train new GCDM models or reproduce our results are available under a Creative Commons Attribution 4.0 International Public License at https://zenodo.org/record/7881981. Additionally, all pre-trained model checkpoints are available under a Creative Commons Attribution 4.0 International Public License at https://zenodo.org/record/10995319.

## Appendix A Expanded Discussion of Denoising

### A.1 Geometry-Complete Denoising

In this section, we postulate that certain types of geometric neural networks serve as more effective 3D graph denoising functions for molecular DDPMs. We describe this notion as follows.

#### Hypothesis A.1. (Geometry-Complete Denoising).

Geometric neural networks that achieve geometry-completeness are more robust in denoising 3D molecular network inputs compared to models that are not geometry-complete, in that geometry-complete methods unambiguously define direction-robust local geometric reference frames.

This hypothesis comes as an extension of the definition of geometry-completeness from Du et al. [63] and Morehead and Cheng [56]:

#### Definition A.2. (Geometric Completeness).

Given a pair of node positions $\left(x_{i}^{t},x_{j}^{t}\right)$ in a 3D graph 𝒢, with vectors $a_{ij}^{t}\in R^{1\times 3}$, $b_{ij}^{t}\in R^{1\times 3}$, and $c_{ij}^{t}\in R^{1\times 3}$ derived from $\left(x_{i}^{t},x_{j}^{t}\right)$, a local geometric representation ${ℱ}_{ij}^{t}=(a_{ij}^{t},b_{ij}^{t},c_{ij}^{t})\in R^{3\times 3}$ is considered geometrically complete if ${ℱ}_{ij}^{t}$ is non-degenerate, hence forming a *local orthonormal basis* located at the tangent space of $x_{i}^{t}$.

An intuition for the implications of Hypothesis A.1 and Definition A.2 on molecular diffusion models is that geometry-complete networks should be able to more effectively learn the gradients of data distributions [57] in which a global force field is present, as is typically the case with 3D molecules [63]. This is because, broadly speaking, geometry-complete methods encode local reference frames for each node (or edge) under which the directions of arbitrary global force vectors can be mapped. In addition to describing the theoretical benefits offered to geometry-complete denoising networks, we support this hypothesis through specific ablation studies in Sections 2.1 and 2.3 where we ablate the geometric frame encodings from GCDM and find that such frames are particularly useful in improving GCDM’s ability to generate realistic 3D molecules.

### A.2 GCPNet++

Inspired by its recent success in modeling 3D molecular structures with geometry-complete message-passing, we parametrize $p_{\Phi }$ using an enhanced version of Geometry-Complete Perceptron Networks (GCPNets) that were originally introduced by Morehead and Cheng [56]. To summarize, GCPNet is a geometry-complete graph neural network that is equivariant to SE(3) transformations of its graph inputs and maps nicely to the context of Hypothesis A.1.

In this setting, with $\left(h_{i}\in H,\chi_{i}\in \chi ,e_{ij}\in E,\xi_{ij}\in \xi \right)$, GCPNet++, our enhanced version of GCPNet, consists of a composition of Geometry-Complete Graph Convolution (GCPConv) layers $\left(h_{i}^{l},\chi_{i}^{l}\right),x_{i}^{l}=GCPConv\left[(h_{i}^{l-1},\chi_{i}^{l-1}),\;(e_{ij}^{l-1},\xi_{ij}^{l-1}),x_{i}^{l-1},{ℱ}_{ij}\right]$ which are defined as:

(A1)
$$n_{i}^{l}=\phi^{l}(n_{i}^{l-1},{𝒜}_{\forall j\in 𝒩(i)}\Omega_{\omega }^{l}(n_{i}^{l-1},n_{j}^{l-1},e_{ij}^{l-1},\xi_{ij}^{l-1},{ℱ}_{ij})),$$

where $n_{i}^{l}=\left(h_{i}^{l},\chi_{i}^{l}\right);$ $\phi^{l}$ is a trainable function; l signifies the representation depth of the network; 𝒜 is a permutation-invariant aggregation function; $\Omega_{\omega }$ represents a message-passing function corresponding to the *ω*-th GCP message-passing layer [56]; and node i’s geometry-complete local frames are ${ℱ}_{ij}^{t}=(a_{ij}^{t},b_{ij}^{t},c_{ij}^{t})$, with $a_{ij}^{t}=\frac{x_{i}^{t}-x_{j}^{t}}{∥x_{i}^{t}-x_{j}^{t}∥}$, $b_{ij}^{t}=\frac{x_{i}^{t}\times x_{j}^{t}}{∥x_{i}^{t}\times x_{j}^{t}∥}$, and $c_{ij}^{t}=a_{ij}^{t}\times b_{ij}^{t}$, respectively. Importantly, GCPNet++ restructures the network flow of GCPConv [56] for each iteration of node feature updates to simplify and enhance information flow, concretely from the form of

(A2)
$${\overset{ˆ}{n}}^{l}=n^{l-1}+f(\Omega_{\omega ,v_{i}}^{l}|v_{i}\in 𝒱)$$

to

(A3)
$${\overset{ˆ}{n}}^{l}=n^{l-1}\cup f((g_{e^{\omega },v_{i}}^{l},\Omega_{e^{\omega },v_{i}}^{l},\Omega_{\xi^{\omega },v_{i}}^{l})|v_{i}\in 𝒱)$$

and from

(A4)
$$n^{l}={ResGCP}_{r}^{l}\left({\tilde{n}}_{r-1}^{l}\right)$$

to

(A5)
$$n^{l}={GCP}_{r}^{l}\left({\tilde{n}}_{r-1}^{l}\right).$$

Note that here f represents a summation or a mean function that is invariant to node order permutations; ∪ denotes the concatenation operation; $g_{e^{\omega },v_{i}}^{l}$ represents the binary-valued (i.e., [0, 1]) output of a scalar message attention (gating) function, expressed as

A6
$$g_{e^{\omega }}^{l}=\sigma_{inf}(\phi_{inf}^{l}(\Omega_{e^{\omega }}^{l}))$$

with $\phi_{\inf}\;:R^{e}\to [0,\;1{]}^{1}$^1^ mapping from high-dimensional scalar edge feature space to a single dimension and σ denoting a sigmoid activation function; r is the node feature update module index; ResGCP is a version of the GCP module with added residual connections; and $\Omega_{\omega ,v_{i}}^{l}=(\Omega_{e^{\omega },v_{i}}^{l},\Omega_{\xi^{\omega },v_{i}}^{l})$ represents the scalar (e) and vector-valued (ξ) messages derived with respect to node $v_{i}$ using up to ω message-passing iterations within each GCPNet++ layer.

We found these adaptations to provide state-of-the-art molecule generation results compared to the original node feature updating scheme, which we found yielded suboptimal results in the context of generative modeling. This highlights the importance of customizing representation learning algorithms for the generative modeling task at hand, since reasonable performance may not always be achievable with them without careful adaptations. It is worth noting that, since GCPNet++ performs message-passing directly on 3D vector features, GCDM is thereby the **first** diffusion generative model that is in principle capable of generating 3D molecules with specific *vector*-valued properties. We leave a full exploration of this idea for future work.

### A.3 Properties of GCDM

If one desires to update the coordinate representations of each node in 𝒢, as we do in the context of 3D molecule generation, the GCPConv module of GCPNet++ provides a simple, SE(3)-equivariant method to do so using a dedicated GCP module as follows:

(A7)
$$\left(h_{p_{i}}^{l},\chi_{p_{i}}^{l}\right)={GCP}_{p}^{l}\left(n_{i}^{l},{ℱ}_{ij}\right)$$

(A8)
$$x_{i}^{l}=x_{i}^{l-1}+\chi_{p_{i}}^{l}\text{,}\;\text{where}\;\chi_{p_{i}}^{l}\in R^{1\times 3},$$

where ${GCP}_{.}^{l}\left(\cdot ,{ℱ}_{ij}\right)$ is defined to provide chirality-aware rotation and translation-invariant updates to $h_{i}$ and rotation-equivariant updates to $\chi_{i}$ following centralization of the input point cloud’s coordinates X [63]. The effect of using positional feature updates $\chi_{p_{i}}$ to update $x_{i}$ is, after decentralizing X following the final GCPConv layer, that updates to $x_{i}$ then become SE(3)-equivariant. As such, all transformations described above satisfy the required equivariance constraints. Therefore, in integrating GCPNet++ as its 3D graph denoiser, GCDM achieves SE(3) equivariance, geometry-completeness, and likelihood invariance altogether. Important to note is that GCDM subsequently performs message-passing with vector features to denoise its geometric inputs, whereas previous methods denoise their inputs **solely** using geometrically-insufficient scalar message-passing [14] as we demonstrate through our experiments in Section 2.

## Appendix B Expanded Discussion of Diffusion

### B.1 Diffusion Models

Key to understanding the contributions in this work are denoising diffusion probabilistic models (DDPMs). As alluded to previously, once trained, DDPMs can generate new data of arbitrary shapes, sizes, formats, and geometries by learning to reverse a noising process acting on each model input. More precisely, for a given data point x, a diffusion process adds noise to x for time step t=0,1,…,T to yield $z_{t}$, a noisy representation of the input x at time step t. Such a process is defined by a multivariate Gaussian distribution:

(B9)
$$q\left(z_{t}|x\right)=𝒩\left(z_{t}|\alpha_{t}x_{t},\sigma_{t}^{2}I\right),$$

where $\alpha_{t}\in R^{+}$ regulates how much feature signal is retained and $\sigma_{t}^{2}$ modulates how much feature noise is added to input x. Note that we typically model α as a function defined with smooth transitions from $\alpha_{0}=1$ to $\alpha_{T}=0$, where a special case of such a noising process, the variance preserving process [57, 64], is defined by $\alpha_{t}=\sqrt{1-\sigma_{t}^{2}}$. To simplify notation, in this work, we define the feature signal-to-noise ratio as $SNR(t)=\alpha_{t}^{2}/\sigma_{t}^{2}$. Also interesting to note is that this diffusion process is Markovian in nature, indicating that we have transition distributions as follows:

B10
$$q\left(z_{t}|z_{s}\right)=𝒩(z_{t}|\alpha_{t|s}z_{s},\sigma_{t|s}^{2}I),$$

for all t>s with $\alpha_{t|s}=\alpha_{t}/\alpha_{s}$ and $\sigma_{t|s}^{2}=\sigma_{t}^{2}-\alpha_{t|s}^{2}\sigma_{s}^{2}$. In total, then, we can write the noising process as:

(B11)
$$q\left(z_{0},z_{1},\ldots ,z_{T}|x\right)=q\left(z_{0}|x\right)\prod_{t=1}^{T}q\left(z_{t}|z_{t-1}\right).$$

If we then define $\mu_{t\to s}\left(x,z_{t}\right)$ and $\sigma_{t\to s}$ as

$$\mu_{t\to s}\left(x,z_{t}\right)=\frac{\alpha_{t|s}\sigma_{s}^{2}}{\sigma_{t}^{2}}z_{t}+\frac{\alpha_{s}\sigma_{t|s}^{2}}{\sigma_{t}^{2}}x\;\text{and}\;\sigma_{t\to s}=\frac{\sigma_{t|s}\sigma_{s}}{\sigma_{t}},$$

we have that the inverse of the noising process, the *true denoising process*, is given by the posterior of the transitions conditioned on x, a process that is also Gaussian:

(B12)
$$q\left(z_{s}|x,z_{t}\right)=𝒩\left(z_{s}|\mu_{t\to s}\left(x,z_{t}\right),\sigma_{t\to s}I\right).$$

#### The Generative Denoising Process.

In diffusion models, we define the generative process according to the *true denoising process*. However, for such a denoising process, we do not know the value of x *a priori*, so we typically approximate it as $\overset{ˆ}{x}=\phi \left(z_{t},t\right)$ using a neural network ϕ. Doing so then lets us express the generative transition distribution $p\left(z_{s}|z_{t}\right)$ as $q\left(z_{s}|\overset{ˆ}{x}\left(z_{t},t\right),z_{t}\right)$. As a practical alternative to Eq. B12, we can represent this expression using the approximation for $\overset{ˆ}{x}$:

(B13)
$$p\left(z_{s}|z_{t}\right)=𝒩\left(z_{s}|\mu_{t\to s}\left(\overset{ˆ}{x},z_{t}\right),\sigma_{t\to s}^{2}I\right).$$

If we choose to define s as s=t−1, then we can derive the variational lower bound on the log-likelihood of x given the generative model as:

(B14)
$$\text{log}\;p\left(\text{x}\right)\geq {ℒ}_{0}+{ℒ}_{\mathrm{base}\;}+\sum_{t=1}^{T}{ℒ}_{t},$$

where we note that ${ℒ}_{0}=log\;p\left(x|z_{0}\right)$ models the likelihood of the data given its noisy representation $z_{0}$, ${ℒ}_{\mathrm{base}}=-KL\left(q\left(z_{T}|x\right)|p\left(z_{T}\right)\right)$ models the difference between a standard normal distribution and the final latent variable $q\left(z_{T}|x\right)$, and

$${ℒ}_{t}=-\mathrm{KL}\left(q\left(z_{s}|x,z_{t}\right)|p\left(z_{s}|z_{t}\right)\right)\;\text{for}\;t=1,\;2,\ldots ,T.$$

Note that, in this formation of diffusion models, the neural network ϕ directly predicts $\overset{ˆ}{x}$. However, Ho et al. [57] and others have found optimization of ϕ to be made much easier when instead predicting the Gaussian noise added to x to create $\overset{ˆ}{x}$. An intuition for how this changes the neural network’s learning dynamics is that, when predicting back the noise added to the model’s input, the network is being trained to more directly differentiate which part of $z_{t}$ corresponds to the input’s feature signal (i.e., the underlying data point x) and which part corresponds to added feature noise. In doing so, if we let $z_{t}=\alpha_{t}x+\sigma_{t}\epsilon$, the neural network can then predict $\overset{ˆ}{\epsilon }=\phi \left(z_{t},t\right)$ such that:

(B15)
$$\overset{ˆ}{x}=\left(1/\alpha_{t}\right)\;z_{t}-\left(\sigma_{t}/\alpha_{t}\right)\;\overset{ˆ}{\epsilon }.$$

Kingma et al. [65] and others have since shown that, when parametrizing the denoising neural network in this way, the loss term ${ℒ}_{t}$ reduces to:

B16
$${ℒ}_{t}=E_{\epsilon ∼𝒩(0,I)}\left[\frac{1}{2}(1-SNR(t-1)/SNR(t))∥\epsilon -\overset{ˆ}{\epsilon }{∥}^{2}\right]$$

Note that, in practice, the loss term ${ℒ}_{\mathrm{base}}$ should be close to zero when using a noising schedule defined such that $\alpha_{T}\approx 0$. Moreover, if and when $\alpha_{0}\approx 1$ and x is a discrete value, we will find ${ℒ}_{0}$ to be close to zero as well.

### B.2 Zeroth Likelihood Terms for GCDM Optimization Objective

For the zeroth likelihood terms corresponding to each type of input feature, we directly adopt the respective terms previously derived by Hoogeboom et al. [25]. Doing so enables a negative log-likelihood calculation for GCDM’s predictions. In particular, for integer node features, we adopt the zeroth likelihood term:

(B17)
$$p(h|z_{0}^{\left(h\right)})=\int_{h-\frac{1}{2}}^{h+\frac{1}{2}}𝒩(u|z_{0}^{\left(h\right)},\sigma_{0})du,$$

where we use the CDF of a standard normal distribution, Φ, to compute Eq. B17 as $\Phi ((h+\frac{1}{2}-z_{0}^{\left(h\right)})/\sigma_{0})-\Phi ((h-\frac{1}{2}-z_{0}^{\left(h\right)})/\sigma_{0})\approx 1$ for reasonable noise parameters $\alpha_{0}$ and $\sigma_{0}$ [25]. For categorical node features, we instead use the zeroth likelihood term:

(B18)
$$p\left(h|z_{0}^{\left(h\right)}\right)=C\left(h|p\right),p\propto \int_{1-\frac{1}{2}}^{1+\frac{1}{2}}𝒩\left(u|z_{0}^{\left(h\right)},\sigma_{0}\right)du,$$

where we normalize p to sum to one and where C is a categorical distribution [25]. Lastly, for continuous node positions, we adopt the zeroth likelihood term:

(B19)
$$p(x|z_{0}^{\left(x\right)})=𝒩(x|z_{0}^{\left(x\right)}/\alpha_{0}-\sigma_{0}/\alpha_{0}{\overset{ˆ}{\epsilon }}_{0},\sigma_{0}^{2}/\alpha_{0}^{2}I)$$

which gives rise to the log-likelihood component ${ℒ}_{0}^{\left(x\right)}$ as:

(B20)
$${ℒ}_{0}^{\left(x\right)}=E_{\epsilon^{\left(x\right)}∼{𝒩}_{x}(0,I)}\left[logZ^{-1}-\frac{1}{2}{∥\epsilon^{x}-\phi^{\left(x\right)}\left(z_{0},0\right)∥}^{2}\right],$$

where d=3 and the normalization constant $Z={\left(\sqrt{2\pi }\cdot \sigma_{0}/\alpha_{0}\right)}^{(N-1)\cdot d}$ - in particular, its (N−1)⋅d term - arises from the zero center of gravity trick mentioned in Section 3.4 of the main text [25].

### B.3 Diffusion Models and Equivariant Distributions

In the context of diffusion generative models of 3D data, one often desires for the marginal distribution p(x) of their denoising neural network to be an invariant distribution. Towards this end, we observe that a conditional distribution p(y∣x) is equivariant to the action of 3D rotations by meeting the criterion:

(B21)
$$p\left(y|x\right)=p\left(Ry|Rx\right)\;\text{for}\;\text{all}\;\text{orthogonal}\;R.$$

Moreover, a distribution is invariant to rotation transformations R when

$$p\left(y\right)=p\left(Ry\right)\;\text{for}\;\text{all}\;\text{orthogonal}\;R.$$

As Köhler et al. [66] and Xu et al. [9] have collectively demonstrated, we know that if $p\left(z_{T}\right)$ is invariant and the neural network we use to parametrize $p\left(z_{t-1}|z_{t}\right)$ is equivariant, we have, as desired, that the marginal distribution p(x) of the denoising model is an invariant distribution.

### B.4 Training and Sampling Procedures for GCDM

#### Equivariant Dynamics.

In this work, we use the previous definition of GCPNet++ in Section A.2 of the main text to learn an SE(3)-equivariant dynamics function $\left[{\overset{ˆ}{\epsilon }}^{\left(x\right)},{\overset{ˆ}{\epsilon }}^{\left(h\right)}]=\phi (z_{t}^{\left(x\right)},z_{t}^{\left(h\right)},t\right)$ as:

(B23)
$${\overset{ˆ}{\epsilon }}_{t}^{\left(x\right)},{\overset{ˆ}{\epsilon }}_{t}^{\left(h\right)}=GCPNET++(z_{t}^{\left(x\right)},(z_{t}^{\left(h\right)},\psi \left(z_{t}^{\left(x\right)}\right),t/T))-[z_{t}^{\left(x\right)},0],$$

where we inform the denoising model of the current time step by concatenating t/T as an additional node feature and where we subtract the coordinate representation outputs of GCPNet++ from its coordinate representation inputs after subtracting from the coordinate representation outputs their collective center of gravity. Lastly yet importantly, as a geometric GNN, GCPNet++ can embed geometric vector features in addition to scalar features. Subsequently, from the noisy coordinates representation $z_{t}^{\left(x\right)}$ we derive noisy sequential (node) orientation unit vectors and pairwise (edge) displacement unit vectors $\psi (z_{t}^{\left(x\right)})$, respectively, and embed these features using GCPNet++’s vector feature channels for nodes and edges accordingly. With the parametrization in Eq. 5 of the main text, GCDM subsequently achieves rotation equivariance on ${\overset{ˆ}{x}}_{i}$, thereby achieving a 3D translation and rotation-invariant marginal distribution p(x) as described in Appendix B.3.

#### Scaling Node Features.

In line with Hoogeboom et al. [25], to improve the log-likelihood of the model’s generated samples, we find it useful to train and perform sampling with GCDM using scaled node feature inputs as $\left[x,\;\frac{1}{4}h^{\text{(}\mathrm{categorical})},\frac{1}{10}h^{\text{(}\mathrm{integer}\text{)}}\right]$.

#### Deriving The Number of Atoms.

Finally, to determine the number of atoms with which GCDM will generate a 3D molecule, we first sample N∼p(N), where p(N) denotes the categorical distribution of molecule sizes over GCDM’s training dataset. Then, we conclude by sampling x,h∼p(x,h∣N).

## Appendix C Additional Details

### C.1 Broader Impacts

In this work, we investigate the impact of geometric representation learning on generative models for 3D molecules. Such research can contribute to drug discovery efforts by accelerating the development of new medicinal or energy-related molecular compounds, and, as a consequence, can yield positive societal impacts [67]. Nonetheless, in line with Urbina et al. [68], we authors would argue that it will be critical for institutions, governments, and nations to reach a consensus on the strict regulatory practices that should govern the use of such molecule design methodologies in settings in which it is reasonably likely such methodologies could be used for nefarious purposes by scientific ”bad actors”.

### C.2 Training Details

#### Scalar Message Attention.

In our implementation of scalar message attention (SMA) within GCDM, $m_{ij}=e_{ij}m_{ij}$, where $m_{ij}$ represents the scalar messages learned by GCPNet++ during message-passing and $e_{ij}$ represents a 1 if an edge exists between nodes i and j (and a 0 otherwise) via $e_{ij}\approx \phi_{\inf}\left(m_{ij}\right)$. Here, $\phi_{\inf}\;:\;R^{e}\to [0,\;1{]}^{1}$ resembles a linear layer followed by a sigmoid function [36].

#### GCDM Hyperparameters.

All GCDM models train on QM9 for approximately 1,000 epochs using 9 **GCPConv** layers; SiLU activations [69]; 256 and 64 scalar node and edge hidden features, respectively; and 32 and 16 vector-valued node and edge features, respectively. All GCDM models are also trained using the AdamW optimizer [70] with a batch size of 64, a learning rate of 10^−4^, and a weight decay rate of 10^−12^.

#### GCDM Runtime.

With a maximum batch size of 64, this 9-layer model configuration allows us to train GCDM models for unconditional (conditional) tasks on the QM9 dataset using approximately 10 (15) days of GPU training time with a single 24GB NVIDIA A10 GPU. For unconditional molecule generation on the much larger GEOM-Drugs dataset, a maximum batch size of 64 allows us to train 4-layer GCDM models using approximately 60 days of GPU training time with a single 48GB NVIDIA RTX A6000 GPU. As such, access to several GPUs with larger GPU memory limits (e.g., 80GBs) should allow one to concurrently train GCDM models in a fraction of the time via larger batch sizes or data-parallel training techniques [71].

### C.3 Compute Requirements

Training GCDM models for tasks on the QM9 dataset by default requires a GPU with at least 24GB of GPU memory. Inference with such GCDM models for QM9 is much more flexible in terms of GPU memory requirements, as users can directly control how soon a molecule generation batch will complete according to the size of molecules being generated as well as one’s selected batch size during sampling. Training GCDM models for unconditional molecule generation on the GEOM-Drugs dataset by default requires a GPU with at least 48GB of GPU memory. Similar to the GCDM models for QM9, inference with GEOM-Drugs models is flexible in terms of GPU memory requirements according to one’s choice of sampling hyperparameters. Note that inference for both QM9 models and GEOM-Drugs models can likely be accelerated using techniques such as DDIM sampling [60]. However, we have not officially validated the quality of generated molecules using such sampling techniques, so we caution users to be aware of this potential risk of degrading molecule sample quality when using such sampling algorithms.

**Table C1:** Comparison of GCDM with baseline methods for property-guided 3D molecule optimization.

Task	*α* ↓ / *MS* ↑	Δ*ϵ* ↓ / *MS* ↑	*ϵ_HOMO_* ↓ / *MS* ↑	*ϵ_LUMO_* ↓ / *MS* ↑	*μ* ↓ / *MS* ↑	*C_v_* ↓ / *MS* ↑
Units	*Bohr*^3^ / %	*meV* / %	*meV* / %	*meV* / %	*D* / %	$\frac{\;\mathrm{cal}\;}{\;\mathrm{mol}}K/\text{\%}$

Initial Samples (Moderately Stable)	4.61 ± 0.2 / 61.7	1.26 ± 0.1 / 61.7	0.53 ± 0.0 / 61.7	1.25 ± 0.0 / 61.7	1.35 ± 0.1 / 61.7	2.93±0.1/61.7

EDM-Opt (100 steps on initial samples)	4.45 ± 0.6 / 77.6 ± 2.1	0.98 ± 0.1 / 80.0 ± 2.0	0.45 ± 0.0 / 78.8 ± 1.0	0.91 ± 0.0 / 83.4 ± 4.6	6*e*^5^ ± 6*e*^5^ / 78.3 ± 2.9	2.72 ± 2.6 / 51.0 ± 109.7
EDM-Opt (250 steps on initial samples)	1*e*^2^ ± 5*e*^2^ / 80.1 ± 2.1	1*e*^3^ ± 6*e*^3^ / 83.7 ± 3.8	0.44 ± 0.0 / 82.5 ± 1.3	0.91 ± 0.1 / 84.7 ± 1.6	2*e*^5^ ± 8*e*^5^ / 81.0 ± 5.8	2.15 ± 0.1 / 78.5 ± 3.4

GCDM-Opt (100 steps on initial samples)	3.29 ± 0.1 / 86.2 ± 1.3	0.93 ± 0.0 / 89.0 ± 1.9	**0.43** ± 0.0 / **91.6** ± 3.5	0.86 ± 0.0 / 87.0 ± 1.7	1.08 ± 0.1 / **89.9** ± 4.2	**1.81** ± 0.0 / 87.6 ± 1.1
GCDM-Opt. (250 steps on initial samples)	**3.24** ± 0.2 / **86.6** ± 1.9	**0.93** ± 0.0 / **89.7** ± 2.2	0.43 ± 0.0 / 90.7 ± 0.0	**0.85** ± 0.0 / **88.6** ± 3.8	1.04± 0.0 / 89.5 ± 2.6	1.82 ± 0.1 / **87.6** ± 2.3

The results are reported in terms of molecular stability (MS) and the MAE for molecular property prediction by an ensemble of three EGNN classifiers $\phi_{c}$ (each trained on the same QM9 subset using a distinct random seed) yielding corresponding Student’s t-distribution 95% confidence intervals, with results listed for EDM and GCDM-optimized samples as well as the molecule generation baseline (”Initial Samples”). Note that certain experiments with an EDM optimizer yielded unsuccessful property optimization, where we denote such results as outlier property MAE values greater than 50. The top-1 (best) results for this task are in **bold**, and the second-best results are underlined.

### C.4 Reproducibility

On GitHub, we thoroughly provide all source code, data, and instructions required to train new GCDM models or reproduce our results for each of the four protein-independent molecule generation tasks we study in this work. The source code, data, and instructions for our protein-conditional molecule generation experiments are also available on GitHub. Our source code uses PyTorch [72] and PyTorch Lightning [71] to facilitate model training; PyTorch Geometric [73] to support sparse tensor operations on geometric graphs; and Hydra [74] to enable reproducible hyperparameter and experiment management.

### C.5 Additional Results

#### C.5.1 Property-Guided 3D Molecule Optimization - QM9

In Table C1, for completeness, we list the numeric molecule optimization results comprising Figure 6 in Section 2.4.

## References

[1] Rombach R., Blattmann A., Lorenz D., Esser P., Ommer B.: High-resolution image synthesis with latent diffusion models. In: Proceedings of the IEEE/CVF Conference on Computer Vision and Pattern Recognition, pp. 10684–10695 (2022)

[2] Kong Z., Ping W., Huang J., Zhao K., Catanzaro B.: Diffwave: A versatile diffusion model for audio synthesis. arXiv preprint arXiv:2009.09761 (2020)

[3] Peebles W., Radosavovic I., Brooks T., Efros A.A., Malik J.: Learning to learn with generative models of neural network checkpoints. arXiv preprint arXiv:2209.12892 (2022)

[4] Anand N., Achim T.: Protein structure and sequence generation with equivariant denoising diffusion probabilistic models. arXiv preprint arXiv:2205.15019 (2022)

[5] Corso G., Stärk H., Jing B., Barzilay R., Jaakkola T.: Diffdock: Diffusion steps, twists, and turns for molecular docking. arXiv preprint arXiv:2210.01776 (2022)

[6] Guo Z., Liu J., Wang Y., Chen M., Wang D., Xu D., Cheng J.: Diffusion models in bioinformatics and computational biology. Nature Reviews Bioengineering (2023) 10.1038/s44222-023-00114-9 PMC10994218 38576453

[7] Watson J.L., Juergens D., Bennett N.R., Trippe B.L., Yim J., Eisenach H.E., Ahern W., Borst A.J., Ragotte R.J., Milles L.F., : De novo design of protein structure and function with rfdiffusion. Nature 620(7976), 1089–1100 (2023) 37433327 10.1038/s41586-023-06415-8 PMC10468394

[8] Morehead A., Ruffolo J.A., Bhatnagar A., Madani A.: Towards joint sequence-structure generation of nucleic acid and protein complexes with se(3)-discrete diffusion. In: NeurIPS 2023 Workshop on Machine Learning in Structural Biology, p. 14 (2023)

[9] Xu M., Yu L., Song Y., Shi C., Ermon S., Tang J.: Geodiff: A geometric diffusion model for molecular conformation generation. arXiv preprint arXiv:2203.02923 (2022)

[10] Gebauer N.W., Gastegger M., Hessmann S.S., Müller K.-R., Schütt K.T.: Inverse design of 3d molecular structures with conditional generative neural networks. Nature communications 13(1), 973 (2022) 10.1038/s41467-022-28526-y PMC8861047 35190542

[11] Anstine D.M., Isayev O.: Generative models as an emerging paradigm in the chemical sciences. Journal of the American Chemical Society 145(16), 8736–8750 (2023) 37052978 10.1021/jacs.2c13467 PMC10141264

[12] Mudur N., Finkbeiner D.P.: Can denoising diffusion probabilistic models generate realistic astrophysical fields? arXiv preprint arXiv:2211.12444 (2022)

[13] Bronstein M.M., Bruna J., Cohen T., Veličković P.: Geometric deep learning: Grids, groups, graphs, geodesics, and gauges. arXiv preprint arXiv:2104.13478 (2021)

[14] Joshi C.K., Bodnar C., Mathis S.V., Cohen T., Liò P.: On the expressive power of geometric graph neural networks. arXiv preprint arXiv:2301.09308 (2023)

[15] Stärk H., Ganea O., Pattanaik L., Barzilay R., Jaakkola T.: Equibind: Geometric deep learning for drug binding structure prediction. In: International Conference on Machine Learning, pp. 20503–20521 (2022). PMLR

[16] Morehead A., Chen C., Cheng J.: Geometric transformers for protein interface contact prediction. In: 10th International Conference on Learning Representations (ICLR 2022) (2022)

[17] Jamasb A.R., Morehead A., Joshi C.K., Zhang Z., Didi K., Mathis S.V., Harris C., Tang J., Cheng J., Liò P., : Evaluating representation learning on the protein structure universe. In: 12th International Conference on Learning Representations (ICLR 2024), p. 14 (2024)

[18] Morehead A., Liu J., Cheng J.: Protein structure accuracy estimation using geometry-complete perceptron networks. Protein Science (2024) 10.1002/pro.4932 PMC10880424 38380738

[19] Jumper J., Evans R., Pritzel A., Green T., Figurnov M., Ronneberger O., Tunyasuvunakool K., Bates R., Žídek A., Potapenko A., : Highly accurate protein structure prediction with alphafold. Nature 596(7873), 583–589 (2021) 34265844 10.1038/s41586-021-03819-2 PMC8371605

[20] Vaswani A., Shazeer N., Parmar N., Uszkoreit J., Jones L., Gomez A.N., Kaiser L., Polosukhin I.: Attention is all you need. Advances in neural information processing systems 30 (2017)

[21] Lin Z., Akin H., Rao R., Hie B., Zhu Z., Lu W., Smetanin N., Verkuil R., Kabeli O., Shmueli Y., : Evolutionary-scale prediction of atomic-level protein structure with a language model. Science 379(6637), 1123–1130 (2023) 36927031 10.1126/science.ade2574

[22] Thomas N., Smidt T., Kearnes S., Yang L., Li L., Kohlhoff K., Riley P.: Tensor field networks: Rotation-and translation-equivariant neural networks for 3d point clouds. arXiv preprint arXiv:1802.08219 (2018)

[23] Buttenschoen M., Morris G.M., Deane C.M.: Posebusters: Ai-based docking methods fail to generate physically valid poses or generalise to novel sequences. Chemical Science (2024) 10.1039/d3sc04185a PMC10901501 38425520

[24] Ramakrishnan R., Dral P.O., Rupp M., Von Lilienfeld O.A.: Quantum chemistry structures and properties of 134 kilo molecules. Scientific data 1(1), 1–7 (2014) 10.1038/sdata.2014.22 PMC4322582 25977779

[25] Hoogeboom E., Satorras V.G., Vignac C., Welling M.: Equivariant diffusion for molecule generation in 3d. In: International Conference on Machine Learning, pp. 8867–8887 (2022). PMLR

[26] Anderson B., Hy T.S., Kondor R.: Cormorant: Covariant molecular neural networks. Advances in neural information processing systems 32 (2019)

[27] Satorras V.G., Hoogeboom E., Fuchs F.B., Posner I., Welling M.: E (n) equivariant normalizing flows. arXiv preprint arXiv:2105.09016 (2021)

[28] Landrum G., : Rdkit: A software suite for cheminformatics, computational chemistry, and predictive modeling. Greg Landrum 8 (2013)

[29] KrishnaR., WangJ., AhernW., SturmfelsP., VenkateshP., KalvetI., LeeG.R., Morey-BurrowsF.S., AnishchenkoI., HumphreysI.R., : Generalized biomolecular modeling and design with rosettafold all-atom. bioRxiv, 2023–10 (2023)10.1126/science.adl252838452047

[30] DeepMind-Isomorphic: Performance and structural coverage of the latest, in-development alphafold model. DeepMind (2023)

[31] Gebauer N., Gastegger M., Schütt K.: Symmetry-adapted generation of 3d point sets for the targeted discovery of molecules. Advances in neural information processing systems 32 (2019)

[32] Wu L., Gong C., Liu X., Ye M., Liu Q.: Diffusion-based molecule generation with informative prior bridges. arXiv preprint arXiv:2209.00865 (2022)

[33] Xu M., Powers A., Dror R., Ermon S., Leskovec J.: Geometric latent diffusion models for 3d molecule generation. arXiv preprint arXiv:2305.01140 (2023)

[34] Vignac C., Osman N., Toni L., Frossard P.: Midi: Mixed graph and 3d denoising diffusion for molecule generation. arXiv preprint arXiv:2302.09048 (2023)

[35] Le T., Cremer J., Noé F., Clevert D.-A., Schütt K.: Navigating the design space of equivariant diffusion-based generative models for de novo 3d molecule generation. arXiv preprint arXiv:2309.17296 (2023)

[36] Satorras V.G., Hoogeboom E., Welling M.: E (n) equivariant graph neural networks. In: International Conference on Machine Learning, pp. 9323–9332 (2021). PMLR

[37] Smith D.G., Burns L.A., Simmonett A.C., Parrish R.M., Schieber M.C., Galvelis R., Kraus P., Kruse H., Di Remigio R., Alenaizan A., : Psi4 1.4: Open-source software for high-throughput quantum chemistry. The Journal of chemical physics 152(18) (2020) 10.1063/5.0006002 PMC7228781 32414239

[38] Lehtola S., Steigemann C., Oliveira M.J., Marques M.A.: Recent developments in libxc—a comprehensive library of functionals for density functional theory. SoftwareX 7, 1–5 (2018)

[39] Pracht P., Bohle F., Grimme S.: Automated exploration of the low-energy chemical space with fast quantum chemical methods. Physical Chemistry Chemical Physics 22(14), 7169–7192 (2020) 32073075 10.1039/c9cp06869d

[40] Axelrod S., Gomez-Bombarelli R.: Geom, energy-annotated molecular conformations for property prediction and molecular generation. Scientific Data 9(1), 185 (2022) 35449137 10.1038/s41597-022-01288-4 PMC9023519

[41] Rappé A.K., Casewit C.J., Colwell K., Goddard III W.A., Skiff W.M.: Uff, a full periodic table force field for molecular mechanics and molecular dynamics simulations. Journal of the American chemical society 114(25), 10024–10035 (1992)

[42] Riniker S., Landrum G.A.: Better informed distance geometry: using what we know to improve conformation generation. Journal of chemical information and modeling 55(12), 2562–2574 (2015) 26575315 10.1021/acs.jcim.5b00654

[43] Wills S., Sanchez-Garcia R., Dudgeon T., Roughley S.D., Merritt A., Hubbard R.E., Davidson J., Delft F., Deane C.M.: Fragment merging using a graph database samples different catalogue space than similarity search. Journal of Chemical Information and Modeling (2023) 10.1021/acs.jcim.3c00276 PMC10268959 37229647

[44] Zaidi S., Schaarschmidt M., Martens J., Kim H., Teh Y.W., Sanchez-Gonzalez A., Battaglia P., Pascanu R., Godwin J.: Pre-training via denoising for molecular property prediction. arXiv preprint arXiv:2206.00133 (2022)

[45] Deore A.B., Dhumane J.R., Wagh R., Sonawane R.: The stages of drug discovery and development process. Asian Journal of Pharmaceutical Research and Development 7(6), 62–67 (2019)

[46] Hu L., Benson M.L., Smith R.D., Lerner M.G., Carlson H.A.: Binding moad (mother of all databases). Proteins: Structure, Function, and Bioinformatics 60(3), 333–340 (2005) 10.1002/prot.20512 15971202

[47] Francoeur P.G., Masuda T., Sunseri J., Jia A., Iovanisci R.B., Snyder I., Koes D.R.: Three-dimensional convolutional neural networks and a cross-docked data set for structure-based drug design. Journal of chemical information and modeling 60(9), 4200–4215 (2020) 32865404 10.1021/acs.jcim.0c00411 PMC8902699

[48] Schneuing A., Du Y., Harris C., Jamasb A.R., Igashov I., Blundell T.L., Lio P., Gomes C.P., Welling M., Bronstein M.M., : Structure-based drug design with equivariant diffusion models (2022) 10.1038/s43588-024-00737-x PMC11659159 39653846

[49] Alhossary A., Handoko S.D., Mu Y., Kwoh C.-K.: Fast, accurate, and reliable molecular docking with quickvina 2. Bioinformatics 31(13), 2214–2216 (2015) 25717194 10.1093/bioinformatics/btv082

[50] Bickerton G.R., Paolini G.V., Besnard J., Muresan S., Hopkins A.L.: Quantifying the chemical beauty of drugs. Nature chemistry 4(2), 90–98 (2012) 10.1038/nchem.1243 PMC3524573 22270643

[51] Ertl P., Schuffenhauer A.: Estimation of synthetic accessibility score of drug-like molecules based on molecular complexity and fragment contributions. Journal of cheminformatics 1, 1–11 (2009) 20298526 10.1186/1758-2946-1-8 PMC3225829

[52] Peng X., Luo S., Guan J., Xie Q., Peng J., Ma J.: Pocket2mol: Efficient molecular sampling based on 3d protein pockets. In: International Conference on Machine Learning, pp. 17644–17655 (2022). PMLR

[53] Lipinski C.A.: Lead-and drug-like compounds: the rule-of-five revolution. Drug discovery today: Technologies 1(4), 337–341 (2004) 24981612 10.1016/j.ddtec.2004.11.007

[54] Tanimoto T.T.: Elementary Mathematical Theory of Classification and Prediction. International Business Machines Corp., ??? (1958)

[55] Bajusz D., Rácz A., Héberger K.: Why is tanimoto index an appropriate choice for fingerprint-based similarity calculations? Journal of cheminformatics 7(1), 1–13 (2015) 26052348 10.1186/s13321-015-0069-3 PMC4456712

[56] Morehead A., Cheng J.: Geometry-complete perceptron networks for 3d molecular graphs. Bioinformatics (2024) 10.1093/bioinformatics/btae087 PMC10904142 38373819

[57] Ho J., Jain A., Abbeel P.: Denoising diffusion probabilistic models. Advances in Neural Information Processing Systems 33, 6840–6851 (2020)

[58] Segler M.H., Kogej T., Tyrchan C., Waller M.P.: Generating focused molecule libraries for drug discovery with recurrent neural networks. ACS central science 4(1), 120–131 (2018) 29392184 10.1021/acscentsci.7b00512 PMC5785775

[59] Jin W., Barzilay R., Jaakkola T.: Junction tree variational autoencoder for molecular graph generation. In: Dy J., Krause A. (eds.) Proceedings of the 35th International Conference on Machine Learning. Proceedings of Machine Learning Research, vol. 80, pp. 2323–2332. PMLR, ??? (2018). https://proceedings.mlr.press/v80/jin18a.html

[60] Song J., Meng C., Ermon S.: Denoising diffusion implicit models. arXiv preprint arXiv:2010.02502 (2020)

[61] Liao Y.-L., Wood B.M., Das A., Smidt T.: Equiformerv2: Improved equivariant transformer for scaling to higher-degree representations. In: The Twelfth International Conference on Learning Representations (2024). https://openreview.net/forum?id=mCOBKZmrzD

[62] Harris C., Didi K., Jamasb A.R., Joshi C.K., Mathis S.V., Lio P., Blundell T.: Benchmarking generated poses: How rational is structure-based drug design with generative models? arXiv preprint arXiv:2308.07413 (2023)

[63] Du W., Zhang H., Du Y., Meng Q., Chen W., Zheng N., Shao B., Liu T.-Y.: SE(3) equivariant graph neural networks with complete local frames. In: Chaudhuri K., Jegelka S., Song L., Szepesvari C., Niu G., Sabato S. (eds.) Proceedings of the 39th International Conference on Machine Learning. Proceedings of Machine Learning Research, vol. 162, pp. 5583–5608 (2022)

[64] Sohl-Dickstein J., Weiss E., Maheswaranathan N., Ganguli S.: Deep unsupervised learning using nonequilibrium thermodynamics. In: International Conference on Machine Learning, pp. 2256–2265 (2015). PMLR

[65] Kingma D., Salimans T., Poole B., Ho J.: Variational diffusion models. Advances in neural information processing systems 34, 21696–21707 (2021)

[66] Köhler J., Klein L., Noé F.: Equivariant flows: exact likelihood generative learning for symmetric densities. In: International Conference on Machine Learning, pp. 5361–5370 (2020). PMLR

[67] Walters W.P., Murcko M.: Assessing the impact of generative ai on medicinal chemistry. Nature biotechnology 38(2), 143–145 (2020) 10.1038/s41587-020-0418-2 32001834

[68] Urbina F., Lentzos F., Invernizzi C., Ekins S.: Dual use of artificial-intelligence-powered drug discovery. Nature Machine Intelligence 4(3), 189–191 (2022) 10.1038/s42256-022-00465-9 PMC9544280 36211133

[69] Elfwing S., Uchibe E., Doya K.: Sigmoid-weighted linear units for neural network function approximation in reinforcement learning. Neural Networks 107, 3–11 (2018) 29395652 10.1016/j.neunet.2017.12.012

[70] Loshchilov I., Hutter F.: Decoupled weight decay regularization. arXiv preprint arXiv:1711.05101 (2017)

[71] <b>Falcon, W.A.: Pytorch lightning. GitHub 3 (2019)

[72] Paszke A., Gross S., Massa F., Lerer A., Bradbury J., Chanan G., Killeen T., Lin Z., Gimelshein N., Antiga L., : Pytorch: An imperative style, high-performance deep learning library. Advances in neural information processing systems 32 (2019)

[73] Fey M., Lenssen J.E.: Fast graph representation learning with pytorch geometric. arXiv preprint arXiv:1903.02428 (2019)

[74] Yadan O.: Hydra - A framework for elegantly configuring complex applications. Github (2019). https://github.com/facebookresearch/hydra

