# Supplementary material for: Geometry-Complete Diffusion for 3D Molecule Generation and Optimization
Source: ArXiv. 2024 Feb 5:arXiv:2302.04313v5. Originally published 2023 Feb 8. Preprint. [Version 5] (PMC9934735)
Supplement: Supplementary file 1 [file NIHPP2302.04313V5-supplement-1.pdf]

Appendix A Expanded Discussion of Methodology

A.1 Diffusion Models

Key to understanding our contributions in this work are denoising diffusion probabilistic models (DDPMs). As alluded to previously, once trained, DDPMs can generate new data of arbitrary shapes, sizes, formats, and geometries by learning to reverse a noising process acting on each model input. More precisely, for a given data point $\mathbf{x}$, a diffusion process adds noise to $\mathbf{x}$ for time step $t = 0, 1, ..., T$ to yield $\mathbf{z}_t$, a noisy representation of the input $\mathbf{x}$ at time step t. Such a process is defined by a multivariate Gaussian distribution:

$$q(\mathbf{z}_t|x) = \mathcal{N}(\mathbf{z}_t|\alpha_t \mathbf{x}_t, \sigma_t^2 \mathbf{I}), \tag{A1}$$

where $\alpha_t \in \mathbb{R}^+$ regulates how much feature signal is retained and σ_t^2 modulates how much feature noise is added to input $\mathbf{x}$. Note that we typically model α as a function defined with smooth transitions from $\alpha_0 = 1$ to $\alpha_T = 0$, where a special case of such a noising process, the variance preserving process [34, 42], is defined by $\alpha_t = \sqrt{1 - \sigma_t^2}$. To simplify notation, in this work, we define the feature signal-to-noise ratio as $\text{SNR}(t) = \alpha_t^2/\sigma_t^2$. Also interesting to note is that this diffusion process is Markovian in nature, indicating that we have transition distributions as follows:

$$q(\mathbf{z}_t|\mathbf{z}_s) = \mathcal{N}(\mathbf{z}_t|\alpha_{t|s}\mathbf{z}_s, \sigma_{t|s}^2 \mathbf{I}), \tag{A2}$$

for all $t > s$ with $\alpha_{t|s} = \alpha_t/\alpha_s$ and $\sigma_{t|s}^2 = \sigma_t^2 - \alpha_{t|s}^2 \sigma_s^2$. In total, then, we can write the noising process as:

$$q(\mathbf{z}_0, \mathbf{z}_1, ..., \mathbf{z}_T|\mathbf{x}) = q(\mathbf{z}_0|x) \prod_{t=1}^{T} q(\mathbf{z}_t|\mathbf{z}_{t-1}). \tag{A3}$$

If we then define $\boldsymbol{\mu}_{t\to s}(\mathbf{x}, \mathbf{z}_t)$ and $\sigma_{t\to s}$ as

$$\boldsymbol{\mu}_{t\to s}(\mathbf{x}, \mathbf{z}_t) = \frac{\alpha_{t|s}\sigma_s^2}{\sigma_t^2}\mathbf{z}_t + \frac{\alpha_s \sigma_{t|s}^2}{\sigma_t^2}\mathbf{x} \quad \text{and} \quad \sigma_{t\to s} = \frac{\sigma_{t|s}\sigma_s}{\sigma_t},$$

we have that the inverse of the noising process, the *true denoising process*, is given by the posterior of the transitions conditioned on $\mathbf{x}$, a process that is also Gaussian:

$$q(\mathbf{z}_s|\mathbf{x}, \mathbf{z}_t) = \mathcal{N}(\mathbf{z}_s|\boldsymbol{\mu}_{t\to s}(\mathbf{x}, \mathbf{z}_t), \sigma_{t\to s}\mathbf{I}). \tag{A4}$$

The Generative Denoising Process. In diffusion models, we define the generative process according to the *true denoising process*. However, for such a denoising process, we do not know the value of $\mathbf{x}$ *a priori*, so we typically approximate it as $\hat{\mathbf{x}} = \phi(\mathbf{z}_t, t)$ using a neural network ϕ. Doing so then lets us express the generative transition distribution $p(\mathbf{z}_s|\mathbf{z}_t)$ as $q(\mathbf{z}_s|\hat{\mathbf{x}}(\mathbf{z}_t, t), \mathbf{z}_t)$. As a practical alternative to Eq. A4, we can represent this expression using our approximation for $\hat{\mathbf{x}}$:

$$p(\mathbf{z}_s|\mathbf{z}_t) = \mathcal{N}(\mathbf{z}_s|\boldsymbol{\mu}_{t\to s}(\hat{\mathbf{x}}, \mathbf{z}_t), \sigma_{t\to s}^2 \mathbf{I}). \tag{A5}$$

If we choose to define s as $s = t - 1$, then we can derive the variational lower bound on the log-likelihood of $\mathbf{x}$ given our generative model as:

$$\log p(\mathbf{x}) \geq \mathcal{L}_0 + \mathcal{L}_{base} + \sum_{t=1}^{T} \mathcal{L}_t, \tag{A6}$$

where we note that $\mathcal{L}_0 = \log p(\mathbf{x}|\mathbf{z}_0)$ models the likelihood of the data given its noisy representation $\mathbf{z}_0$, $\mathcal{L}_{base} = -\mathrm{KL}(q(\mathbf{z}_T|\mathbf{x})|p(\mathbf{z}_T))$ models the difference between a standard normal distribution and the final latent variable $q(\mathbf{z}_T|\mathbf{x})$, and

$$\mathcal{L}_t = -\mathrm{KL}(q(\mathbf{z}_s|\mathbf{x}, \mathbf{z}_t)|p(\mathbf{z}_s|\mathbf{z}_t)) \quad \text{for } t = 1, 2, ..., T.$$

Note that, in this formation of diffusion models, our neural network ϕ directly predicts $\hat{\mathbf{x}}$. However, Ho et al. [34] and others have found optimization of ϕ to be made much easier when instead predicting the Gaussian noise added to $\mathbf{x}$ to create $\hat{\mathbf{x}}$. An intuition for how this changes the neural network's learning dynamics is that, when predicting back the noise added to the model's input, the network is being trained to more directly differentiate which part of $\mathbf{z}_t$ corresponds to the input's feature signal (i.e., the underlying data point $\mathbf{x}$) and which part corresponds to added feature noise. In doing so, if we let $\mathbf{z}_t = \alpha_t \mathbf{x} + \sigma_t \boldsymbol{\epsilon}$, our neural network can then predict $\hat{\boldsymbol{\epsilon}} = \phi(\mathbf{z}_t, t)$ such that:

$$\hat{\mathbf{x}} = (1/\alpha_t)\ \mathbf{z}_t - (\sigma_t/\alpha_t)\ \hat{\boldsymbol{\epsilon}}. \tag{A7}$$

Kingma et al. [43] and others have since shown that, when parametrizing our denoising neural network in this way, the loss term $\mathcal{L}_t$ reduces to:

$$\mathcal{L}_t = \mathbb{E}_{\boldsymbol{\epsilon} \sim \mathcal{N}(\mathbf{0}, \mathbf{I})} \left[\frac{1}{2}(1 - \mathrm{SNR}(t-1)/\mathrm{SNR}(t)) \|\boldsymbol{\epsilon} - \hat{\boldsymbol{\epsilon}}\|^2 \right] \tag{A8}$$

Note that, in practice, the loss term $\mathcal{L}_{base}$ should be close to zero when using a noising schedule defined such that $\alpha_T \approx 0$. Moreover, if and when $\alpha_0 \approx 1$ *and* $\mathbf{x}$ is a discrete value, we will find $\mathcal{L}_0$ to be close to zero as well.

A.2 Zeroth Likelihood Terms for GCDM Optimization Objective

For the zeroth likelihood terms corresponding to each type of input feature, we directly adopt the respective terms previously derived by Hoogeboom et al. [20]. Doing so enables a negative log-likelihood calculation for GCDM's predictions. In particular, for integer node features, we adopt the zeroth likelihood term:

$$p(\mathbf{h}|\mathbf{z}_0^{(h)}) = \int_{\mathbf{h}-\frac{1}{2}}^{\mathbf{h}+\frac{1}{2}} \mathcal{N}(\mathbf{u}|\mathbf{z}_0^{(h)}, \sigma_0) d\mathbf{u}, \tag{A9}$$

where we use the CDF of a standard normal distribution, Φ, to compute Eq. A9 as $\Phi((\mathbf{h} + \frac{1}{2} - \mathbf{z}_0^{(h)})/\sigma_0) - \Phi((\mathbf{h} - \frac{1}{2} - \mathbf{z}_0^{(h)})/\sigma_0) \approx 1$ for reasonable noise parameters α_0

and σ_0 [20]. For categorical node features, we instead use the zeroth likelihood term:

$$p(\mathbf{h}|\mathbf{z}_0^{(h)}) = C(\mathbf{h}|\mathbf{p}), \mathbf{p} \propto \int_{1-\frac{1}{2}}^{1+\frac{1}{2}} \mathcal{N}(\mathbf{u}|\mathbf{z}_0^{(h)}, \sigma_0)\mathrm{d}\mathbf{u}, \tag{A10}$$

where we normalize $\mathbf{p}$ to sum to one and where C is a categorical distribution [20]. Lastly, for continuous node positions, we adopt the zeroth likelihood term:

$$p(\mathbf{x}|\mathbf{z}_0^{(x)}) = \mathcal{N}\left(\mathbf{x}|\mathbf{z}_0^{(x)}/\alpha_0 - \sigma_0/\alpha_0\hat{\boldsymbol{\epsilon}}_0, \sigma_0^2/\alpha_0^2\mathbf{I}\right) \tag{A11}$$

which gives rise to the log-likelihood component $\mathcal{L}_0^{(x)}$ as:

$$\mathcal{L}_0^{(x)} = \mathbb{E}_{\boldsymbol{\epsilon}^{(x)} \sim \mathcal{N}_x(\mathbf{0},\mathbf{I})}\left[\log Z^{-1} - \frac{1}{2}\|\boldsymbol{\epsilon}^x - \phi^{(x)}(\mathbf{z}_0, 0)\|^2\right], \tag{A12}$$

where $d = 3$ and the normalization constant $Z = (\sqrt{2\pi} \cdot \sigma_0/\alpha_0)^{(N-1)\cdot d}$ - in particular, its $(N-1)\cdot d$ term - arises from the zero center of gravity trick mentioned in Section 3.4 of the main text [20].

A.3 Diffusion Models and Equivariant Distributions

In the context of diffusion generative models of 3D data, one often desires for the marginal distribution $p(\mathbf{x})$ of their denoising neural network to be an invariant distribution. Towards this end, we observe that a conditional distribution $p(y|x)$ is equivariant to the action of 3D rotations by meeting the criterion:

$$p(y|x) = p(\mathbf{R}y|\mathbf{R}x) \quad \text{for all orthogonal } \mathbf{R}. \tag{A13}$$

Moreover, a distribution is invariant to rotation transformations $\mathbf{R}$ when

$$p(y) = p(\mathbf{R}y) \quad \text{for all orthogonal } \mathbf{R}. \tag{A14}$$

As Köhler et al. [44] and Xu et al. [6] have collectively demonstrated, we know that if $p(\mathbf{z}_T)$ is invariant and the neural network we use to parametrize $p(\mathbf{z}_{t-1}|\mathbf{z}_t)$ is equivariant, we have, as desired, that the marginal distribution $p(\mathbf{x})$ of the denoising model is an invariant distribution.

A.4 Training and Sampling Procedures for GCDM

Equivariant Dynamics. In this work, we use our previous definition of GCPNet++ in Section 3.4 of the main text to learn an SE(3)-equivariant dynamics function $[\hat{\boldsymbol{\epsilon}}^{(x)}, \hat{\boldsymbol{\epsilon}}^{(h)}] = \phi(\mathbf{z}_t^{(x)}, \mathbf{z}_t^{(h)}, t)$ as:

$$\hat{\boldsymbol{\epsilon}}_t^{(x)}, \hat{\boldsymbol{\epsilon}}_t^{(h)} = \text{GCPNet++}(\mathbf{z}_t^{(x)}, [\mathbf{z}_t^{(h)}, t/T]) - [\mathbf{z}_t^{(x)}, \mathbf{0}], \tag{A15}$$

where we inform our denoising model of the current time step by concatenating t/T as an additional node feature and where we subtract the coordinate representation outputs of GCPNet++ from its coordinate representation inputs after subtracting from the coordinate representation outputs their collective center of gravity. With the parametrization in Eq. 8 of the main text, GCDM subsequently achieves rotation equivariance on $\hat{\mathbf{x}}_i$, thereby achieving a 3D translation and rotation-invariant marginal distribution $p(\mathbf{x})$ as described in Appendix A.3.

Scaling Node Features. In line with Hoogeboom et al. [20], to improve the log-likelihood of our model's generated samples, we find it useful to train and perform sampling with GCDM using scaled node feature inputs as $[\mathbf{x}, \frac{1}{4}\mathbf{h}^{(categorical)}, \frac{1}{10}\mathbf{h}^{(integer)}]$.

Deriving The Number of Atoms. Finally, to determine the number of atoms with which GCDM will generate a 3D molecule, we first sample $N \sim p(N)$, where $p(N)$ denotes the categorical distribution of molecule sizes over GCDM's training dataset. Then, we conclude by sampling $\mathbf{x}, \mathbf{h} \sim p(\mathbf{x}, \mathbf{h}|N)$.

Appendix B Additional Details

B.1 Broader Impacts

In this work, we investigate the impact of geometric representation learning on generative models for 3D molecules. Such research can contribute to drug discovery efforts by accelerating the development of new medicinal or energy-related molecular compounds, and, as a consequence, can yield positive societal impacts [45]. Nonetheless, in line with Urbina et al. [46], we authors would argue that it will be critical for institutions, governments, and nations to reach a consensus on the strict regulatory practices that should govern the use of such molecule design methodologies in settings in which it is reasonably likely such methodologies could be used for nefarious purposes by scientific "bad actors".

B.2 Training Details

Scalar Message Attention. In our implementation of scalar message attention (SMA) within GCDM, $\mathbf{m}_{ij} = e_{ij}\mathbf{m}_{ij}$, where $\mathbf{m}_{ij}$ represents the scalar messages learned by GCPNet++ during message-passing and e_{ij} represents a 1 if an edge exists between nodes i and j (and a 0 otherwise) via $e_{ij} \approx \phi_{inf}(\mathbf{m}_{ij})$. Here, $\phi_{inf} : \mathbb{R}^e \rightarrow [0, 1]^1$ resembles a linear layer followed by a sigmoid function [23].

GCDM Hyperparameters. All GCDM models train on QM9 for approximately 1,000 epochs using 9 **GCPConv** layers; SiLU activations [47]; 256 and 64 scalar node and edge hidden features, respectively; and 32 and 16 vector-valued node and edge features, respectively. All GCDM models are also trained using the AdamW optimizer [48] with a batch size of 64, a learning rate of 10^{-4}, and a weight decay rate of 10^{-12}.

GCDM Runtime. With a maximum batch size of 64, this 9-layer model configuration allows us to train GCDM models for unconditional (conditional) tasks on the QM9 dataset using approximately 10 (15) days of GPU training time with a single 24GB NVIDIA A10 GPU. For unconditional molecule generation on the much larger GEOM-Drugs dataset, a maximum batch size of 64 allows us to train 4-layer GCDM models using approximately 60 days of GPU training time with a single 48GB NVIDIA RTX A6000 GPU. As such, access to several GPUs with larger GPU memory limits (e.g., 80GBs) should allow one to concurrently train GCDM models in a fraction of the time via larger batch sizes or data-parallel training techniques [49].

B.3 Compute Requirements

Training GCDM models for tasks on the QM9 dataset by default requires a GPU with at least 24GB of GPU memory. Inference with such GCDM models for QM9 is much more flexible in terms of GPU memory requirements, as users can directly control how soon a molecule generation batch will complete according to the size of molecules being generated as well as one's selected batch size during sampling. Training GCDM models for unconditional molecule generation on the GEOM-Drugs dataset by default requires a GPU with at least 48GB of GPU memory. Similar to our GCDM models for QM9, inference with GEOM-Drugs models is flexible in terms of GPU memory requirements according to one's choice of sampling hyperparameters. Note that inference for both

QM9 models and GEOM-Drugs models can likely be accelerated using techniques such as DDIM sampling [40]. However, we have not officially validated the quality of generated molecules using such sampling techniques, so we caution users to be aware of this potential risk of degrading molecule sample quality when using such sampling algorithms.

B.4 Reproducibility

On GitHub, we thoroughly provide all source code, data, and instructions required to train new GCDM models or reproduce our results for each of the four molecule generation tasks we study in this work. Our source code uses PyTorch [50] and PyTorch Lightning [49] to facilitate model training; PyTorch Geometric [51] to support sparse tensor operations on geometric graphs; and Hydra [52] to enable reproducible hyperparameter and experiment management.
